# Supplementary material for: Dichloromethylation of enones by carbon nitride photocatalysis
Source: Nat Commun. 2020 Mar 13;11:1387. doi: 10.1038/s41467-020-15131-0 (PMC7070069; doi:10.1038/s41467-020-15131-0)
Supplement: Supplementary file 1 — Supplementary Information [file 41467_2020_15131_MOESM1_ESM.pdf]

Supplementary Information

Dichloromethylation of Enones by Carbon Nitride Photocatalysis

Stefano Mazzanti[1,†], Bogdan Kurpil[1,†], Bartholomäus Pieber[2], Markus Antonietti[1] and Aleksandr Savateev[1]*

[1] Max-Planck Institute of Colloids and Interfaces, Department of Colloid Chemistry, Research Campus Golm, Am Mühlenberg 1, 14476 Potsdam, Germany.

[2] Max Planck Institute of Colloids and Interfaces, Department of Biomolecular Systems Am Mühlenberg 1, 14476 Potsdam, Germany.

[*] Corresponding author. E-mail address: oleksandr.savatieiev@mpikg.mpg.de (A. Savateev).

† authors contributed equally.

Supplementary Methods

^{1}H and ^{13}C NMR spectra were recorded on Agilent 400 MHz (at 400 MHz for Protons and 101 MHz for Carbon-13). Chemical shifts are reported in ppm versus solvent residual peak: chloroform-*d* 7.26 ppm (^{1}H NMR), 77.1 ppm (^{13}C NMR); acetonitrile-d_3 1.94 ppm (^{1}H NMR), 118.3 ppm (^{13}C NMR).

Chlorine elemental analysis was performed by company MikroLab.

Irradiance of the LED modules was measured using PM400 Optical Power and Energy Meter equipped with integrating sphere S142C.

Powder X-Ray diffraction patterns were measured on a Bruker D8 Advance diffractometer equipped with a scintillation counter detector with CuKα radiation (λ = 0.15418 nm) applying 2θ step size of 0.05° and counting time of 3s per step.

Nitrogen adsorption/desorption measurements were performed after degassing the samples at 150 °C for 20 hours using a Quantachrome Quadrasorb SI-MP porosimeter at 77.4 K. The specific surface areas were calculated by applying the Brunauer-Emmett-Teller (BET) model to adsorption isotherms for $0.05 < p/p_0 < 0.3$ using the QuadraWin 5.11 software package.

Mass spectral data were obtained using Agilent GC 6890 gas chromatograph, equipped wit HP-5MS column (inner diameter=0.25 mm, length=30 m, and film=0.25 µm), coupled with Agilent MSD 5975 mass spectrometer (electron ionization).

Scanning electron microscopy (SEM) images were obtained on a LEO 1550-Gemini microscope.

The X-ray photoelectron spectroscopy (XPS) measurements were carried out in an ultrahigh vacuum (UHV) spectrometer equipped with a VSW Class WA hemispherical electron analyzer. A dual anode Al Kα X-ray source (1486.6 eV) was used as incident radiation. Survey and high resolution spectra were recorded in constant pass energy mode (44 and 22 eV, respectively). During the UPS (He I excitation energy $h\nu$=21.23 eV) measurements a bias of 15.32 V was applied to the sample, in order to avoid interference of the spectrometer threshold in the UP spectra. The values of the valence band maximum (VBM) are determined by fitting a straight line into the leading edge.

Optical absorbance spectra of powders were measured on a Shimadzu UV 2600 equipped with an integrating sphere.

Emission spectra were recorded on Jasco FP-8300 instrument. The excitation wavelength was 360 nm.

Energy dispersive X-Ray (EDX) analysis was performed on JEOL JSM-7500F electron microscope equipped with two Oxford Instruments EDX detectors, located at opposite sides from the sample. The angle between the sample film surface and EDX detector axis was 28°.

The TEM measurements were acquired using a double-corrected Jeol ARM200F, equipped with a cold field emission gun and a Gatan GIF Quantum. The used acceleration voltage was 200kV and the emission was set to 10µA in order to reduce beam damage. An objective aperture with a diameter of 60µm was introduced into the beam to improve the contrast while still allowing for atomic resolution.

Microwave reactions were carried out in a CEM Discover® SP Systemmicrowave reactor.

Cyclic voltammetry (CV) measurements were performed in a glass single-compartment electrochemical cell. Glassy carbon (diameter 3 mm) was used as a working electrode (WE), Ag wire in $AgNO_3$ (0.01M) with tetrabutylammonium perchlorate (0.1M) in MeCN as a reference electrode (RE), Pt wire as a counter electrode. Each compound was studied in a 50 mM concentration in a 0.1 M tetrabutylammonium perchlorate (TBAP)/chloroform electrolyte solution (10 mL). Before voltammograms were recorded, the solution was purged with Ar, and an Ar flow was kept in the headspace volume of the electrochemical cell during CV measurements. A potential scan rate of 0.050 V s^{-1} was chosen, and the potential window ranging from +2.5 V to −2.5 V (and backwards) was investigated. Cyclic voltammetry was performed under room-temperature conditions (~20-22 °C).

Zeta-potentials were measured in aqueous colloidal solution of K-PHI using a Malvern Zetasizer instrument.

Hydrodynamic diameter of K-PHI particles in water was measured using Malvern Zetasizer instrument.

Light scattering by K-PHI suspension

Light scattering by K-PHI particles has been evaluated using two methods. In the first method transmittance spectrum of K-PHI suspension (2.5 mg mL^{-1}) in $CHCl_3$:DMSO (9:1) have been acquired in the range of wavelength 400-1100 nm using a UV-vis spectrometer T70+. Four cuvettes made of optical glass with optical path 1 mm, 2 mm, 5 mm and 10 mm were first filled with $CHCl_3$:DMSO (9:1) mixture and used to acquire the baseline. Solvent was removed and cuvettes were refilled with K-PHI suspension in $CHCl_3$:DMSO (9:1). Transmittance of the suspension at 461 nm was used in the discsussion.

In the second method, transmittance of K-PHI suspension (2.5 mg mL^{-1}) in $CHCl_3$:DMSO (9:1) was calculated by measuring the light intensity passed through the cuvette using blue light (461±20 nm, I_0 = 10.6 mW cm^{-2}) from the external light source. Light source from Jasco FP-8300 fluorescence spectrometer was used for this purpose. Exitation and emission bandwiths were set to 20 nm. Transmittance of the K-PHI suspension was calculated as $I/I_s \cdot 100\%$, where I – light intensity measured by the optical power meter after light passed throught the cuvette filled with K-PHI suspension, mW cm^{-2}; I_s – light intensity measured by the optical power meter after light passed through the cuvette filled with K-PHI $CHCl_3$:DMSO (9:1) mixture, mW cm^{-2}. Supplmentary Figure 86 shows the experimental setup for measurement of light intensity using a 5 mm cuvette as an example.

Flow photoreactor

Design of the flow photoreactor, i.e. tubings and fittings, used in this work is based on the reported in the literature.[1]

The flow photoreactor consist of the following parts:

A. Light source. Self-adhesive LED stripe (Luminous Flux 1820 lm/m, distance between individual LEDs 8.4 mm, Input Voltage 12V DC, Current 4 A, Power 48 W, dominant wavelength 460 nm, length 500 mm) fabricated by JKL Components (Manufactrurer Part No. ZFS-85000HD-B) were used as a light source. Three LED stripes were attached with the displacement between the leading ends of stripes ca. 120° to the home-made hollow steel cylinder (external diameter 140 mm, height 230 mm, wall thickness 1 mm) equipped with inlet and outlet (both with external diameter 8 mm, wall thickness 1 mm) for supplying cooling water. Each piece of the LED stripe is powered by a constant voltage LED driver (manufacturer Mean Well, Manufacturer Part No. LPV-60-12, Output voltage 12 V, Output current 5 A, Maximum output power 60 W). Total length of the LED stripe is 12.22 m, total number of LEDs 1437, output power at zero distance from the light source 18.5 mW cm^{-2} (Supplementary Figure 87).

B. Photoreactor. Photoreactor was made by wrapping the tubing (tetrafluorethylene/hexafluorpropylene copolymer (FEP), manufacturer Bohlender, Manufactrurer Part No. S1815-08, external diameter 3.2 mm (1/8''), internal diameter 1.6 mm (1/16'')) around the glass beaker (purchased from Fischerbrand Catalogue No. FB33119, external diameter 170 mm, height 270 mm, capacity 5000 mL). Total length of the reactor is 35 m, volume 70 mL (Supplementary Figure 88).

C. Cooling module. Huber Ministatic cc (Manufacturer Peter Huber Kältemaschinen GmbH, Manufacturer Part No. 37791/98, temperature range —25°C…+125°C) was used.

D. External shell (Supplementary Figure 89). External shell of the photoreactor was made by wrapping aluminium foil around a glass beaker (purchased from Fischerbrand Catalogue No. FB33183, external diameter 217 mm, height 350 mm, capacity 10000 mL).

E. Reaction mixture supply module (Supplementary Figure 90). The module consists of Syringe pump (World Precision Instruments, Model No. AL-1600), 4 Luer Lock Syringes (Capacity 20 mL, manufacturer B. Braun, Manufacturer part No. 4606205V), 4 Luer Adapters (1/4-28 Female to Female Luer, Material Tefzel, Manufacturer Upchurch Scientific, Manufacturer Part No. UPP-678), 3 Tee Assemblies (for tubing 1/16'', Material Tefzel, Manufacturer Upchurch Scientific, Manufacturer Part No. UPP-632), 8 Ferrules (for tubing 1/16'', 1/4-28, Material Tefzel, Manufacturer Upchurch Scientific, Manufacturer Part No. UPP-200N), 8 Nuts (for tubing 1/16'', 1/4-28, Material Tefzel, Manufacturer Upchurch Scientific, Manufacturer Part No. UPP-245), 1 Ferrule (for tubing 1/8'', 1/4-28, Material Tefzel, Manufacturer Upchurch Scientific, Manufacturer Part No. UPP-300N), 1 Nut (for tubing 1/8'', 1/4-28, Material Tefzel, Manufacturer Upchurch Scientific, Manufacturer Part No. UPP-345), 6 pieces of FEP tubing (tetrafluorethylene/hexafluorpropylene copolymer (FEP), manufacturer Bohlender, Manufactrurer Part No. S 1815-04, external diameter 3.2 mm (1/16''), internal diameter 1.6 mm (1/32''), total length ca. 500 mm), 1 piece of FEP tubing (tetrafluorethylene/hexafluorpropylene copolymer (FEP), manufacturer Bohlender, Manufactrurer Part No. S1815-08, external diameter 3.2 mm (1/8''), internal diameter 1.6 mm (1/16''), length ca. 500 mm).

F. Photocatalyst supply module (Supplementary Figure 91). The module consists of Syringe pump (KD Scientific, Model No. 789100B), Magnetic stirrer (IKA RCT classic), PTFE coated magnetic stir bar (Length 15 mm, Diameter 5 mm, manufacturer Bohlender, Manufactrurer Part No. C354-08), 1 Luer Lock Syringe (Capacity 20 mL, manufacturer B. Braun, Manufacturer part No. 4606205V), 1 T-connector (Manufacturer Kinesis, Manufacturer Part No. 001109, Material PTFE), 1 Luer adaptor (Female Luer to 1/4-28, Material Tefzel, Manufacturer Upchurch Scientific, Manufacturer Part No. P-624), 2 Ferrules (for tubing 1/8'', 1/4-28, Material Tefzel, Manufacturer Upchurch Scientific, Manufacturer Part No. UPP-300N), 2 Nuts (for tubing 1/8'', 1/4-28, Material Tefzel, Manufacturer Upchurch Scientific, Manufacturer Part No. UPP-345).

G. Reaction mixture receiver. Glass vials or flask.

Photoreactor assembly.

Cooling water inlet of the light source (A) was connected to the cooling module outlet (C) using PVC tubing (inner diameter 8 mm, outer diameter 12 mm, length ca. 1.5 m, Purchased from Carl Roth GmbH & Co. KG). Outlet of the light source (A) was connected to the cooling module inlet (C) using PVC tubing (inner diameter 8 mm, outer diameter 12 mm, length ca. 5 m, Purchased from Carl Roth GmbH & Co. KG). Longer piece of the PVC tubing was wrapped around the photoreactor

(B). The photoreactor (B) and the PVC tubing was placed inside the external shell (C). Deionized water was added to the space between the external shell (C) and the photoreactor (B) until all FEP tubing of the photoreactor (B) is completely covered with water. Outlet of the photoractor (B) was placed into the reaction mixture receiver (G). Inlet of the photoreactor (B) was connected to the outlet of the photocatalyst supply module (F). Inlet of the photocatalyst supply module (F) was connected to the outlet of the reaction mixture supply module (E).

Synthesis of dichloroketone 2a in flow photoreactor.

The syringes of the reaction mixture supply module (E) were first filled with a homogeneous solution prepared by mixing 520 mg of chalcone **2a**, 3.35 mL of TEOA in 100 mL of $CHCl_3$/DMSO (6:4) solution. The syringe of the photocatalyst supply module (F) was filled with a dispersion of K-PHI in DMSO (10 mg/mL), previously sonicated for 20 minutes for optimal dispersion. Stirring bar was added to the syringe. The syringe was fixed into the syringe pump. The stirring plate was turned on in order to maintain K-PHI suspended. The cooling system was turned on, while the temperature was set to 20 °C. The space between the photoreactor (B) and external shell (D) was filled with deionized water. The light source (A) was turned on. The flow rate of the reaction mixture supply module (E) syringe pump was set to 8 mL h^{-1} and the pump was enabled. When the reaction mixture solution reached the T-connector of the photocatalyst supply module (F), the flow rate of the syringe pump was set to 2 mL h^{-1} and the pump was enabled. After passing through the flowreactor (reaction time of 7 hours), the solution was collected into glass vials for 1 h 30 min. Fractions between minutes 30 and 60 were combined and analyzed by GC-MS to give 57% yield.

Carbon nitride photocatalysts synthesis and characterization

K-PHI

Potassium poly(heptazine imide) (K-PHI) was synthesized according to the previously described procedure.[2] Mixture of lithium chloride (3.71 g), potassium chloride (4.54 g) and 5-aminotetrazole (1.65 g) was grinded in ball mill for 5 min at the shaking rate 25 s^{-1}. Reaction mixtures were transferred into porcelain crucibles and covered with lids. Crucibles were placed in the oven and heated under constant nitrogen flow (15 L·min^{-1}) and atmospheric pressure at a following temperature regime: heating from room temperature to 550 °C for 4 hours, annealing at 550 °C for 4 hours. After completion of the heating program, the crucibles were allowed to cool slowly to room temperature under nitrogen flow. The crude products were removed from the crucibles,

washed with deionized water (100 mL) for 3 hours in order to remove salts, then filtered, extensively washed with deionized water and dried in a vacuum oven (20 mbar) at 50 °C for 15 h.

mpg-CN

Cyanamide (3.0 g) and Ludox HS-40 (7.5 g) were mixed in a 10 mL glass vial. The mixture was stirred at room temperature for 30 min until cyanamide has completely dissolved. The resultant solution was stirred at +60°C for 16 h until water has completely evaporated. Magnetic stir bar was removed and white solid was transferred to the porcelain crucible and heated under N_2 flow in the oven. The temperature was increased from room temperature to 550°C within 4 h and maintained at 550°C for 4 h. The crucible was spontaneously cooled to room temperature. The solid from the crucible was briefly grinded in the mortar and transferred to the polypropylene bottle. A solution of $(NH_4)HF_2$ (0.24 g·mL^{-1}, 50 mL) was added and suspension was stirred at room temperature for 24 h. The solid was filtered, thoroughly washed with water, once with ethanol and dried in vacuum (55°C, 20 mbar) overnight.

g-C_3N_4

Diacyandiamide (15.0 g) was calcined at 600ºC for 4 h under flow of nitrogen (15 L min^{-1}) in a porcelain crucible. Yellow solid was ground in mortar.

General method for chalcone (1a-j) preparation

To a mixture of acetophenone (1g, 8.3mmol) and corresponding aldehyde (9.1mmol) in 50 mL of mixture methanol/water (1:4), solution of sodium hydroxide (667mg, 16.6mmol) in 2 ml of water was added dropwise at room temperature and resulted solution was stirred for 20 hours at room temperature, in case of chalcones 1c - for 20 hours at reflux. Then reaction mixture was cooled to room temperature and for chalcones 1a-f,h 50ml of water was added, obtained precipitate was filtered, washed with water (3 x 15ml) then with 10ml of mixture water/ethanol (4:1) and dried on air to give product as solid material. In case of chalcones 1g, precipitate after filtration was redisolved in 30ml of mixture ethanol and aq. (2N) hydrochloride acid (1:1) and refluxed for 2 hours, then cooled to room temperature, dilluted with 50ml of water and obtained precipitete was filtered, washed with water (3 x 15ml) then with 10ml of mixture water/ethanol (4:1) then dried on air to give product as solid material.

Preparation of chalkone 1k

To stirring mixture of benzaldehyde (12g, 0.11mol) and acetone (40g, 0.68mol) in water (20ml), aquaeous (20%) solution of sodium hydroxide (1.5ml) was added dropwise at 25°C. after addition reaction mixture was stirred for another 2 hours at 25°C. Then mixture was dilluted with water (100ml) and acidified with aq. (2N) hydrochloride acid to pH=5, then extracted with chloroform (3 x 15ml), all organic solutions were combined, washed with brine (2 x 10ml), dried over Na_2SO_4. Residue after evaporation was redisolved in 50ml of mixture ethanol and aq. (2N) hydrochloride acid (1:1) and refluxed for 2 hours. Then cooled to room temperature, dilluted with 50ml of water, extracted with chloroform (3 x 15ml), all organic solutions were combined, washed with brine (2 x 10ml), dried over Na_2SO_4, evaporated to give as residue orange oil, vacuum distilation of which gives pure chalcone 1k (yield 74%).

General method for chalcone (1m, n, o, p, q) preparation

To a 100 mL flask, the corresponding aldehyde (10,4 mmol) and acetophenone (10.5 mmol, 1.22 mL) are stirred in 30 mL of EtOH. Then the solutions is ketps at 0 °C with an ice bath and slowly added an acqueous solution of NaOH (0.8g in 30 mL). The solution is then stirred at room temperature for 3 hours. The precipited formed is then filtered, washed with water and ethanol. Finally the solid obtained is purified by ricristalizzation in ethanol.

Preparation of chalkone 1r

In a flask charged with CH_3OH (0.5 mL), NaOH (1 N, 5.8 mL) and picolinaldehyde (1.80 mL, 1.0 equiv.) at 0 ℃, it was added acetophenone (1.1 mL, 0.5 equiv). The solution was left to react overnight. Then ice water (10 mL) was added and the mixture was stirred vigorously. The resulting solid was filtered, washed with cold water, dried and purified by silica gel column chromatography (EtOAc/hexanes) to provide compound 1o (1.4 g, 76% yield) as a pale yellow solid.

(E)-chalcone[3] (1a).

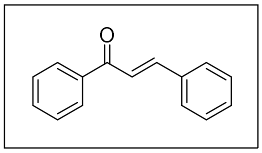

Yellowish solid (yield 73%). ^{1}H NMR (400 MHz, $CDCl_3$) δ 8.03 (dd, *J* = 8.3, 1.3 Hz, 2H), 7.82 (d, *J* = 15.7 Hz, 1H), 7.65 (dd, *J* = 6.8, 2.8 Hz, 2H), 7.60 (t, *J* = 7.3 Hz, 1H), 7.57 – 7.49 (m, 3H), 7.45 – 7.40 (m, 3H). ^{13}C NMR (101 MHz, $CDCl_3$) δ 190.5, 144.8, 138.2, 134.9, 132.8, 130.5, 128.9, 128.6, 128.5, 128.4, 122.1.

(E)-1-phenyl-3-(p-tolyl)prop-2-en-1-one[4] *(1b).*

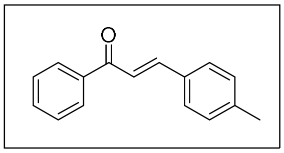

Yellowish solid (yield 82%). ^{1}H NMR (400 MHz, Chloroform-*d*) δ 8.05 – 8.00 (m, 2H), 7.81 (d, *J* = 15.7 Hz, 1H), 7.62 – 7.47 (m, 6H), 7.23 (d, *J* = 8.0 Hz, 2H), 2.40 (s, 3H). ^{13}C NMR (101 MHz, $CDCl_3$) δ 190.7, 145.0, 141.1, 138.3, 132.7, 132.1, 129.7, 128.6, 128.5, 128.5, 121.1, 21.6.

(E)-3-(4-methoxyphenyl)-1-phenylprop-2-en-1-one[3] *(1c).*

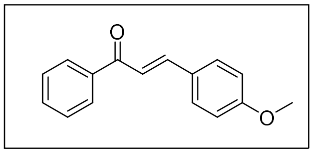

Yellowish solid solid solid (yield 82%). ^{1}H NMR (400 MHz, $CDCl_3$) δ 8.01 (dd, *J* = 8.3, 1.3 Hz, 2H), 7.79 (d, *J* = 15.6 Hz, 1H), 7.63 – 7.55 (m, 3H), 7.49 (t, *J* = 7.4 Hz, 2H), 7.42 (d, *J* = 15.6 Hz, 1H), 6.93 (d, *J* = 8.8 Hz, 2H), 3.85 (s, 3H). ^{13}C NMR (101 MHz, $CDCl_3$) δ 190.6, 161.7, 144.7, 138.5, 132.6, 130.28, 128.6, 128.4, 127.6, 119.7, 114.4, 55.5.

(E)-3-(4-fluorophenyl)-1-phenylprop-2-en-1-one[5] *(1d).*

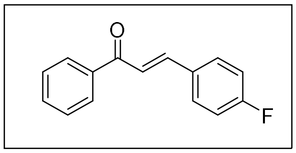

White solid (yield 85%). ^{1}H NMR (400 MHz, $CDCl_3$) δ 8.03 – 8.01 (m, 2H), 7.78 (d, *J* = 15.7 Hz, 1H), 7.69 – 7.55 (m, 3H), 7.54 – 7.41 (m, 3H), 7.12 (t, *J* = 8.6 Hz, 2H). ^{13}C NMR (101 MHz, $CDCl_3$) δ 190.35, 164.07 (d, J = 251.9 Hz), 143.56, 138.11, 132.90, 131.13 (d, J = 3.4 Hz), 130.38 (d, J = 8.5 Hz), 128.69, 128.50, 121.74 (d, J = 2.3 Hz), 116.17 (d, J = 21.9 Hz).

(E)-3-(3-fluorophenyl)-1-phenylprop-2-en-1-one (1e).

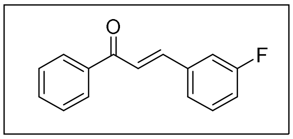

White solid (yield 80%). ^{1}H NMR (400 MHz, Chloroform-*d*) δ 8.06 – 8.00 (m, 2H), 7.76 (d, *J* = 15.7 Hz, 1H), 7.64 – 7.57 (m, 1H), 7.57 – 7.47 (m, 3H), 7.44 – 7.32 (m, 3H), 7.12 (t, *J* = 8.9 Hz, 1H). ^{13}C NMR (101 MHz, Chloroform-*d*) δ 190.19, 163.05 (d, *J* = 246.9 Hz), 143.32 (d, *J* = 2.7 Hz), 137.92,

137.13 (d, *J* = 7.6 Hz), 133.05, 130.54 (d, *J* = 8.3 Hz), 128.73, 128.55, 124.60 (d, *J* = 2.8 Hz), 123.16, 117.41 (d, *J* = 21.5 Hz), 114.49 (d, *J* = 21.9 Hz).

(E)-1-phenyl-3-(4-(trifluoromethyl)phenyl)prop-2-en-1-one[6] (1f).

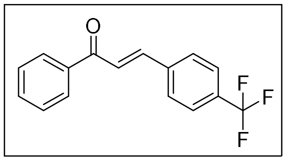

White solid (yield 81%). ^{1}H NMR (400 MHz, $CDCl_3$) δ 8.04 (d, *J* = 7.5 Hz, 2H), 7.81 (d, *J* = 15.7 Hz, 1H), 7.77 – 7.65 (m, 4H), 7.62 – 7.58 (m, 2H), 7.54 – 7.51 (m, 2H). ^{13}C NMR (101 MHz, $CDCl_3$) δ 190.1, 142.8, 138.3, 137.8, 133.2, 131.9 (q, *J* = 32.3 Hz), 128.8, 128.6, 128.5, 125.9 (q, *J* = 4 Hz), 124.2, 123.8(q, *J* = 272 Hz).

(E)-1-phenyl-3-(3-(trifluoromethyl)phenyl)prop-2-en-1-one[7] (1g).

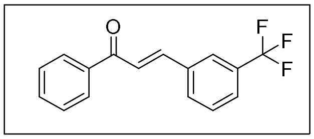

White solid (yield 78%). ^{1}H NMR (400 MHz, Chloroform-*d*) δ 8.07 – 8.02 (m, 2H), 7.89 (s, 1H), 7.85 – 7.79 (m, 2H), 7.69 – 7.50 (m, 6H). ^{13}C NMR (101 MHz, $CDCl_3$) δ 190.05, 142.87, 137.79, 135.66, 133.16, 131.68, 131.50 (q, *J* = 32.3 Hz), 129.56, 128.77, 128.59, 126.87 (q, *J* = 4 Hz), 124.70 (q, *J* = 4 Hz), 123.82 (q, *J* = 273.7 Hz), 123.61.

(E)-3-(3,4-difluorophenyl)-1-phenylprop-2-en-1-one (1h).

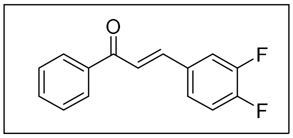

White solid (yield 78%). ^{1}H NMR (400 MHz, Chloroform-*d*) δ 8.04 – 7.99 (m, 2H), 7.71 (d, *J* = 15.7 Hz, 1H), 7.63 – 7.58 (m, 1H), 7.55 – 7.42 (m, 4H), 7.40 – 7.34 (m, 1H), 7.21 (dt, *J* = 9.7, 8.3 Hz, 1H). ^{13}C NMR (101 MHz, Chloroform-*d*) δ 189.94, 151.64 (dd, *J* = 254,5 13.1 Hz), 150.66 (dd, *J* = 250.5, 13.1 Hz), 142.36 (t, *J* = 2.0 Hz), 137.84, 133.11, 132.13 (dd, *J* = 5.9, 4.0 Hz), 128.75, 128.53, 125.36 (dd, *J* = 6.6, 3.4 Hz), 122.83 (d, *J* = 2.4 Hz), 117.94 (d, *J* = 17.7 Hz), 116.51 (d, *J* = 17.6 Hz).

(E)-1-phenyl-3-(thiophen-2-yl)prop-2-en-1-one[8] (1j).

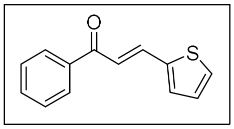

Orange solid (yield 68%). ^{1}H NMR (400 MHz, $CDCl_3$) δ 8.01 (dd, *J* = 8.3, 1.3 Hz, 2H), 7.95 (d, *J* = 15.3 Hz, 1H), 7.61 – 7.55 (m, 1H), 7.53 – 7.47 (m, 2H), 7.43 (d, *J* = 5.0 Hz, 1H), 7.37 (d, *J* = 5.0 Hz, 2H), 7.10 (dd, *J* = 5.0, 3.6 Hz, 1H). ^{13}C NMR (101 MHz, $CDCl_3$) δ 189.9, 140.4, 138.1, 137.3, 132.8, 132.2, 128.9, 128.7, 128.43, 128.41, 120.7.

(E)-1-phenyl-3-(thiophen-2-yl)prop-2-en-1-one[8] (1k).

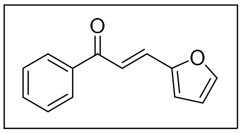

Orange oil (yield 62%). ^{1}H NMR (400 MHz, $CDCl_3$) δ 8.06 – 8.00 (m, 2H), 7.64 – 7.43 (m, 6H), 6.72 (d, *J* = 3.4 Hz, 1H), 6.52 (dd, *J* = 3.4, 1.8 Hz, 1H). ^{13}C NMR (101 MHz, $CDCl_3$) δ 189.9, 151.7, 145.0, 138.2, 132.9, 130.8, 128.7, 128.5, 119.3, 116.4, 112.8.

(E)-4-phenylbut-3-en-2-one[9] (1l)

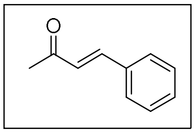

Yellow solid (yield 74%). ^{1}H NMR (400 MHz, Chloroform-*d*) δ 7.57 – 7.49 (m, 3H), 7.43 – 7.38 (m, 3H), 6.72 (d, *J* = 16.3 Hz, 1H), 2.39 (s, 3H). ^{13}C NMR (101 MHz, $CDCl_3$) δ 198.54, 143.52, 134.39, 130.57, 129.00, 128.28, 127.14, 27.56.

(E)-3-(4-chlorophenyl)-1-phenylprop-2-en-1-one (1m)

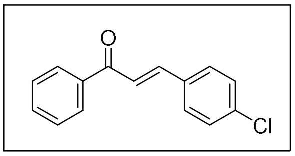

Pale yellow solid (69% yield). ^{1}H NMR (400 MHz, Chloroform-d) δ 8.05 – 7.99 (m, 1H), 7.76 (d, J = 15.7 Hz, 1H), 7.63 – 7.56 (m, 2H), 7.55 – 7.49 (m, 2H), 7.42 – 7.38 (m, 1H). ^{13}C NMR (101 MHz, $CDCl_3$) δ 190.41, 143.49, 138.14, 136.58, 133.49, 133.10, 129.75, 129.40, 128.83, 128.65, 122.56.

(E)-1-(4-bromophenyl)-3-phenylprop-2-en-1-one (1n)

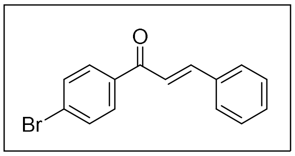

Pale yellow solid (64% yield). ^{1}H NMR (400 MHz, Chloroform-d) δ 7.93 – 7.87 (m, 1H), 7.82 (d, J = 15.7 Hz, 1H), 7.70 – 7.61 (m, 2H), 7.48 (d, J = 15.7 Hz, 1H), 7.45 – 7.41 (m, 2H). ^{13}C NMR (101 MHz, $CDCl_3$) δ 189.57, 145.59, 137.03, 134.79, 132.08, 130.93, 130.18, 129.16, 128.67, 128.06, 121.57.

(E)-3-(4-iodophenyl)-1-phenylprop-2-en-1-one (1o)

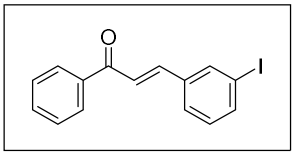

Pale yellow solid (47%). ^{1}H NMR (400 MHz, Chloroform-d) δ 8.05 – 7.98 (m, 1H), 7.77 – 7.66 (m, 1H), 7.64 – 7.56 (m, 1H), 7.55 – 7.48 (m, 1H), 7.16 (t, J = 7.8 Hz, 0H). ^{13}C NMR (101 MHz, $CDCl_3$) δ 190.24, 143.06, 139.33, 138.01, 137.19, 136.94, 133.18, 130.73, 128.85, 128.69, 127.98, 123.23, 94.93.

(E)-4-(3-oxo-3-phenylprop-1-en-1-yl)benzonitrile (1p)

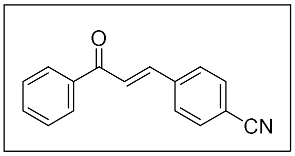

Pale yellow solid (53% yield). ^{1}H NMR (400 MHz, Chloroform-d) δ 8.06 – 8.01 (m, 1H), 7.78 (d, J = 15.7 Hz, 0H), 7.76 – 7.70 (m, 2H), 7.65 – 7.58 (m, 1H), 7.53 (ddt, J = 8.2, 6.6, 1.2 Hz, 1H). ^{13}C NMR (101 MHz, $CDCl_3$) δ 189.91, 142.68, 142.24, 139.32, 137.72, 133.47, 132.85, 128.95, 128.85, 128.72, 125.16, 118.56, 113.63, 109.59.

methyl (E)-4-(3-oxo-3-phenylprop-1-en-1-yl)benzoate (1q)

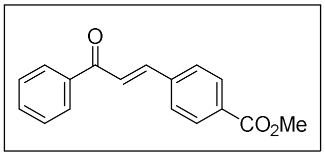

White solid (43% yield). ^{1}H NMR (400 MHz, Chloroform-d) δ 8.11 – 8.06 (m, 1H), 8.06 – 8.01 (m, 1H), 7.82 (d, J = 15.8 Hz, 1H), 7.74 – 7.68 (m, 1H), 7.65 – 7.58 (m, 1H), 7.56 – 7.48 (m, 1H), 3.95 (s, 2H). ^{13}C NMR (101 MHz, $CDCl_3$) δ 120.32, 96.61, 73.38, 69.24, 67.99, 63.24, 61.65, 60.30, 58.88, 58.71, 58.40, 54.23.

(E)-1-phenyl-3-(pyridin-3-yl)prop-2-en-1-one[10] (1r)

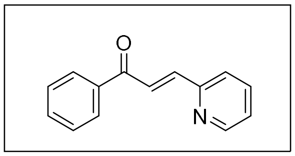

Pale yellow solid (yield 76%). ^{1}H NMR (400 MHz, Chloroform-d) δ 8.71 – 8.66 (m, 1H), 8.17 – 8.06 (m, 3H), 7.81 – 7.71 (m, 2H), 7.63 – 7.55 (m, 1H), 7.55 – 7.46 (m, 3H), 7.30 (ddd, J = 7.7, 4.8, 1.2 Hz, 1H). ^{13}C NMR (101 MHz, $CDCl_3$) δ 190.55, 153.22, 150.21, 142.79, 137.89, 137.11, 133.22, 128.86, 128.77, 125.69, 125.59, 124.58.

General method for γ,γ-dichloroketones (2a-r) preparation

A glass tube with rubber-lined cap was evacuated and filled with argon three times. To this tube triethanolamine (74.6 mg, 66 µL, 0.5 mmol), corresponding chalcone (50 µmol), K-PHI (5 mg) and chloroform (2 mL) were added. Resulting mixture was stirred at 50°C under irradiation od Blue LED (λ=461nm) for 20 hours. Then reaction mixture was cooled to room temperature and centrifuged, clear solution was separated and solid residue was washed with chloroform (2 mL) and centrifuged again. Organic solutions were combined and evaporated to dryness. Residue after evaporation was purified by silica gel column chromatography using mixture of hexane/diethyl ether (98:2) as an eluent.

4,4-dichloro-1,3-diphenylbutan-1-one (2a)

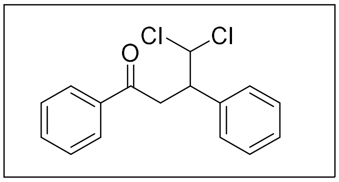

Yellow oil (yield 88%). ^{1}H NMR (400 MHz, Acetonitrile-d_3) δ 8.00 – 7.95 (m, 2H), 7.64 – 7.59 (m, 1H), 7.53 – 7.47 (m, 2H), 7.43 – 7.38 (m, 2H), 7.36 – 7.27 (m, 3H), 6.28 (d, *J* = 4.9 Hz, 1H), 4.10 (dt, *J* = 9.3, 4.9 Hz, 1H), 3.87 – 3.67 (m, 2H). ^{13}C NMR (101 MHz, CD_3CN) δ 197.2, 138.4, 136.7, 133.4, 129.3, 128.7, 128.3, 128.0, 127.8, 77.1, 50.8, 39.4. Elemental analysis calculated for $C_{16}H_{14}ClO_2$ Cl 24.18 found 24.77±0.01. GC-MS: m/z 221.2 [M-2HCl]

4,4-dichloro-1,3-diphenylbutan-1-one (2a-d$_1$)

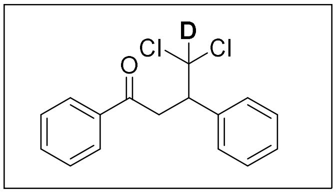

Yellow oil. ^{1}H NMR (400 MHz, Acetonitrile-d_3) δ 8.01 – 7.93 (m, 2H), 7.65 – 7.59 (m, 1H), 7.53 – 7.47 (m, 2H), 7.43 – 7.39 (m, 2H), 7.36 – 7.26 (m, 3H), 4.09 (dd, *J* = 8.7, 4.9 Hz, 1H), 3.87 – 3.66 (m, 2H). zzi^{13}C NMR (101 MHz, CD_3CN) δ 198.10, 139.25, 137.58, 134.31, 130.18, 129.61, 129.14, 128.90, 128.65, 78.01-77.46 (t, *J* = 27.866 Hz), 51.54, 40.31.

4,4-dichloro-1-phenyl-3-(p-tolyl)butan-1-one (2b)

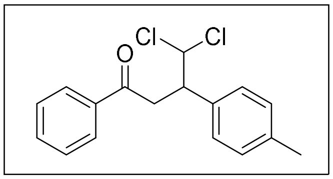

Yellow oil (yield 89%). ^{1}H NMR (400 MHz, Acetonitrile-d_3) δ 7.99 – 7.94 (m, 2H), 7.64 – 7.59 (m, 1H), 7.52 – 7.47 (m, 2H), 7.30 – 7.26 (m, 2H), 7.14 (d, *J* = 7.9 Hz, 2H), 6.25 (d, *J* = 4.8 Hz, 1H), 4.05 (dt, *J* = 9.2, 4.8 Hz, 1H), 3.85 – 3.63 (m, 2H), 2.29 (s, 3H). ^{13}C NMR (101 MHz, CD_3CN) δ 197.3, 137.6, 136.7, 135.3, 133.4, 129.2, 128.9, 128.7, 128.0, 77.3, 50.4, 39.4, 20.1. GC-MS: m/z 234.1 [M-2HCl]

4,4-dichloro-3-(4-methoxyphenyl)-1-phenylbutan-1-one (2c)

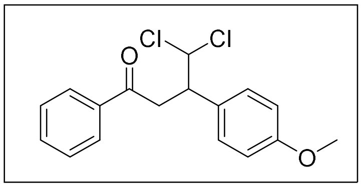

Yellow oil (yield 83%). ^{1}H NMR (400 MHz, Acetonitrile-d_3) δ 7.99 (d, *J* = 7.2 Hz, 2H), 7.68 – 7.62 (m, 1H), 7.53 (t, *J* = 7.7 Hz, 2H), 7.38 – 7.32 (m, 2H), 6.93 – 6.86 (m, 2H), 6.26 (d, *J* = 4.7 Hz, 1H), 4.07 (dt, *J* = 9.1, 4.8 Hz, 1H), 3.84 – 3.76 (m, 4H), 3.70 (dd, *J* = 17.8, 5.0 Hz, 1H). ^{13}C NMR (101 MHz, CD_3CN) δ 197.3, 159.2, 136.7, 133.4, 130.4, 130.1, 128.7, 128.0, 113.5, 77.5, 54.9, 50.0, 39.4. GC-MS: m/z 250.1 [M-2HCl]

4,4-dichloro-3-(4-fluorophenyl)-1-phenylbutan-1-one (2d)

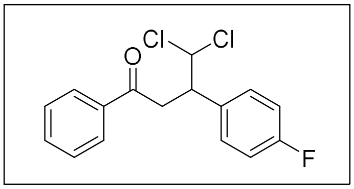

Yellow oil (yield 84%). ^{1}H NMR (400 MHz, Acetonitrile-d_3) δ 8.00 (dd, *J* = 8.4, 1.3 Hz, 2H), 7.68 – 7.62 (m, 1H), 7.56 – 7.50 (m, 2H), 7.49 – 7.44 (m, 2H), 7.14 – 7.07 (m, 2H), 6.29 (d, *J* = 4.7 Hz, 1H), 4.13 (dt, *J* = 8.4, 5.0 Hz, 1H), 3.85 – 3.70 (m, 2H). ^{13}C NMR (101 MHz, Acetonitrile-d_3) δ 197.1, 162.2 (d, *J* = 244.2 Hz), 136.6, 134.3 (d, *J* = 3.3 Hz), 133.5, 131.2 (d, *J* = 8.2 Hz), 128.7, 128.0, 114.9 (d, *J* = 21.5 Hz), 76.9, 49.9, 39.7. ^{19}F NMR (376 MHz, Acetonitrile-d_3) δ -116.45 (tt, *J* = 8.9, 5.4 Hz). GC-MS: m/z 238.1 [M-2HCl]

4,4-dichloro-3-(3-fluorophenyl)-1-phenylbutan-1-one (2e)

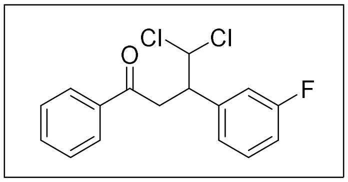

Yellow oil (yield 80%). ^{1}H NMR (400 MHz, Acetonitrile-d_3) δ 8.01 (dt, *J* = 8.5, 1.6 Hz, 2H), 7.68 – 7.63 (m, 1H), 7.56 – 7.51 (m, 2H), 7.38 (td, *J* = 8.0, 6.2 Hz, 1H), 7.28 (d, *J* = 7.8 Hz, 1H), 7.23 (dt, *J* = 10.5, 2.1 Hz, 1H), 7.11 – 7.03 (m, 1H), 6.31 (d, *J* = 4.8 Hz, 1H), 4.15 (dt, *J* = 8.5, 5.0 Hz, 1H), 3.88 – 3.72 (m, 2H). ^{13}C NMR (101 MHz, Acetonitrile-d_3) δ 196.9, 162.4 (d, *J* = 243.4 Hz), 141.1 (d, *J* = 7.4 Hz), 136.6, 133.5, 130.0 (d, *J* = 8.3 Hz), 128.7, 128.0, 125.5 (d, *J* = 2.8 Hz), 116.1 (d, *J* = 22.2 Hz),

114.5 (d, J = 21.1 Hz), 76.5, 50.3, 39.5. ^{19}F NMR (376 MHz, Acetonitrile-d_3) δ -114.90 – 115.03 (m). GC-MS: m/z 238.1 [M-2HCl]

4,4-dichloro-1-phenyl-3-(4-(trifluoromethyl)phenyl)butan-1-one ((2f)

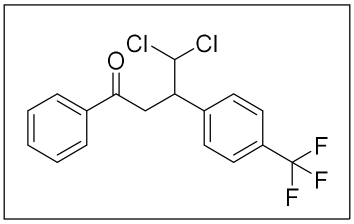

Yellow oil (yield 85%). ^{1}H NMR (400 MHz, Acetonitrile-d_3) δ 8.00 – 7.95 (m, 2H), 7.68 – 7.59 (m, 5H), 7.53 – 7.47 (m, 2H), 6.32 (d, J = 4.8 Hz, 1H), 4.20 (dt, J = 8.9, 4.9 Hz, 1H), 3.89 – 3.73 (m, 2H). ^{13}C NMR (101 MHz, CD_3CN) δ 196.8, 142.9, 136.5, 133.5, 130.2, 129.1 (q, J = 37 Hz), 128.7, 128.0, 125.0 (q, J = 4 Hz), 124.3 (q, J = 37 Hz), 76.3, 50.4, 39.6. ^{19}F NMR (376 MHz, Acetonitrile-d_3) δ - 114.90 – 115.03 (m). GC-MS: m/z 288.1 [M-2HCl]

4,4-dichloro-1-phenyl-3-(3-(trifluoromethyl)phenyl)butan-1-one (2g)

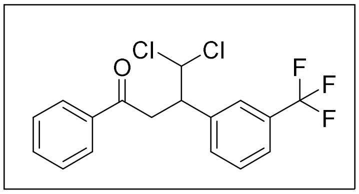

Yellow oil (yield 82%). ^{1}H NMR (400 MHz, Acetonitrile-d_3) δ 8.02 – 7.94 (m, 2H), 7.77 (s, 1H), 7.70 (d, J = 7.7 Hz, 1H), 7.65 – 7.60 (m, 2H), 7.56 – 7.47 (m, 3H), 6.32 (d, J = 4.8 Hz, 1H), 4.20 (dt, J = 8.3, 5.0 Hz, 1H), 3.90 – 3.72 (m, 2H). ^{13}C NMR (101 MHz, CD_3CN) δ 196.89, 139.58, 136.49, 133.53, 133.40, 129.72 (q, J = 32.3 Hz), 129.03, 128.72, 128.39, 128.05, 126.20 (q, J = 4 Hz), 124.53 (q, J = 4 Hz), 124.34 (q, J = 273 Hz), 76.38, 50.35, 39.59. ^{19}F NMR (376 MHz, cd_3cn) δ -63.03. GC-MS: m/z 288.1 [M-2HCl]

4,4-dichloro-3-(3,4-difluorophenyl)-1-phenylbutan-1-one (2h)

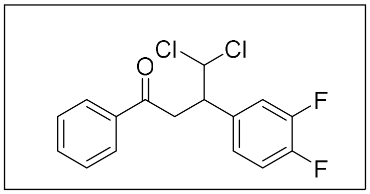

Yellow oil (yield 70%). ^{1}H NMR (400 MHz, Acetonitrile-d_3) δ 8.02 – 7.94 (m, 2H), 7.65 – 7.60 (m, 1H), 7.53 – 7.48 (m, 2H), 7.42 – 7.34 (m, 1H), 7.26 – 7.21 (m, 2H), 6.26 (d, *J* = 4.7 Hz, 1H), 4.10 (dt, *J* = 7.8, 5.2 Hz, 1H), 3.76 – 3.71 (m, 2H). ^{13}C NMR (101 MHz, CD_3CN) δ 196.8, 150.8 (m), 148.4 (m), 136.5, 135.7 (m), 128.7, 128.0, 126.2 (m), 118.4 (d, *J* = 18.2 Hz), 116.9, 118.4 (d, *J* = 17.2 Hz), 76.4, 49.7, 39.7. ^{19}F NMR (376 MHz, Acetonitrile-d_3) δ -140.02 – 140.20 (m), -141.30 – 141.47 (m). GC-MS: m/z 256.1 [M-2HCl]

4,4-dichloro-1-phenyl-3-(thiophen-2-yl)butan-1-one (2i)

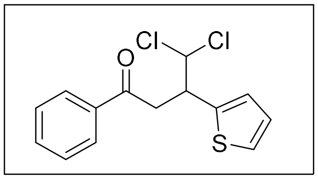

Yellow oil (yield 54%). ^{1}H NMR (400 MHz, Acetonitrile-d_3) δ 8.01 – 7.96 (m, 2H), 7.66 – 7.60 (m, 1H), 7.54 – 7.48 (m, 2H), 7.32 (dd, *J* = 5.1, 1.2 Hz, 1H), 7.13 – 7.09 (m, 1H), 6.99 (dd, *J* = 5.1, 3.6 Hz, 1H), 6.31 (d, *J* = 3.7 Hz, 1H), 4.42 (dt, *J* = 8.4, 4.2 Hz, 1H), 3.84 – 3.63 (m, 2H). ^{13}C NMR (101 MHz, CD_3CN) δ 196.7, 140.4, 136.5, 133.6, 128.8, 128.1, 127.5, 126.7, 125.4, 76.6, 46.1, 41.0.

4,4-dichloro-3-(furan-2-yl)-1-phenylbutan-1-one (2j)

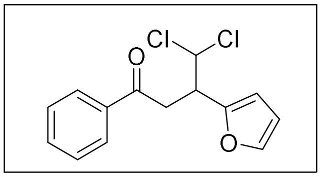

Yellow oil (yield 72%). ^{1}H NMR (400 MHz, Acetonitrile-d_3) δ 8.01 (d, *J* = 7.2 Hz, 2H), 7.67 – 7.62 (m, 1H), 7.52 (t, *J* = 7.7 Hz, 2H), 7.45 – 7.42 (m, 1H), 6.37 (dd, *J* = 3.2, 1.8 Hz, 1H), 6.33 (d, *J* = 3.2 Hz, 1H), 6.30 (d, *J* = 3.8 Hz, 1H), 4.28 – 4.19 (m, 1H), 3.78 (dd, *J* = 18.0, 8.8 Hz, 1H), 3.61 (dd, *J* = 18.0, 4.3 Hz, 1H). ^{13}C NMR (101 MHz, CD_3CN) δ 196.8, 151.9, 142.3, 136.5, 133.5, 128.8, 128.1, 110.6, 108.4, 75.2, 44.8, 37.8.

5,5-dichloro-4-phenylpentan-2-one (2k)

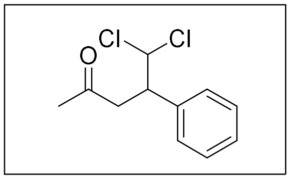

Yellow oil (yield 54%).^{1}H NMR (400 MHz, Acetonitrile-d_3) δ 7.39 – 7.26 (m, 5H), 6.16 (d, *J* = 4.8 Hz, 1H), 3.93 – 3.81 (m, 1H), 3.19 (dd, *J* = 6.8, 2.8 Hz, 2H), 2.07 (s, 3H). ^{13}C NMR (101 MHz, CD_3CN) δ 206.1, 138.2, 129.2, 128.3, 127.8, 77.0, 50.3, 43.8, 29.6. GC-MS: m/z 194.1 [M-HCl]

3-(dichloromethyl)cyclohexan-1-one (2l)

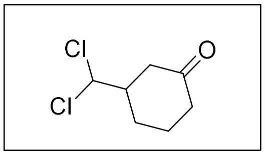

Yellow oil (yield 78%). ^{1}H NMR (400 MHz, Chloroform-*d*) δ 5.79 (d, *J* = 2.9 Hz, 1H), 2.70 – 2.62 (m, 1H), 2.50 – 2.38 (m, 3H), 2.32 (td, *J* = 14.2, 13.6, 6.3 Hz, 1H), 2.22 – 2.10 (m, 2H), 1.76 – 1.64 (m, 2H). ^{13}C NMR (101 MHz, $CDCl_3$) δ 209.2, 76.4, 48.5, 42.8, 40.9, 26.8, 23.9. GC-MS: m/z 180.0

4,4-dichloro-3-(4-chlorophenyl)-1-phenylbutan-1-one (2m)

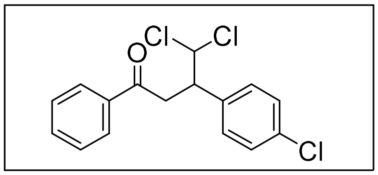

Yellow oil. ^{1}H NMR (400 MHz, Chloroform-d) δ 8.00 – 7.94 (m, 1H), 7.62 – 7.57 (m, 1H), 7.51 – 7.44 (m, 1H), 7.31 (s, 2H), 6.08 (d, J = 4.0 Hz, 0H), 4.13 (ddd, J = 8.0, 5.4, 4.0 Hz, 1H), 3.80 – 3.63 (m, 1H). ^{13}C NMR (101 MHz, $CDCl_3$) δ 196.78, 136.48, 134.06, 133.77, 133.50, 130.56, 129.04, 128.91, 128.82, 128.73, 128.20. GC-MS: m/z 254.1 [M-2HCl]

1-(4-bromophenyl)-4,4-dichloro-3-phenylbutan-1-one (2n)

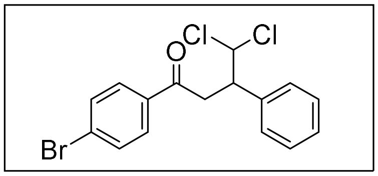

Yellow oil. ^{1}H NMR (400 MHz, Chloroform-d) δ 7.86 – 7.80 (m, 1H), 7.64 – 7.59 (m, 1H), 7.38 – 7.29 (m, 2H), 6.09 (d, J = 4.1 Hz, 0H), 4.14 (ddd, J = 7.7, 5.5, 4.1 Hz, 1H), 3.76 – 3.63 (m, 1H). ^{13}C NMR

(101 MHz, $CDCl_3$) δ 196.10, 137.95, 135.38, 132.31, 132.22, 132.17, 130.75, 130.24, 130.15, 129.73, 129.05, 129.00, 128.85, 128.70, 128.42, 128.21. GC-MS: m/z 298 [M-2HCl]

4,4-dichloro-3-(3-iodophenyl)-1-phenylbutan-1-one (2o)

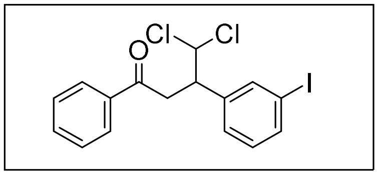

Yellow oil. ^{1}H NMR (400 MHz, Chloroform-d) δ 8.03 – 7.93 (m, 1H), 7.73 (t, J = 1.8 Hz, 1H), 7.66 – 7.57 (m, 1H), 7.52 – 7.46 (m, 1H), 7.36 (dt, J = 7.7, 1.4 Hz, 1H), 7.07 (t, J = 7.8 Hz, 1H), 6.08 (d, J = 4.0 Hz, 1H), 4.09 (ddd, J = 7.8, 5.3, 4.0 Hz, 1H), 3.82 – 3.61 (m, 1H). ^{13}C NMR (101 MHz, $CDCl_3$) δ 196.67, 140.39, 138.08, 137.24, 136.48, 133.78, 130.27, 128.91, 128.58, 128.23, 94.49. GC-MS: m/z 346.0 [M-2HCl]

Preparation of *dichloro-ketone* derived compound (3-6a)

4-oxo-2,4-diphenylbutanal (3a)

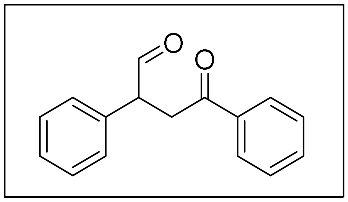

In a sealed glass tube, it was added 4,4-dichloro-1,3-diphenylbutan-1-one (2a) (0.05 mmol, 14.66 mg), KOH (100 mg), EtOH (3 mL). The reaction vessel has then been kept to react for 1 hour in an oil bath at 100 °C. After, the reaction was left to cool down to room temperature. Afterwards, ice was added to the reaction mixture before adding concentrated HCl. The mixture was then neutralized with $NaHCO_3$ and extracted with EtOAc and washed with brine. Collected organic solutions were evaporated, dissolved in CH_2Cl_2 and dried over Na_2SO_4. At this stage, the yield of 3a was recorded measuring ^{1}H NMR with 1,3,5-trimethozybenzene as internal standard. The crude obtained has been purified with a chromatographic colum Hexane/EtOAc (9:1).

Yellow oil (yield 60%). ^{1}H NMR (400 MHz, Chloroform-d) δ 9.81 (d, J = 0.7 Hz, 1H), 7.98 (dd, J = 8.4, 1.4 Hz, 2H), 7.59 – 7.54 (m, 1H), 7.48 – 7.43 (m, 2H), 7.42 – 7.37 (m, 2H), 7.35 – 7.31 (m, 1H), 7.30 – 7.27 (m, 2H), 4.47 (dd, J = 8.5, 4.9 Hz, 1H), 3.96 (dd, J = 18.0, 8.5 Hz, 1H), 3.23 (ddd, J = 18.1, 4.8, 0.6 Hz, 1H). ^{13}C NMR (101 MHz, $CDCl_3$) δ 199.07, 197.34, 136.46, 135.47, 133.39, 129.34, 129.15, 128.67, 128.17, 127.97, 127.23, 53.69, 39.49.

2,4-diphenylfuran (4a)

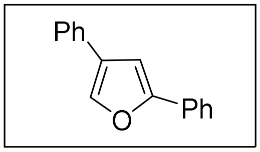

For the preparation of this copound it was followed a slightly different synthesis already report in another work[11]. In a glass tube with a Teflon-lined snap cap, it was added 4-oxo-2,4-diphenylbutanal (3a) (0.1 mmol, 23.8 mg), EtOH (1 mL), HCl 1 M (0.1 mL). The vessel has then been place in the microwave reactor at 150 °C for 15 minutes. The reaction mixture was diluted with EtOAc and then washed with a sature solution of $NaHCO_3$ and then with Brine. Collected organic solutions were evaporated, dissolved in CH_2Cl_2 and dried over Na_2SO_4. At this stage, the yield of 4a was recorded measuring ^{1}H NMR with 1,3,5-trimethozybenzene as internal standard. The crude obtained has been purified with a chromatographic colum Hexane/EtOAc (99:1).

Colorless crystals (yield: 70%). ^{1}H NMR (400 MHz, Chloroform-d) δ 7.77 (d, J = 0.9 Hz, 1H), 7.76 – 7.71 (m, 2H), 7.58 – 7.53 (m, 2H), 7.45 – 7.39 (m, 4H), 7.34 – 7.27 (m, 2H), 6.98 (d, J = 0.9 Hz, 1H). ^{13}C NMR (101 MHz, $CDCl_3$) δ 154.98, 138.03, 132.49, 130.78, 128.98, 128.86, 128.49, 127.74, 127.27, 125.94, 123.98, 104.11.

2,4-diphenyl-1H-pyrrole (5a)

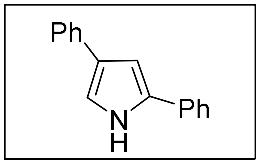

For the preparation of this compound it was followed a slightly different synthesis already report in another work[13]. In a glass tube with a Teflon-lined snap cap, it was added 4-oxo-2,4-diphenylbutanal (3a) (0.1 mmol, 23.8 mg), a mixture THF:AcOH (1:1, 1 mL), $AcONH_4$ (0.4 mmol, 31 mg). The vessel has then been place in the microwave reactor at 170 °C for 15 minutes. The reaction mixture was diluted with EtOAc and then washed with a sature solution of $NaHCO_3$ and then with Brine. Collected organic solutions were evaporated, dissolved in CH_2Cl_2 and dried over Na_2SO_4. At this stage, the yield of 5a was recorded measuring ^{1}H NMR with 1,3,5-trimethozybenzene as internal standard. The crude obtained has been purified with a chromatographic colum Hexane/EtOAc (85:15).

White solid (yield: 89%). ^{1}H NMR (400 MHz, Chloroform-d) δ 8.46 (s, 1H), 7.61 – 7.55 (m, 2H), 7.55 – 7.50 (m, 2H), 7.38 (ddd, J = 10.4, 8.4, 7.0 Hz, 4H), 7.30 – 7.18 (m, 2H), 7.15 (dd, J = 2.7, 1.7 Hz, 1H), 6.84 (dd, J = 2.8, 1.7 Hz, 1H). ^{13}C NMR (101 MHz, $CDCl_3$) δ 135.60, 133.20, 132.58, 129.10, 128.81, 126.73, 126.64, 125.89, 125.31, 123.99, 115.68, 104.09.

1,2,4-triphenyl-1H-pyrrole (6a)

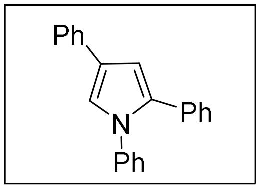

For the preparation of this compound it was followed a slightly different synthesis already report in another work[13]. In a glass tube with a Teflon-lined snap cap, it was added 4-oxo-2,4-diphenylbutanal (3a) (0.1 mmol, 23.8 mg), a mixture THF:AcOH (1:1, 1 mL), phenylamine (0.2 mmol, 18 µL). The vessel has then been place in the microwave reactor at 170 °C for 15 minutes. The reaction mixture was diluted with EtOAc and then washed with a sature solution of $NaHCO_3$ and then with Brine. Collected organic solutions were evaporated, dissolved in CH_2Cl_2 and dried over Na_2SO_4. At this stage, the yield of 6a was recorded, measuring ^{1}H NMR with 1,3,5-trimethozybenzene as internal standard. The crude obtained has been purified with a chromatographic colum Hexane/EtOAc (85:15).

White solid (yield: 100%). ^{1}H NMR (400 MHz, Chloroform-d) δ 7.66 – 7.61 (m, 2H), 7.45 – 7.30 (m, 5H), 7.30 – 7.18 (m, 9H), 6.78 (d, J = 1.8 Hz, 1H). ^{13}C NMR (101 MHz, $CDCl_3$) δ 140.42, 135.26, 134.88, 132.80, 129.20, 128.84, 128.43, 128.26, 126.93, 126.68, 125.98, 125.79, 125.65, 125.24, 121.01, 108.86.

General method for imines preparations (2'a-f)

A glass tube with rubber-lined cap was evacuated and filled with argon three times. To this tube, corresponding amine (0.1 mmol for 1'a-b, 0.2 mmol for 1'c-f), K-PHI (5 mg) and chloroform (2 mL) were added. Resultig mixture was stirred at 50°C under irradiation od Blue LED (λ = 461nm) for 20 hours. Then reaction mixture was cooled to room temperature and centrifuged, clear solution was separated and solid residue was washed with chloroform (2 mL) and centrifuged again. Organic solutions were combined and evaporated to dryness (Supplementary Figure 92, Supplementary Figure 93).

Supplementary Tables

Supplementary Table 1. Screening reactions[a,b]

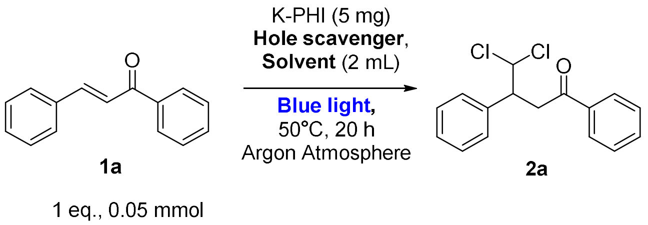

Entry	Hole Scavenger	Solvent	Yield, %
1	THIQ (1 eq, 0.05 mmol)	$CHCl_3$	17
2	THIQ (2 eq, 0.1 mmol)	$CHCl_3$	33
3	THIQ (3 eq, 0.15 mmol)	$CHCl_3$	41
4	THIQ-d_2 (3 eq, 0.15 mmol)	$CHCl_3$	36
5	THIQ (4 eq, 0.2 mmol)	$CHCl_3$	51
6	TEOA (3 eq., 0.15 mmol)	$CHCl_3$	62
7	TEOA (10 eq., 0.5 mmol)	$CHCl_3$	97
8[b]	TEOA (10 eq., 0.5 mmol)	$CHCl_3$	96
9	TEA (10 eq., 0.5 mmol)	$CHCl_3$	37
10	DiPEA (10 eq., 0.5 mmol)	$CHCl_3$	+
11	MeOH (10 eq., 0.5 mmol)	$CHCl_3$	10
12	EtOH (10 eq., 0.5 mmol)	$CHCl_3$	7
13	iPrOH (10 eq., 0.5 mmol)	$CHCl_3$	6
14	BnOH (10 eq., 0.5 mmol)	$CHCl_3$	8
15	TEOA (10 eq., 0.5 mmol)	DMF	-
16	TEOA (10 eq., 0.5 mmol)	Triethyl orthoformate	-
17	TEOA (10 eq., 0.5 mmol)	CH_2Cl_2	+
18[c]	TEOA (10 eq., 0.5 mmol)	CH_2Cl_2	-

19	TEOA (10 eq., 0.5 mmol)	CH_2Cl_2	-
20	TEOA (10 eq., 0.5 mmol)	$C_2H_2Cl_4$	-
21[d]	TEOA (10 eq., 1 mmol)	$CHCl_3$	64
22	TEOA (10 eq., 0.5 mmol)	CH_2Cl_2	-
23	TEOA (10 eq., 0.5 mmol)	$CHCl_3$/DMSO 1:1	47

a) Reaction conditions: 1 eq., 0.05 mmol, 10.4 mg of **1a**; under light irradiation (λ = 461 nm, 51±0.03 mW cm^{-2}, blue LED; b) Yelds were recorded by GC-MS. When the chromatogram was not possible to estimate yields in this way, GC-MS was used only as qualitative analysis with the following reported signs in the table: +) product formation is observed at the end of reaction; -): product formation is not observed at the end of reaction; b) reaction performed under air; c) 100mg of CHI_3 were dissolved in the solvent; d) reaction performed with 'coold finger' at 20 °C, 1 eq., 0.1 mmol, 20.8 mg of **1a**, 4 mL of solvent.

Supplementary Table 2. Catalyst recycling.

Entry	K-PHI refill	Yield (%)[a]
1	-	97
2	+1.5 mg	96
3	2.5 mg	97

a) Yields were calculated by GC-MS. After following the general procedure for the preparation of dichloroketones (section 5), the residual catalyst was washed with water (2 mL) and centrifuged for two times. The residue was then place overnight in the vacuum oven at 50°C. Before reusal, the lost mass has been refilled with fresh catalyst.

Supplementary Table 3. Scale-up experiment.[a]

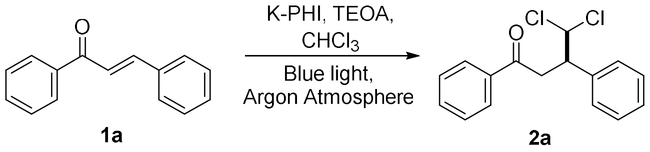

Entry	1a, mmol (mg)	K-PHI, mg	TEOA, mL	$CHCl_3$, mL	Scale factor[b]	Time, h	Yield of 2a,[c] %
1	0.05 (10.4)	5	0.066	2	1	20	88
2	0.25 (52)	25	0.335	10	5	20	43
3	0.25 (52)	25	0.335	10	5	40	71
4	0.50 (104)	50	0.67	20	10	20	38
5	5.00 (1040)	250	6.6	100	100	20	23

[a] Reaction conditions: blue LED (λ = 461 nm, 51±0.03 mW cm^{-2}); [b] with respect to enone **1a**; [c] determined by GC-MS

Supplementary Table 4. Screening reaction with chloroform alone.

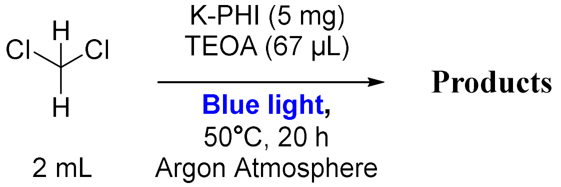

Entry	K-PHI	TEOA	Light	Major products[a]
1	-	+	+	-
2	+	+	+	$C_2H_2Cl_4$, C_2HCl_5, C_2HCl_3, C_2Cl_4
3	+	-	+	C_2Cl_6
4	-	-	+	-
5	-	+	-	-
6	+	+	-	-
7	+	-	-	-
8	-	-	-	-

a) Only a qualitative analysis by GC-MS was possible because of multiple peaks in chromatogram.

Supplementary Notes

Supplementary Note 1

We investigated the reasons why TEOA is better hole scavenger than alcohols. As an example, in Supplementary Figure 94 we report cyclic voltammograms, comparing oxidation potential of TEOA and benzyl alcohol. It is clear that TEOA has much lower oxidation potential than benzylic alcohol, +0.5 V vs +1.5 V. Therefore TEOA is oxidized faster, leading to the formation of much higher amount of K-PHI radical anion species to carry on the reaction.

Supplementary Note 2

Enones **1p-r** gave dichloroketones **2p-r** as evidenced by ^{1}H NMR spectra of the reaction mixture (Supplementary Figure 95-100). Formation of these products was supported by the presence of doublet at 6.08-6.20 ppm in the ^{1}H NMR spectrum assigned to the $CHCl_2$ group, which is similar to the synthesized dichloroketone **2a-l**

Our attempts to isolate CN-, CO_2Me- and pyridine-substituted dichloroketones **2p-r** by column chromatography were not successful.

Relatively low yields of dichloroketones **2p-r** bearing strong electron withdrawing groups (CO_2Me, CN, pyridine), we attribute to two factors. Firstly, electron withdrawing groups activate C=C bond for reduction, *i.e.* in the gas chromatograms of the reaction mixture we detected products of chalcones **1p-1r** C=C bond reduction. Similar behavior of electron deficient chalcones we observed earlier when studied their cyclodimerization.[12] In other words, addition of $CHCl_2$-group to the C=C bond competes with the reduction of C=C bond.

Secondly, higher lability of dichloroketones **2p-r** toward nucleophiles creates difficulties for these compounds isolation by column chromatography due to irreversible interaction with OH-groups of silicagel. We also found that reversed phase chromatography (C18 modified stationary phase, eluent – acetonitrile:water in different ratios) is not suitable for γ,γ-dichloroketones purification either.

It should be pointed out that even a relatively weak nucleophile such as oxygen of the carbonyl group in dichloroketones **2** capable for triggering the intramolecular cyclization followed by elminination of two molecules HCl. Therefore, in the GC-MS (electron ionization) we have not detected a signal with m/z of dichloroketones, e.g. m/z 292 for **2a**, but we always observe signal of the furans generated from the corresponding dichloroketone, e.g. m/z 220 for 2,4-diphenylfuran.

Supplementary Note 3

Supplementary Figure 101 shows a list of Michael acceptor that were used as substrates in the dichloromethylation reaction.

The reaction mixture was analyzed by GC-MS. Please note that all Michael acceptors gave quite complex mixture. Therefore, we provide only mass spectra of the dichloromethylated products that we were able to identify. Assignment of the specific structure to the chromatogram peak was made taking into account isotope distribution of the molecular ion (denoted as Exp. or E.). Theoretical isotope distribution (denoted as Theor. or T.) was calculated using ChemDraw 17.1.0.105 (19) software (see Supplementary Figures 102-103).

In the GC-MS of the reaction mixture using methylvinyl ketone as a substrate, we identified 1) tetrachloroethane – the product of two dichloromethyl radicals coupling, 2) product of triethanol amine dehydrogenation,[12] 3) residual triethanolamine and 4) a small amount of dichloromethylated product **2s**. Isotope distribution in the mass spectrum of **2s** molecular ion matches well with the calculated one (Supplementary Figure 102).

In the GC-MS of the reaction mixture using acrylonitrile as a substrate, we identified 1) tetrachloroethane – the product of two dichloromethyl radicals coupling, 2) product of triethanol amine dehydrogenation,[12] 3) residual triethanolamine and 4) a small amount of dichloromethylated product **2t**. Isotope distribution in the mass spectrum of **2t** molecular ion matches well with the calculated one (Supplementary Figure 103).

Any attempts to separate compounds **2s** and **2t** from the reaction mixture by column chromatography were not successful (Supplementary Note 4).

Other Michael acceptors did not give the desired product even in trace quantity.

Supplementary Note 4

We assume that high reactivity of the Michael acceptors **1s**-**1w** is responsible for either low or no yield of the products **2s**-**2w**. Many of compounds **1s**-**1w** are used as polymer precursors due to reactive C=C bond. Therefore, in the photocatalytic reaction that involves radical intermediate, they most probably undergo polymerization.

Another reason for low or no yield of the products **2s**-**2w** may consist in high reactivity of these compounds toward nucleophiles, e.g. hydroxyl groups of triethanolamine during synthesis, silicagel hydroxyl groups during purification. Worth mentioning that reversed phase

chromatography (C18 modified stationary phase, eluent – acetonitrile:water in different ratios) was not suitable for dichloroketones purification either.

Finally our conclusions about high reactivity of dichloromethylated compounds 2s-w are supported by absence of almost any data regarding synthesis of these compounds obtained from SciFinder and Reaxys (Supplementary Figure 104-113). To our surprise SciFinder output as of 24.10.2019 showed that only one article mentions methyl 4,4-dichlorobutanoate 2v – a product of $CHCl_2$ addition to the β-carbon atom of methacrylate 1v (Supplementary Figure 107). [Acta Chemica Scandinavica, Series B: Organic Chemistry and Biochemistry, B35(3), 175-8; 1981]. However, no NMR data is given for the title compound neither yield.

Compounds 2s-2u and 2w that we would have expected to obtain from the suggested Michael acceptors upon addition of $CHCl_2$-moiety, have not been reported earlier (Supplementary Figure 104-106, 108).

Similar results were obtained from Reaxys (Supplementary Figure 109-113)

Taking into account absence of any data regarding the dichloromethylated Michael acceptors 2s-2u and 2w, we conclude that synthesis of such compounds is not a trivial task. Despite high attractiveness for organic synthesis they have not been synthesized so far.

We need to point out that most of dichloroketones prepared in this work have not been reported either. However, success in dichloromethylation of enones bearing aromatic substituent we explain by higher stability of these molecules compared to small Michael acceptors under the reaction conditions. Due to steric hindrance and resonance stabilization of the aromatic substituents, diarylsubstituted enones are less susceptible for polymerization. Therefore, the path of $CHCl_2$-moiety addition to the C=C bond becomes dominant. Furthermore, isolation of dichloromethylated ketones is possible, but until the point when the structure becomes too electron deficient and hence susceptible for nucleophilic attack (see Supplementary Note 2).

Supplementary Note 5

Several halogenated compounds have been investigated as possible sources of $C_xHal_yH_z$ groups to introduce into enone 1a. Reaction mixtures were analyzed by GC-MS. Assignment of the specific structure to the chromatogram peak was made taking into account isotope distribution of the molecular ion (denoted as Exp. or E.). Theoretical isotope distribution (denoted as Theor. or T.) was calculated using ChemDraw 17.1.0.105 (19) (Supplementary Figure 114-115, 117-118, 120).

The experiment using dichloromethane as solvent showed traces of the product. There are also intense peak of TEOA (broad due high concentration) and starting chalcone (Supplementary Figure 114).

In case of using bromoform, in the reaction mixture we identified the following major products: 1) residual bromoform, 2) compound with a brutto formula C_2HBr_3, presumably 1,1,2-tribromoethene, 3) tetrabromoethene, 4) tetrabromoethane, 5) compound with a brutto formula C_9H_7OBr, presumably cinnamoyl bromide, 6) unreacted chalcone **1a**, 7) 2,4-diphenylfuran, 8) 4-bromo-1,3-diphenylbutan-1-one (Supplementary Figure 115).

Formation of tetrabromoethane suggests that similarly to chloroform, in the photocatalytic reactor bromoform yields dibromomethyl radical. Two of such radicals recombine and give detectable products in GC-MS, *i.e*. tetrabromoethane, 1,1,2-tribromoethene.

Formation of 4-bromo-1,3-diphenylbutan-1-one suggests that addition of dibromomethyl radical to the C=C bond of enone **1a** took place. Reduction of the intermediary dihaloketone yields 4-bromo-1,3-diphenylbutan-1-one. Furthermore, intermediary dibromoketone undergoes intramolecular cyclization, *i.e.* nucleophilic attack of the carbonyl oxygen atom at the carbon atom of $CHBr_2$-group, followed by elimination of two molecules HBr and yields 2,4-diphenylfuran (Supplementary Figure 116).

This mechanism is further supported by the fact that no triethanolamine was detected in the reaction mixture by GC-MS – it has been converted to the salt. After the photocatalytic experiment we observed formation of gum-like residue. Therefore, only soluble fraction of the reaction mixture was analyzed by GC-MS (Supplementary Figure 116).

In case of iodoform, i.e. solution in CH_2Cl_2, we observed formation of small amount of 2,4-diphenylfurane implying that tentative CHI_2-radical has been attached to the enone **1a**. Similarly to bromoform, no triethanolamine or its dehydrogenation products were detected in gas chromatogram suggesting acidification of the reaction mixture and subsequent binding triethanolamine to the insoluble salt (Supplementary Figure 117).

Analysis of the GC-MS data of the reaction mixture using 1,1,2,2-tetrachloroethane as a solvent revealed that this halogenated solvent partially underwent chemical transformation.

We identified the following products of tetrachloroethane conversion: 1) trichloroethene, 2) trichloroethane and 3) tetrachloroethene. *Cis*-isomer of chalcone **1a** was detected in the GC-MS of the reaction mixture (Supplementary Figure 118). In addition, two compounds, presumably isomers, with m/z 304 and similar retention times, *i.e.* 10.650 min and 10.996 min, were detected. Based on the analysis of the isotope distribution in the molecular ion, we assigned brutto formula $C_{17}H_{14}Cl_2O$ to these compounds. The compounds with the brutto formula $C_{17}H_{14}Cl_2O$ might be the

products of tetrachloroethane addition to the C=C bond of enone **1a** according to Supplementary Figure 119).

Elimination of two HCl molecules mediated by TEOA followed by one of the C=C bonds reduction yields the two products with brutto formula $C_{17}H_{14}Cl_2O$ that were detected in the GC-MS. Acidification of the reaction mixture was concluded based on the fact that TEOA was not detected by GC-MS. Instead it was converted to gum-like residue, similarly to the experiments with bromoform and iodoform, upon protonation with the generated HCl.

Reaction in tetrachlorometane led to isomerization of chalcone **1a**. Similarly to the experiments with bromoform, iodoform and tetrachloroethane, triethanolamine was converted to gum-like residue insoluble in tetrachloromethane (Supplementary Figure 120).

Overall, we conclude that K-PHI may be used to generate $C_xHal_yH_z$ radicals from the respective halogenated compounds and potentially used to extend the backbone of enones.

Supplementary Note 6

We performed a series of experiments in the batch reactor scaling up proportionally the amount of reagents/catalyst/solvent. The smallest amount of enone **1a** was 0.05 mmol and the largest amount – 5 mmol. The yield of dichloroketone **2a** (η) decreases gradually as the amount (n) of reagents, i.e. chalcone **1a**, increases (Supplementary Figure 121).

These results can be explained by the fact that the surface area of the liquid phase exposed to the light scales lower than the volume of the reaction mixture. Therefore, only a small fraction of the catalyst (close to the surface of the reaction) receives sufficient number of photons to mediate the reaction. The highest surface-area-to-volume ratio (A = 4.57) was for the reaction performed on 0.05 mmol scale, the lowest, A = 1.37, was for the reaction performed on 5 mmol scale. Assuming that the yield (η) of dichloroketone **2a** will approach zero in infinitely large photoreactor due to infinitely small number of photocatalyst located at the near surface layer and exposed to light, η versus A can be fitted with the linear function $\eta = 18.47 \times A$ (R^2=0.995) (Supplementary Figure 122).

In order to elucidate the origin of this effect we conducted further investigation.

Carbon nitride particles strongly absorb and scatter light.[ref.[13], Supplementary Figure 17-18 in the ESI therein] Therefore, we concede that the chemical reaction occurs only in a thin layer located close to the surface of the liquid phase. On the other hand, the central area of the batch photoreactor remains in 'dark' and therefore no photocatalytic reaction occurs there. When reaction is performed on 0.05 mmol scale using 2 mL of solvent in a glass tube of 10 mm in

diameter, most of the photoreactor volume is exposed to light, therefore average photoreactor productivity is high. On the other hand, on the 5 mmol scale using 200 mL of solvent in a glass tube of 30 mm in diameter, only a small volume of the batch photoreactor is effectively irradiated by light, while most of the reactor volume remains in dark. As a result average productivity of the photoreactor is low.

In order to find a distance from the reactor wall to the point in the bulk of the reaction mixture below which photons cannot penetrate, we performed light transmittance measurements of K-PHI suspension in $CHCl_3$:DMSO (9:1) in 1 mm, 2 mm, 5 mm and 10 mm cuvettes using convenient UV-vis spectrometer. Ten percent of DMSO has been added to delay sedimentation of K-PHI particles during the measurements.

The data in the graphs (Supplementary Figure 123) show that 1 mm thick layer of K-PHI suspension absorbs 94.50% of blue photons (λ = 461 nm), 2 mm thick layer absorbs 99.15%, 5 mm thick layer absorbs 99.45% and 10 mm thick layer absorbs 99.50% of light. Transmittance measurements were corrected taking into account absorption of pure solvent.

Comparable results were obtained when we measured a fraction of light intensity (461±20 nm, I_0 = 10.6 mW cm^{-2}) passed through the cuvettes of variable length (1 mm, 2 mm, 5 mm and 10 mm) filled with K-PHI suspension.

These reference measurements show that higher light intensity allows for better light delivery in the bulk of the reaction mixture. However, even using relatively high light intensity (10.6 mW cm^{-2}) compared to that in the UV-vis spectrometer, 95% of photons do not penetrate into the reaction mixture deeper than 3 mm.

Having this data, we calculated a specific absolute yield (N) of dichloroketone **2a** (mmol of dichloroketone **2a** produced by 1 gram of K-PHI) assuming that the 'active' volume of the batch reactor is limited by 3 mm thick cylinder shell. Supplementary Figure 124 shows specific absolute yield (N) of dichloroketone **2a** versus enone **1a** amount (n, mmol) taken for the experiment.

In this case the yield of **2a** is 7.05±0.5 mmol g^{-1} and depends weakly on the amount of enone **1a** taken for the experiment. Relative standard deviation in this case is 7.2%.

All in all, we conclude that the main limitation of scaling up the photocatalytic reaction mediated by the heterogeneous photocatalyst in batch is poor light penetration into the bulk of the reaction mixture.

Supplementary Note 7

To further investigate colloidal properties of K-PHI nanoparticles, we conducted a deposition test, to compare its properties with other carbon nitrides, such as g-CN and mpg-CN. We proceeded by adding the selected photocatalyst (5 mg) in a mixture of $CHCl_3$/DMSO (3:2, 2 mL), sonicated for 10 minutes and stirred for another 10 minutes. Suspensions were left standing without agitation. Pictures were taken at different times to observe solid precipitation (Supplementary Figure 125). From this experiment it clear as K-PHI also has superior capacity to stay dispersed in solution.

Supplementary Figures

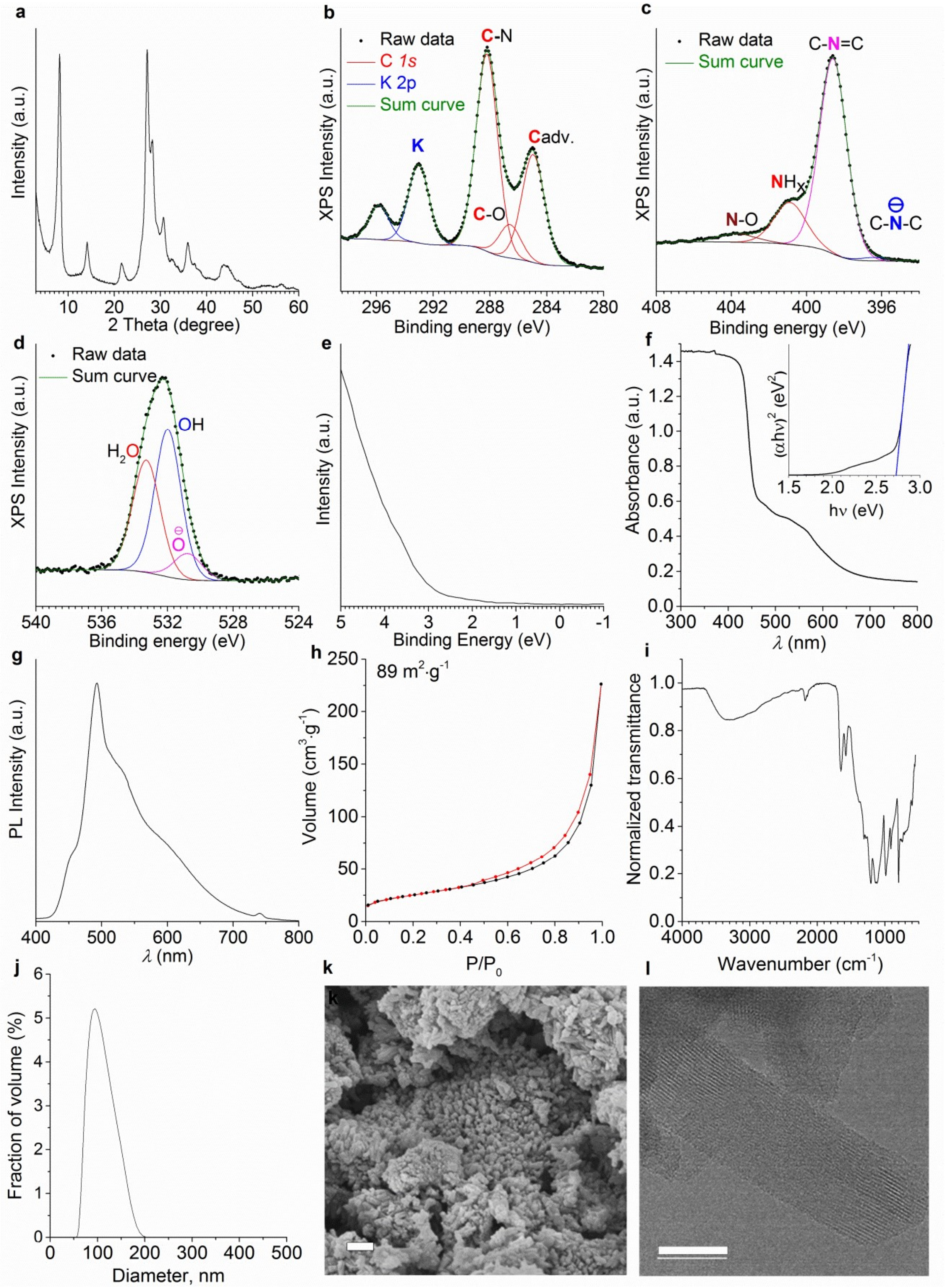

Supplementary Figure 1.[14] K-PHI characterization. a) PXRD pattern of K-PHI; b) XPS C 1s and K 2p spectra of K-PHI; c) XPS N 1s spectrum of K-PHI; d) XPS O 1s spectrum of K-PHI; e) UPS spectrum of K-PHI; f) UV-vis absorption spectrum of K-PHI with Tauc plot as inset assuming that K-PHI is a direct semiconductor; g) room temperature PL spectrum of K-PHI obtained upon excitation with

350 nm wavelength; h) N_2 sorption isotherm measured at 77 K. BET surface area; i) FT-IR spectrum of K-PHI; j) DLS analysis of K-PHI suspension in water; k) representative SEM image of K-PHI photocatalyst. Scale bar 200 nm; l) AC-HRTEM image of K-PHI photocatalyst. Scale bar 20nm. Source data are provided as a Source Data file.

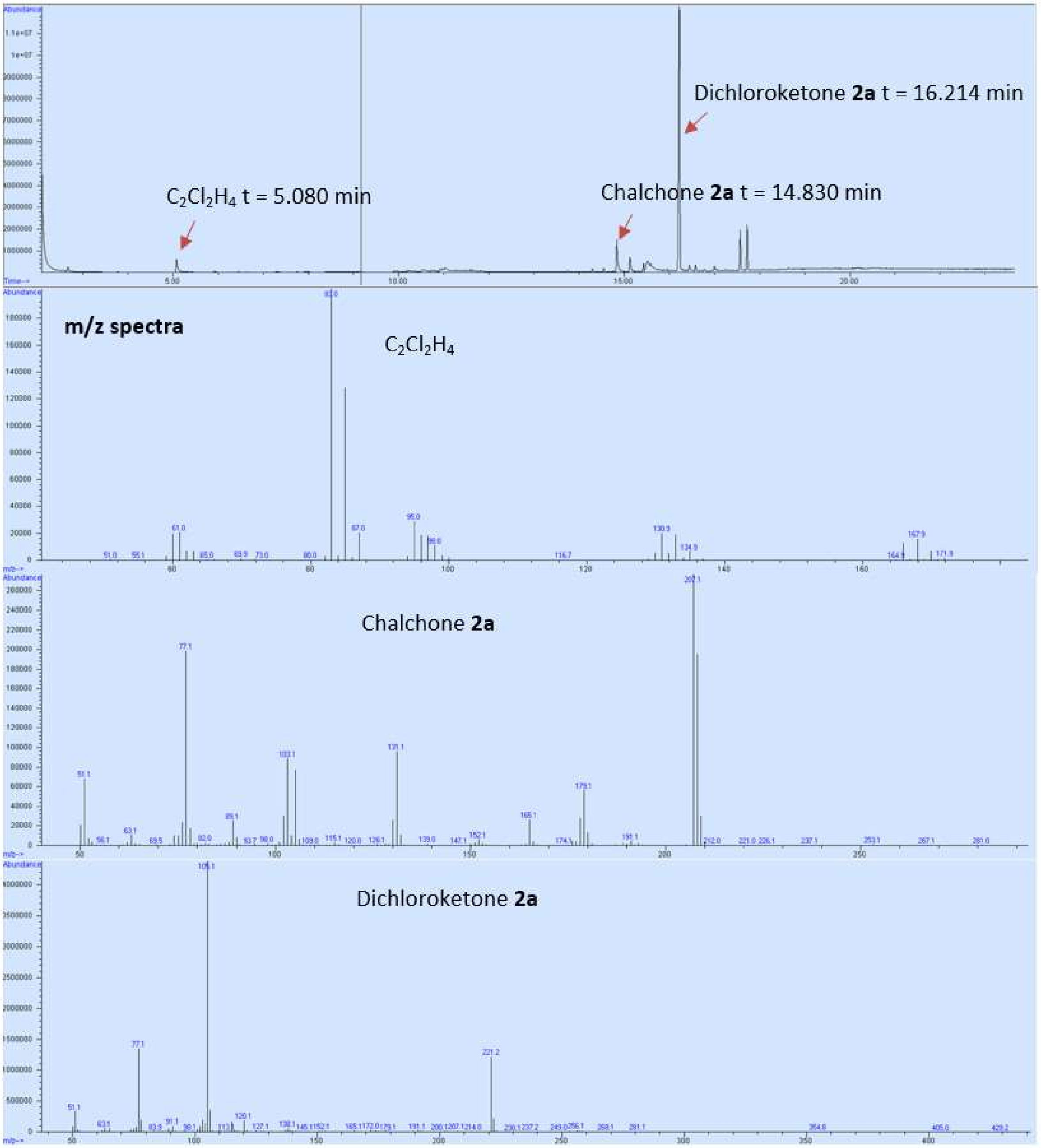

Supplementary Figure 2. Typical chromatogram during reaction screening

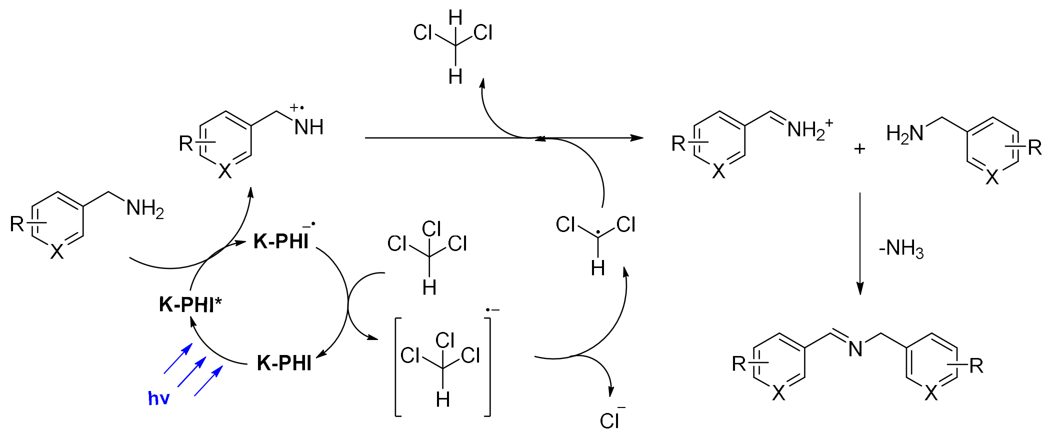

Supplementary Figure 3. Detailed mechanism proposed for oxidative coupling of benzylamines.

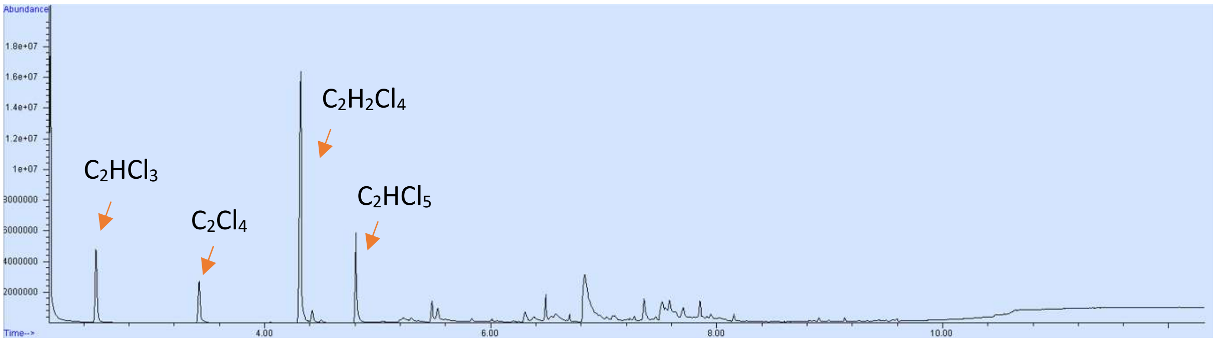

Supplementary Figure 4. Chromatogram after reaction of entry 2 (Supplementary Table 4).

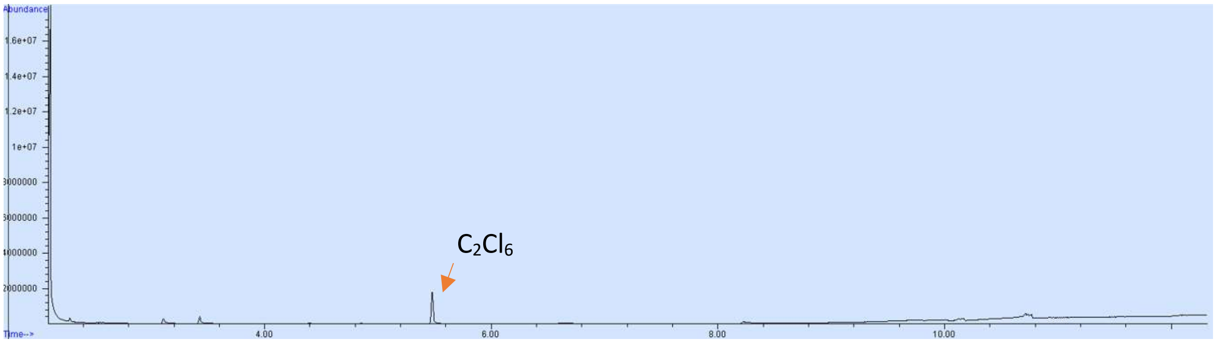

Supplementary Figure 5. Chromatogram after reaction of entry 3 (Supplementary Table 4).

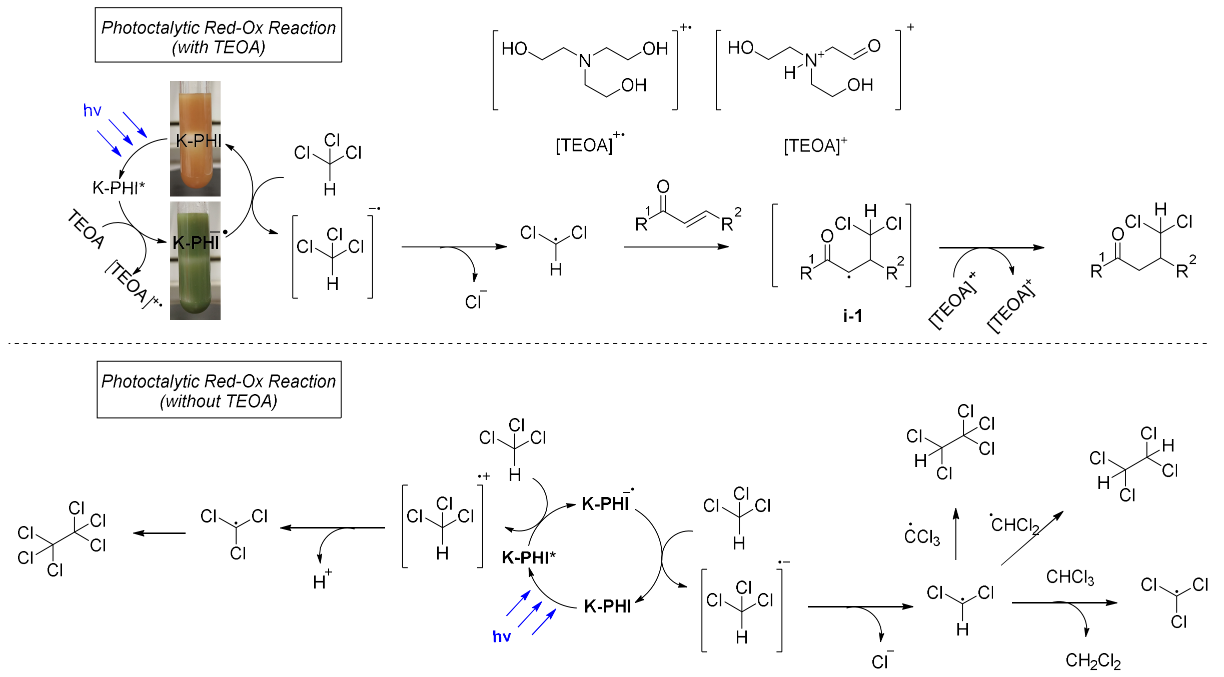

Supplementary Figure 6. Detaild mechanism proposed for dichloromethyl radical addition to enones and also for the formation of the different halogenated hydrocarbons.

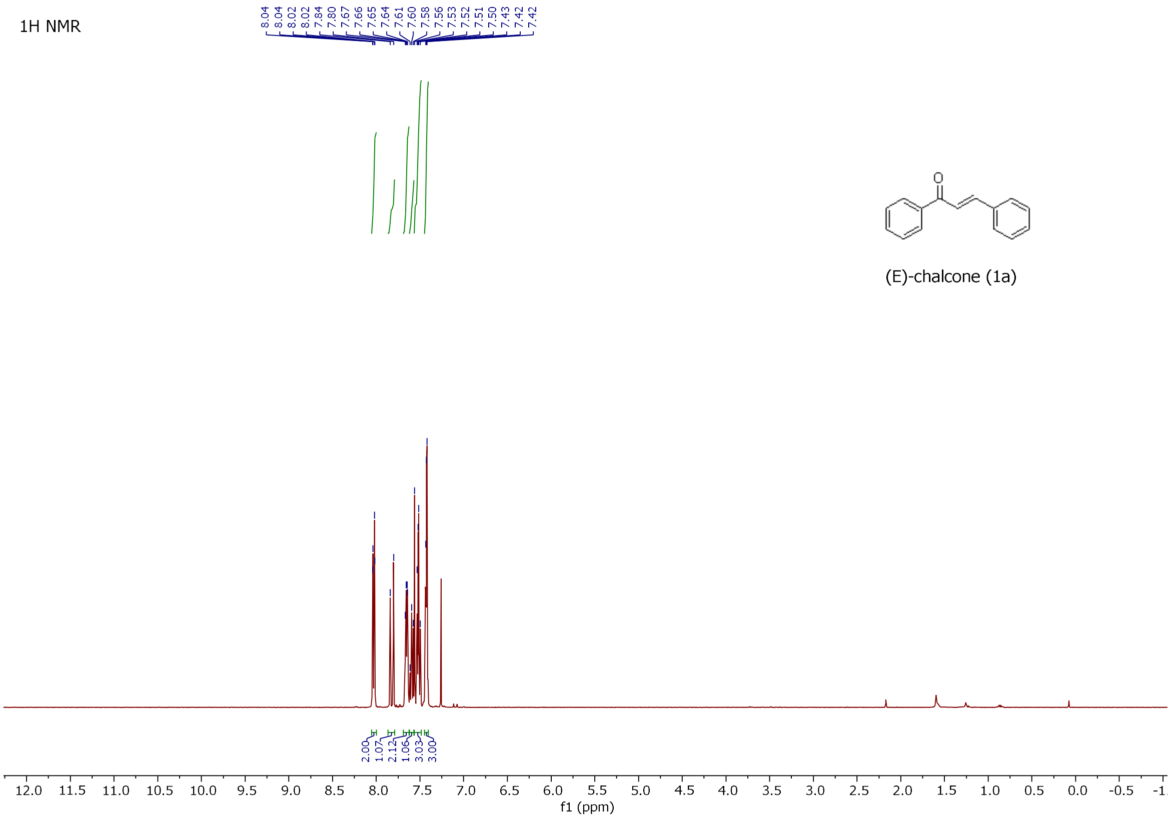

Supplementary Figure 7. NMR spectrum of 1a.

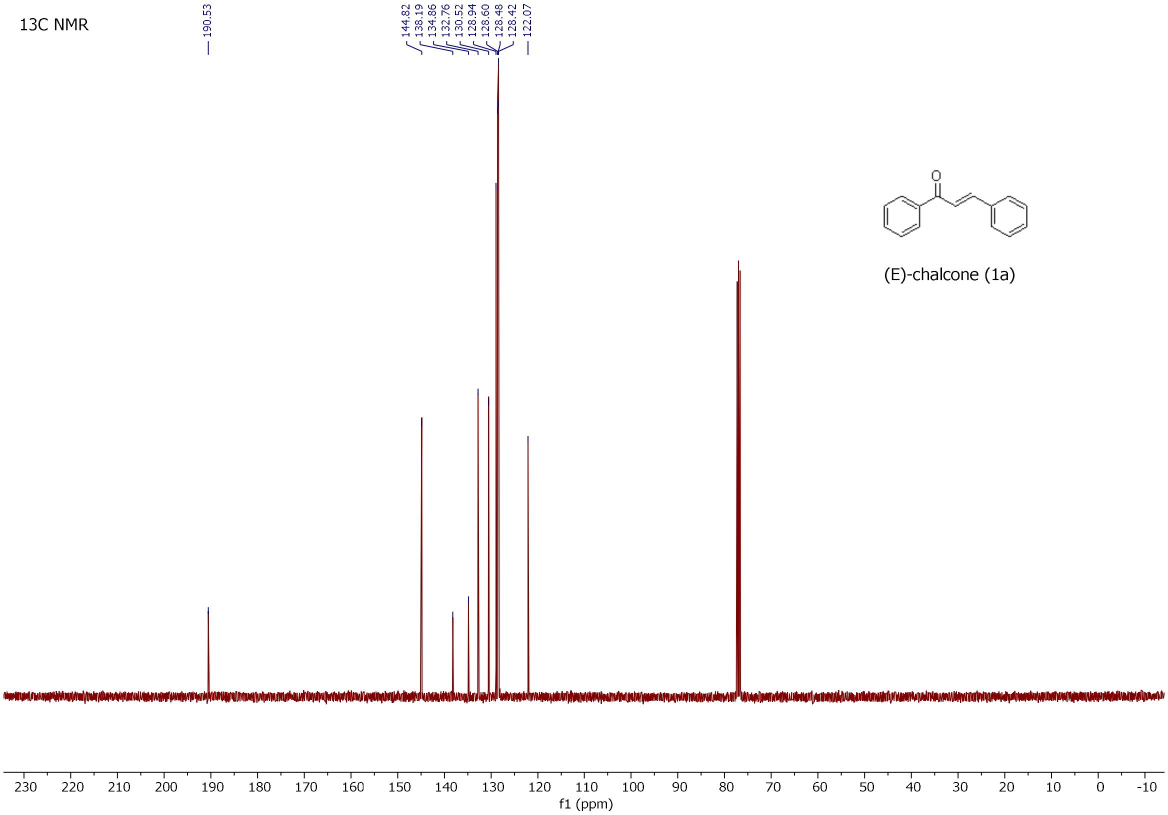

Supplementary Figure 8. NMR spectrum of 1a.

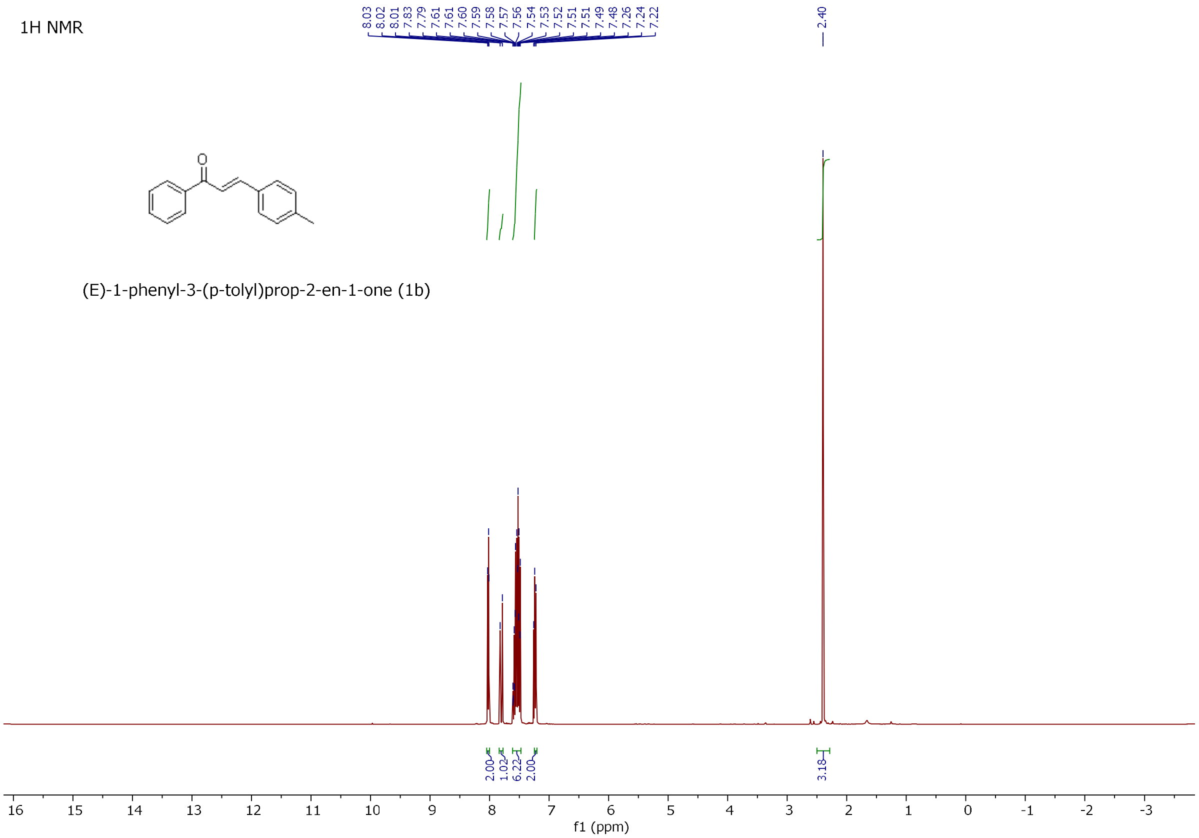

Supplementary Figure 9. NMR spectrum of **1b**.

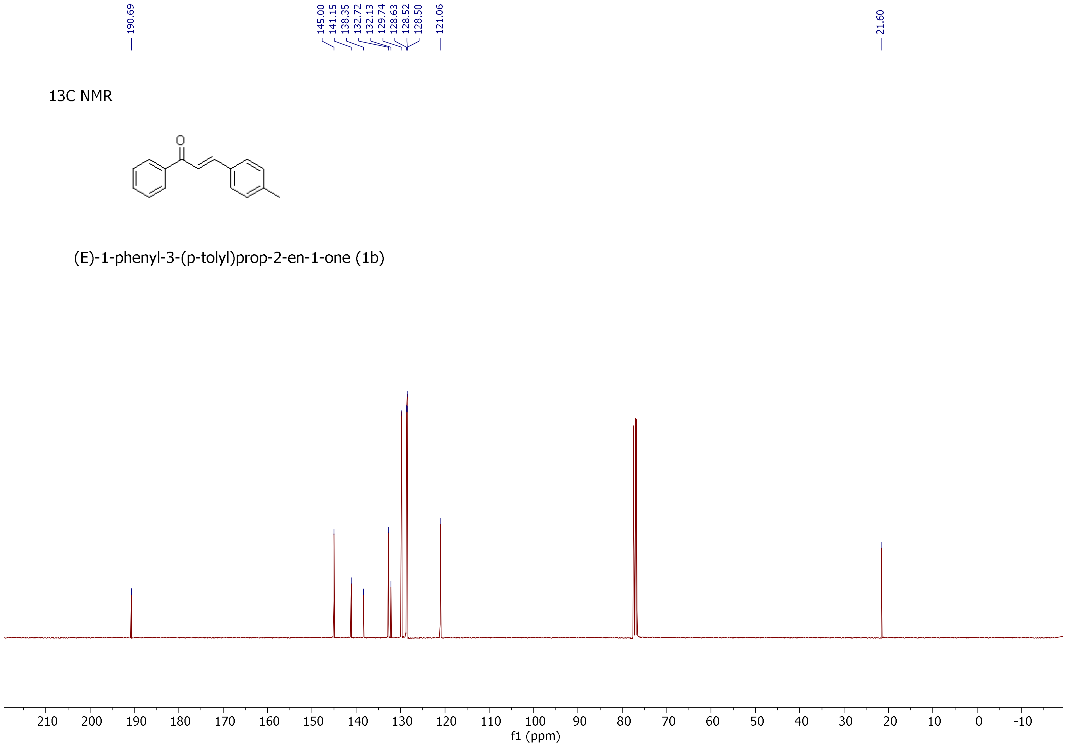

Supplementary Figure 10. NMR spectrum of **1b**.

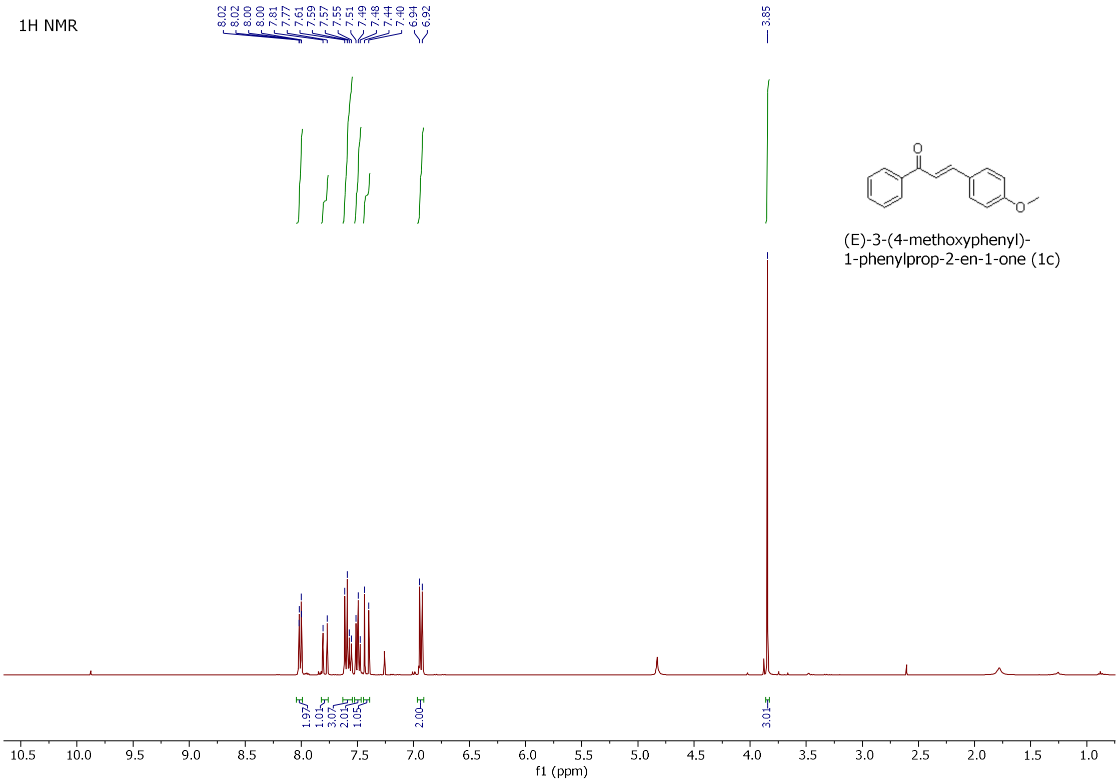

Supplementary Figure 11. NMR spectrum of 1c.

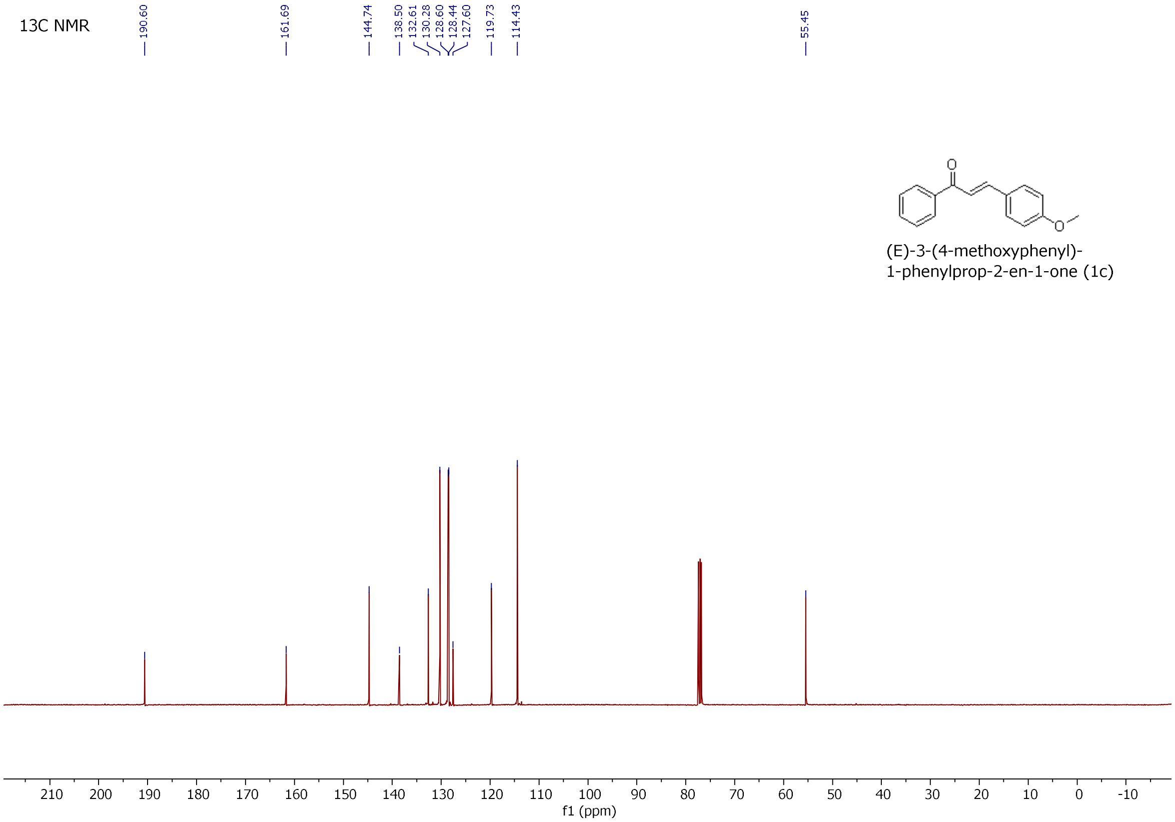

Supplementary Figure 12. NMR spectrum of 1c.

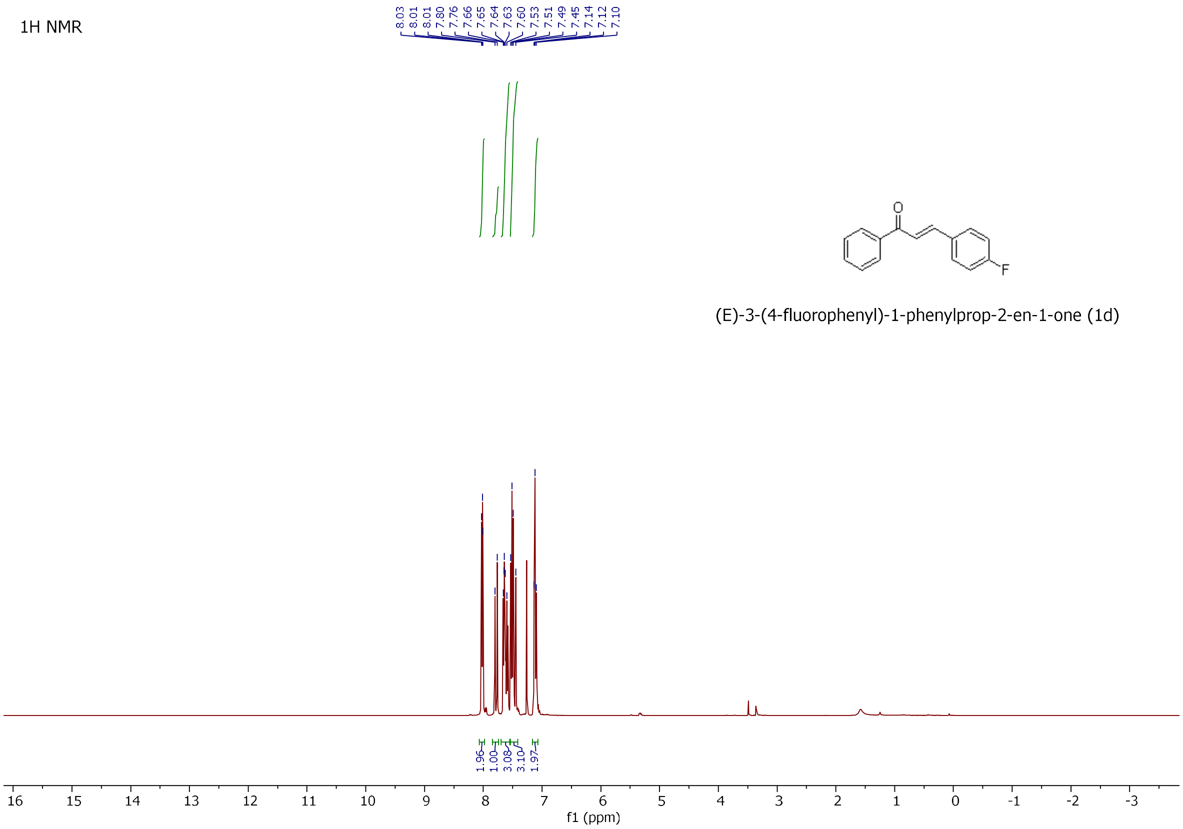

Supplementary Figure 13. NMR spectrum of **1d**.

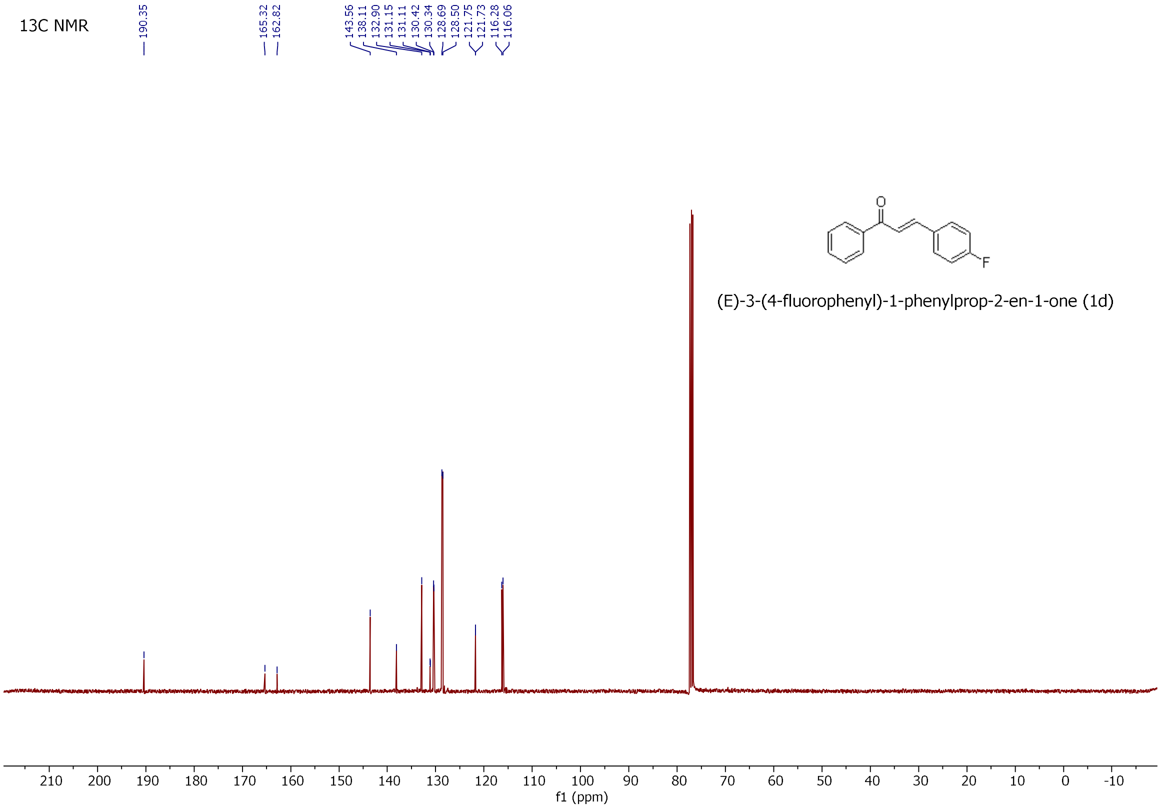

Supplementary Figure 14. NMR spectrum of **1d**.

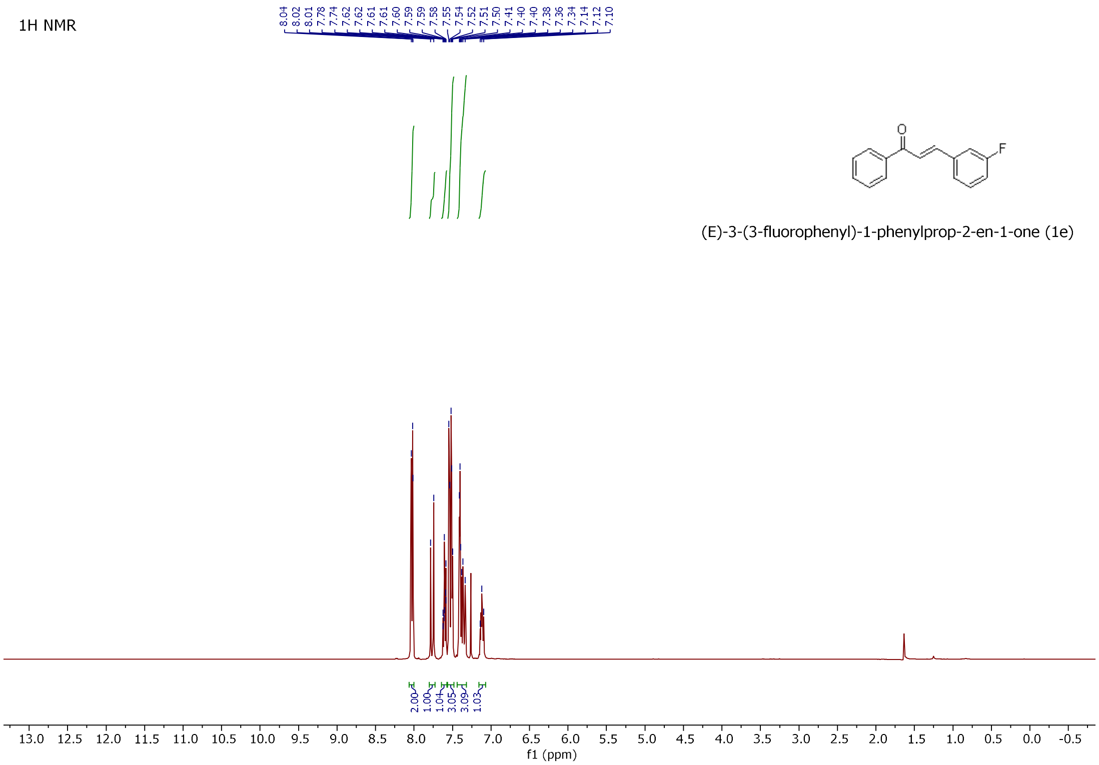

Supplementary Figure 15. NMR spectrum of **1e**.

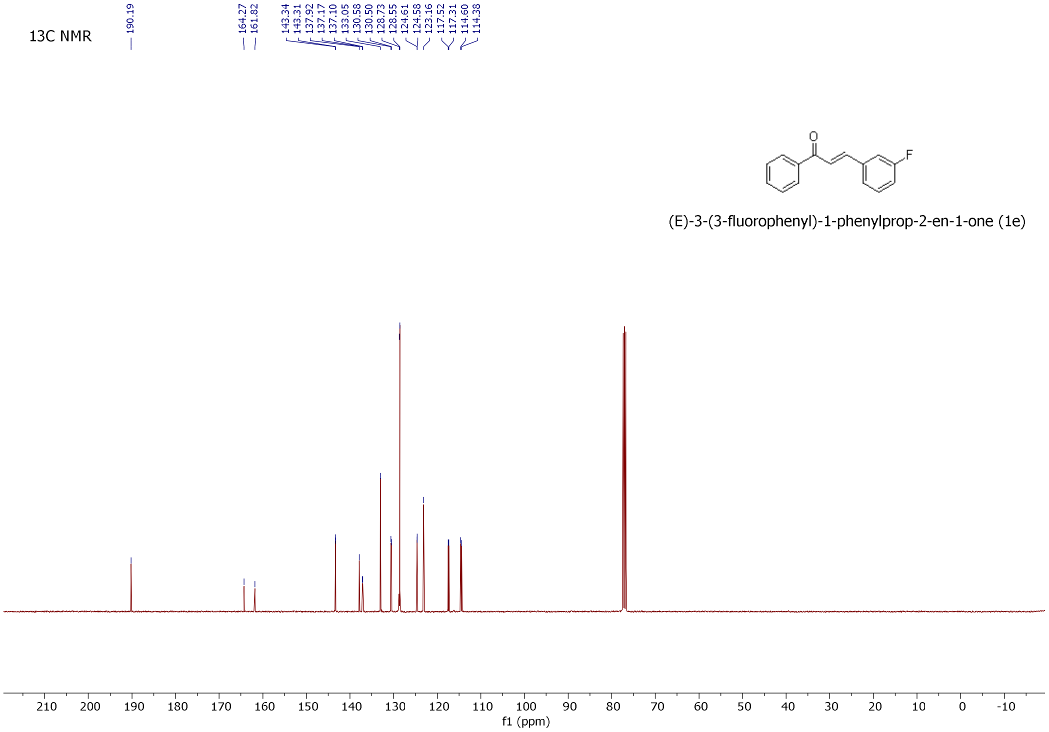

Supplementary Figure 16. NMR spectrum of **1e**.

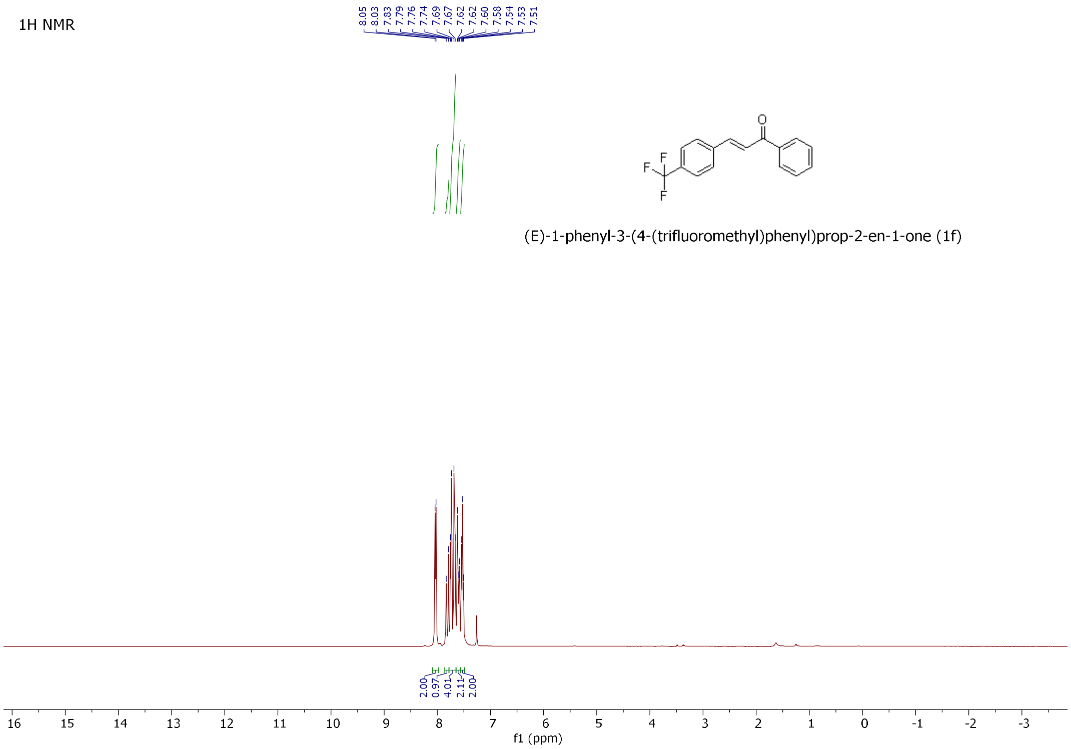

Supplementary Figure 17. NMR spectrum of 1f.

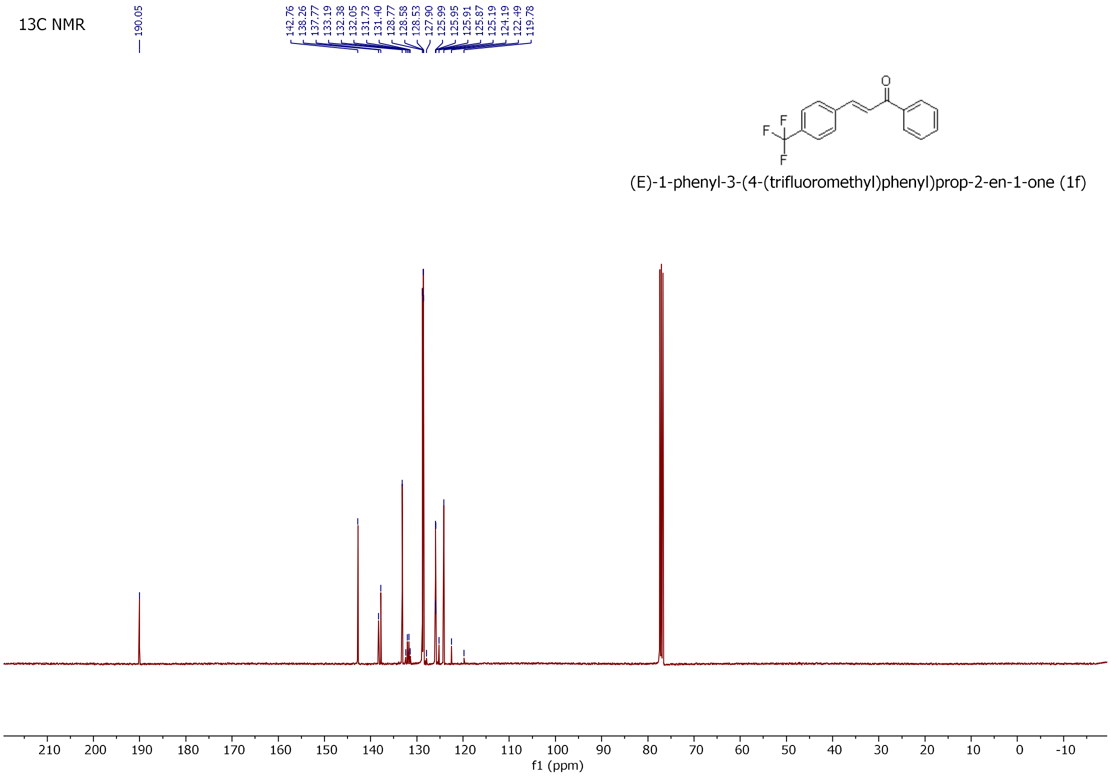

Supplementary Figure 18. NMR spectrum of 1f.

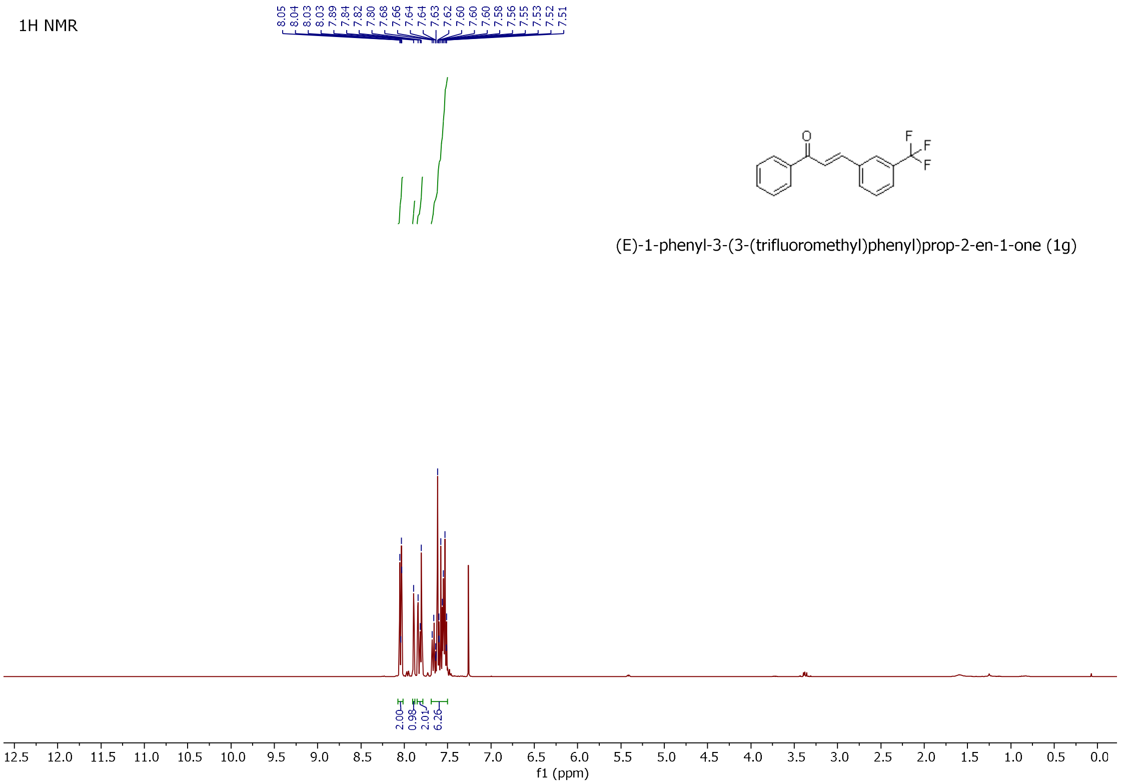

Supplementary Figure 19. NMR spectrum of **1g**.

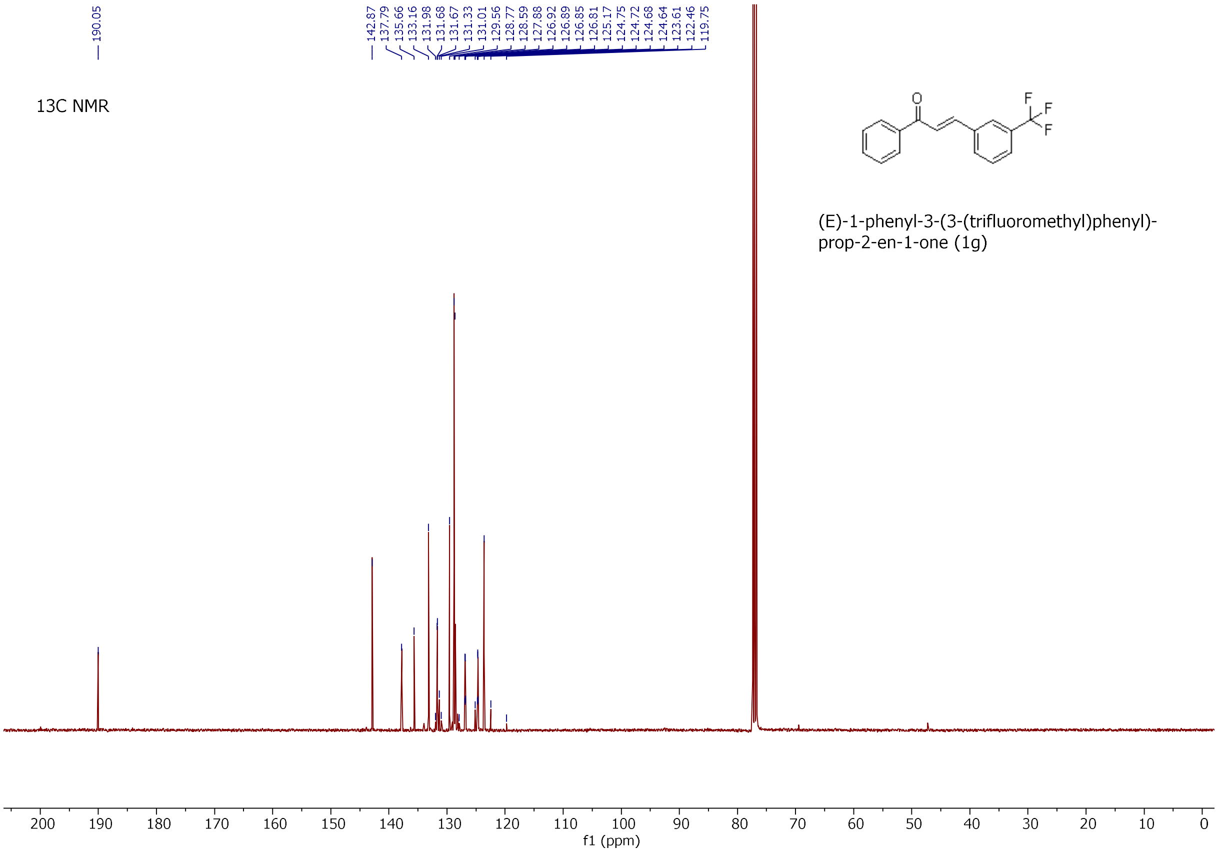

Supplementary Figure 20. NMR spectrum of **1g**.

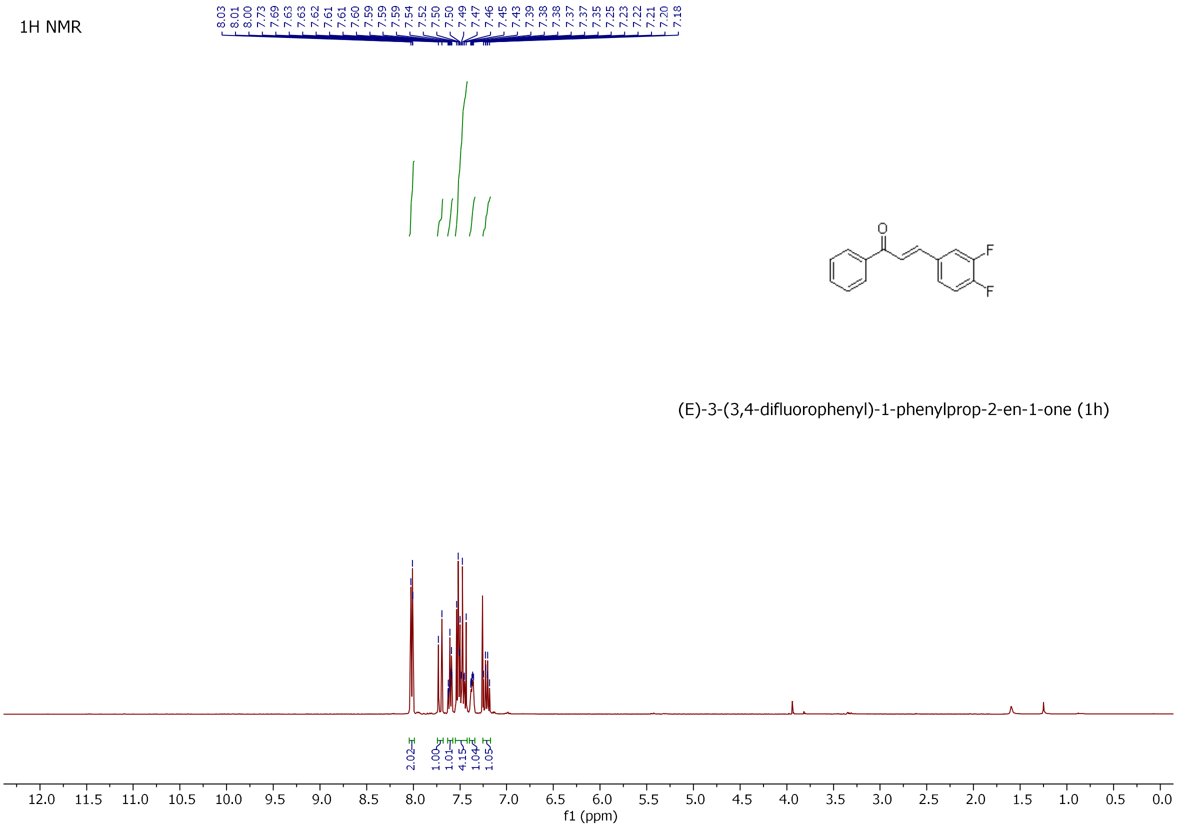

Supplementary Figure 21. NMR spectrum of **1h**.

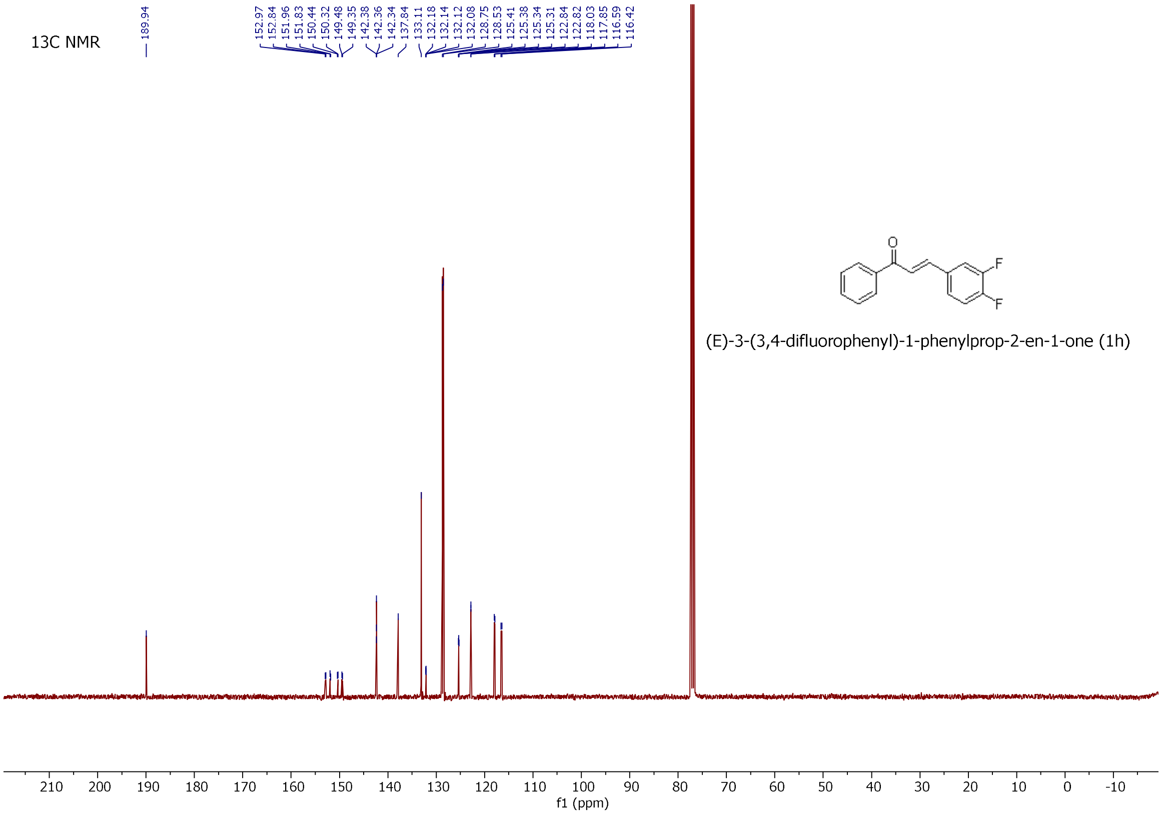

Supplementary Figure 22. NMR spectrum of **1h**.

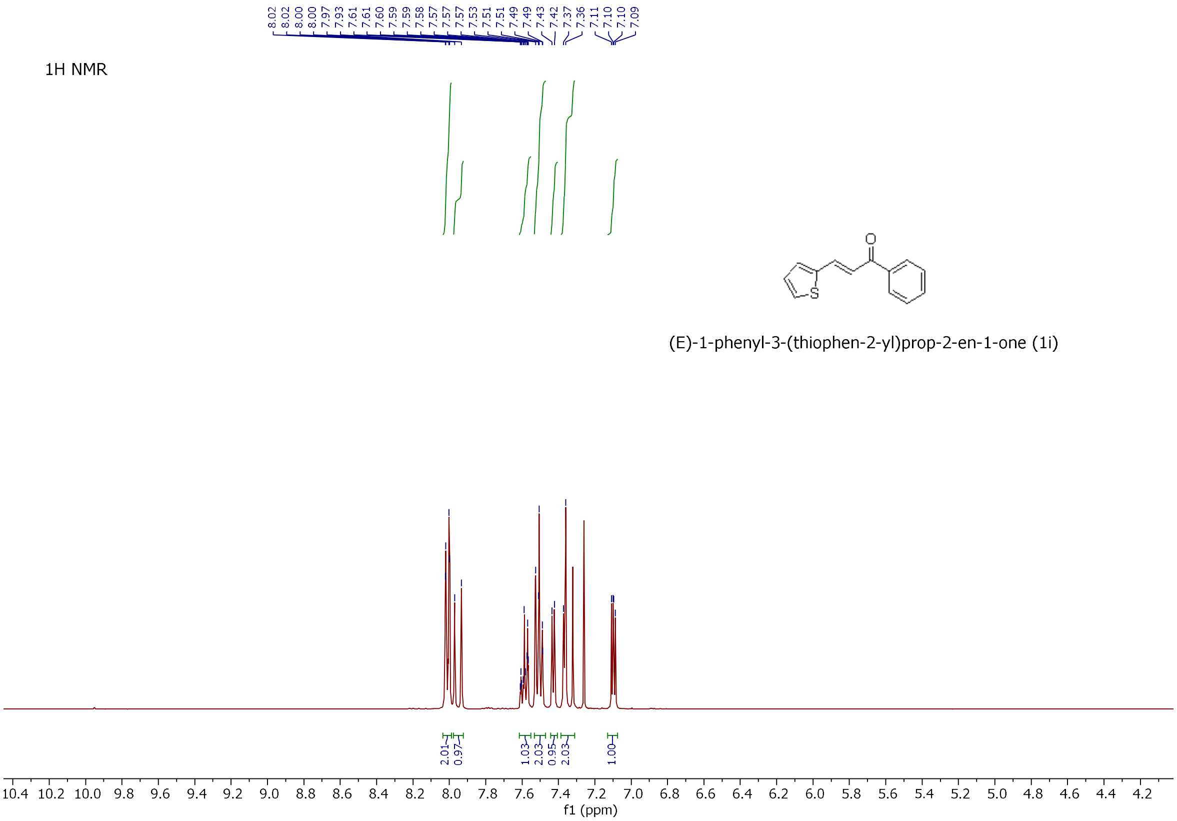

Supplementary Figure 23. NMR spectrum of 1i.

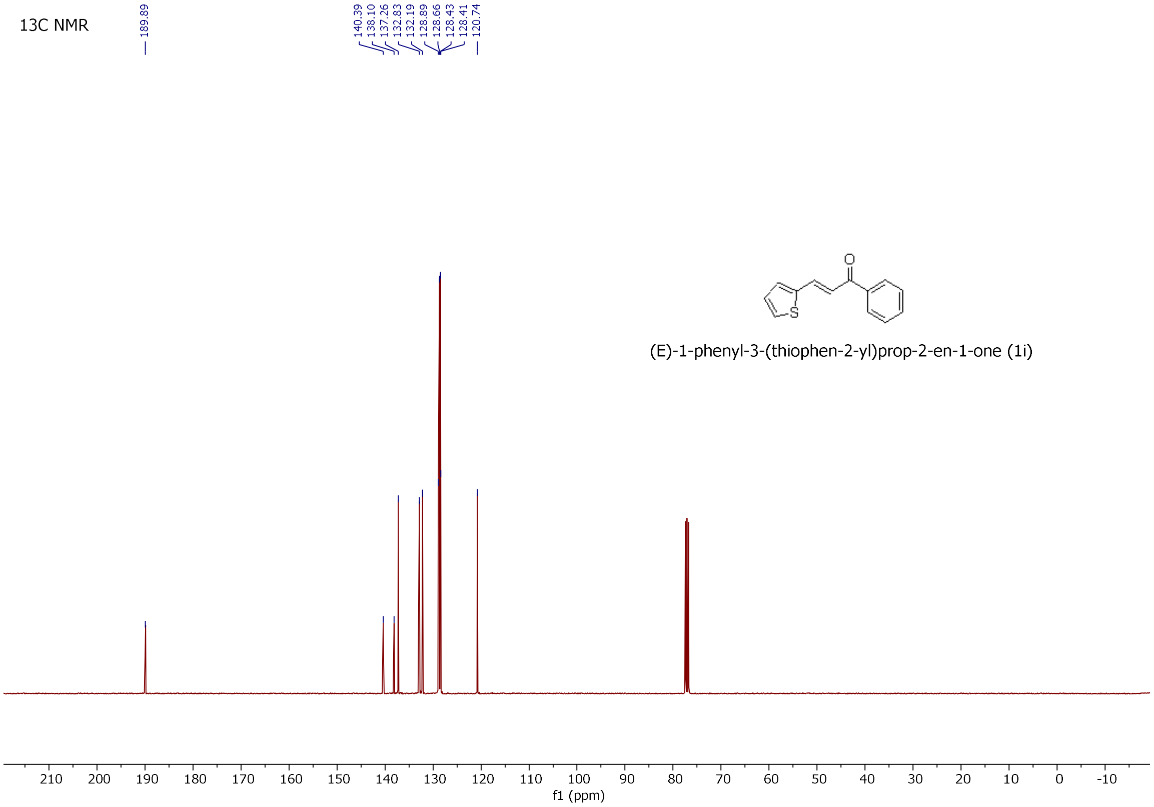

Supplementary Figure 24. NMR spectrum of 1i.

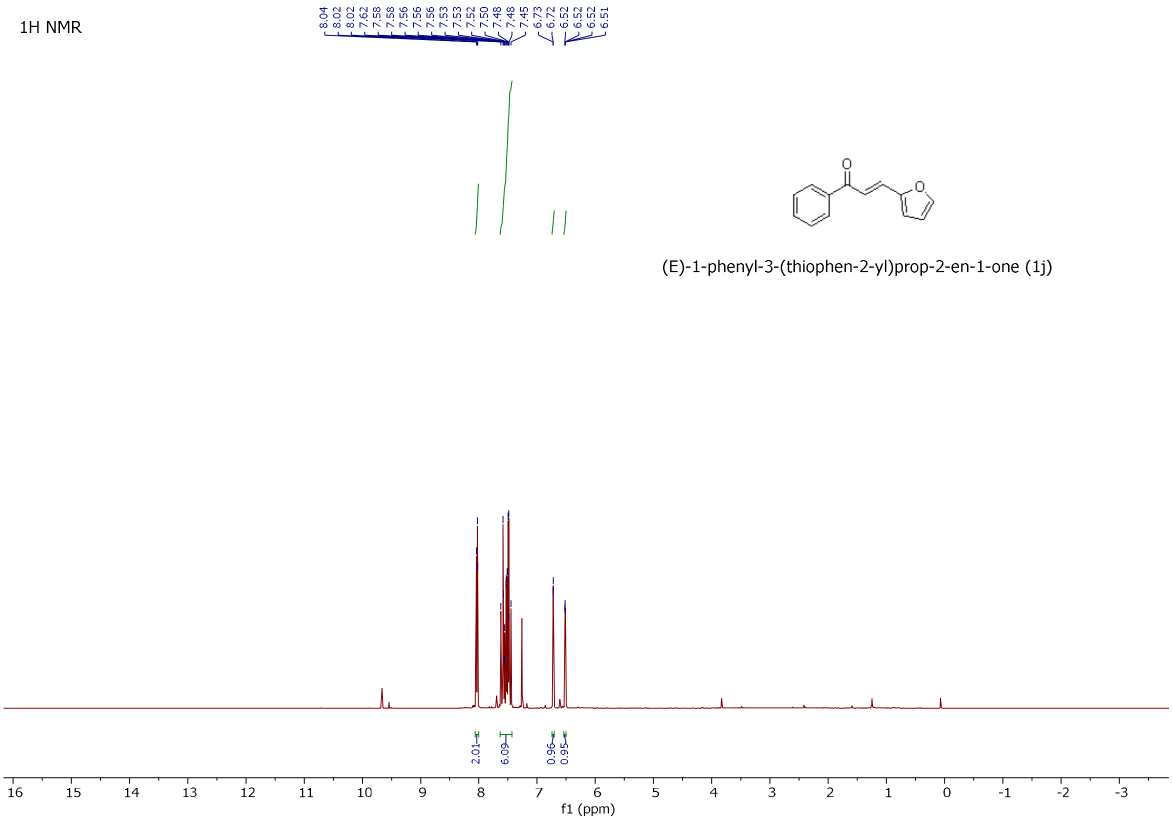

Supplementary Figure 25. NMR spectrum of 1j.

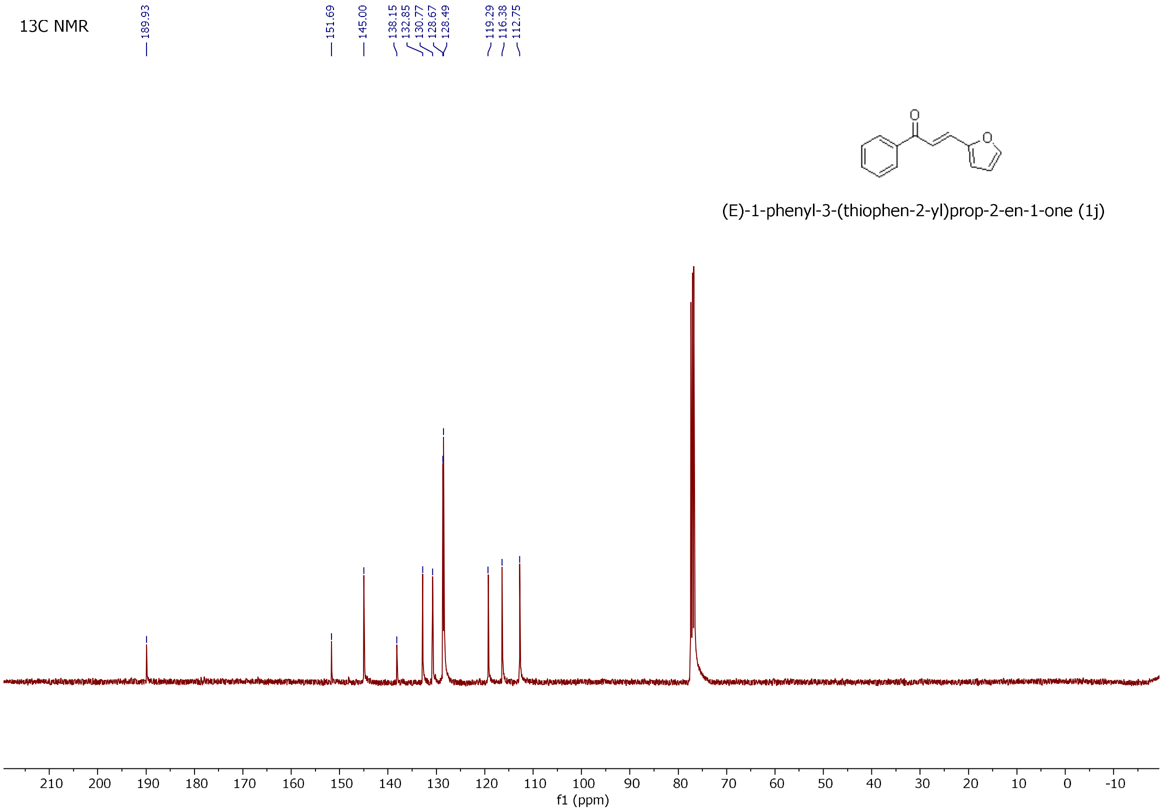

Supplementary Figure 26. NMR spectrum of 1j.

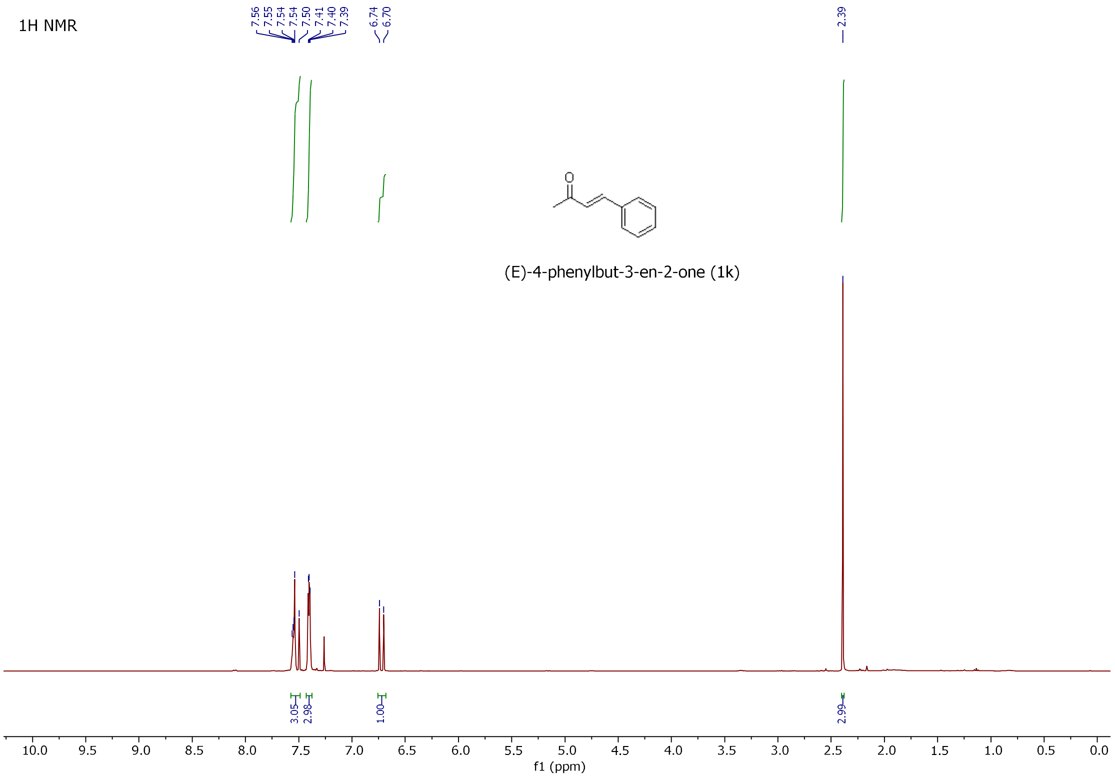

Supplementary Figure 27. NMR spectrum of 1k.

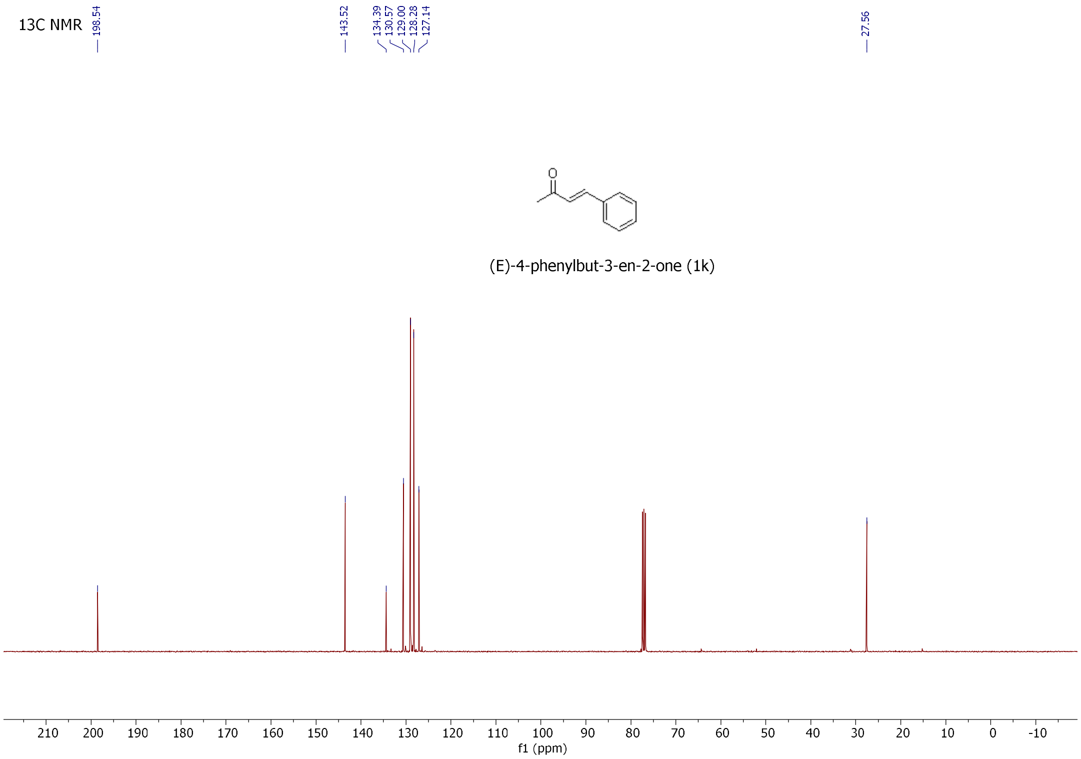

Supplementary Figure 28. NMR spectrum of 1k.

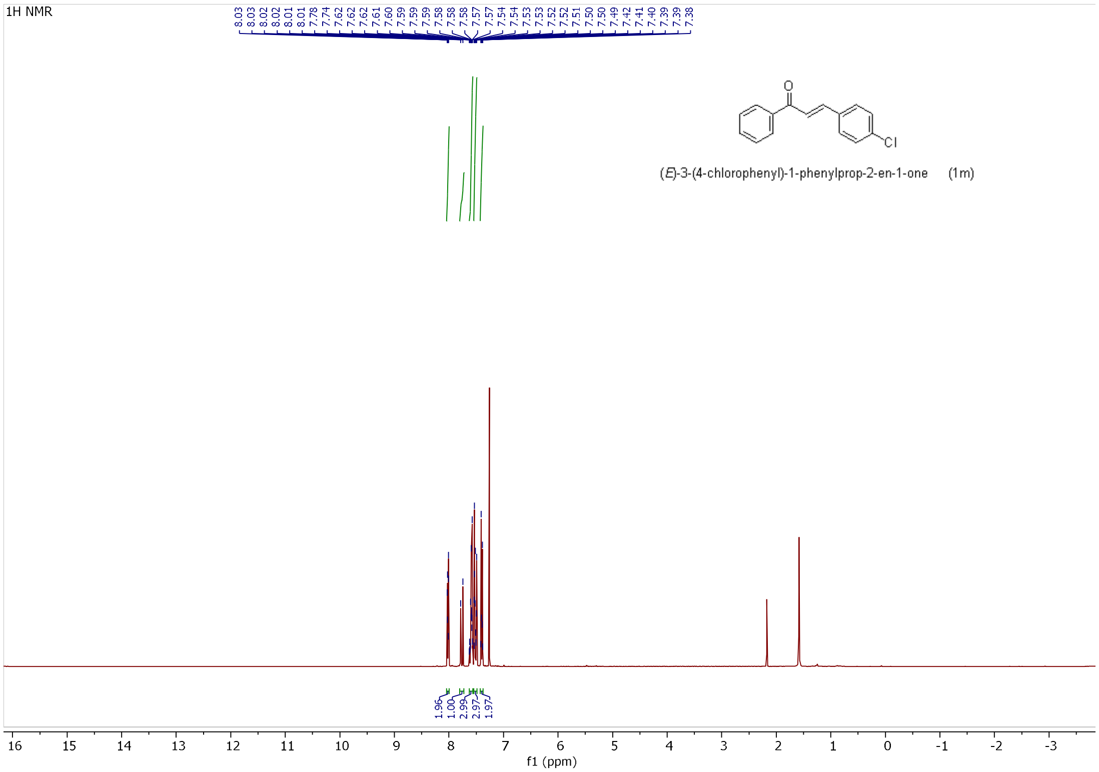

Supplementary Figure 29. NMR spectrum of **1m**.

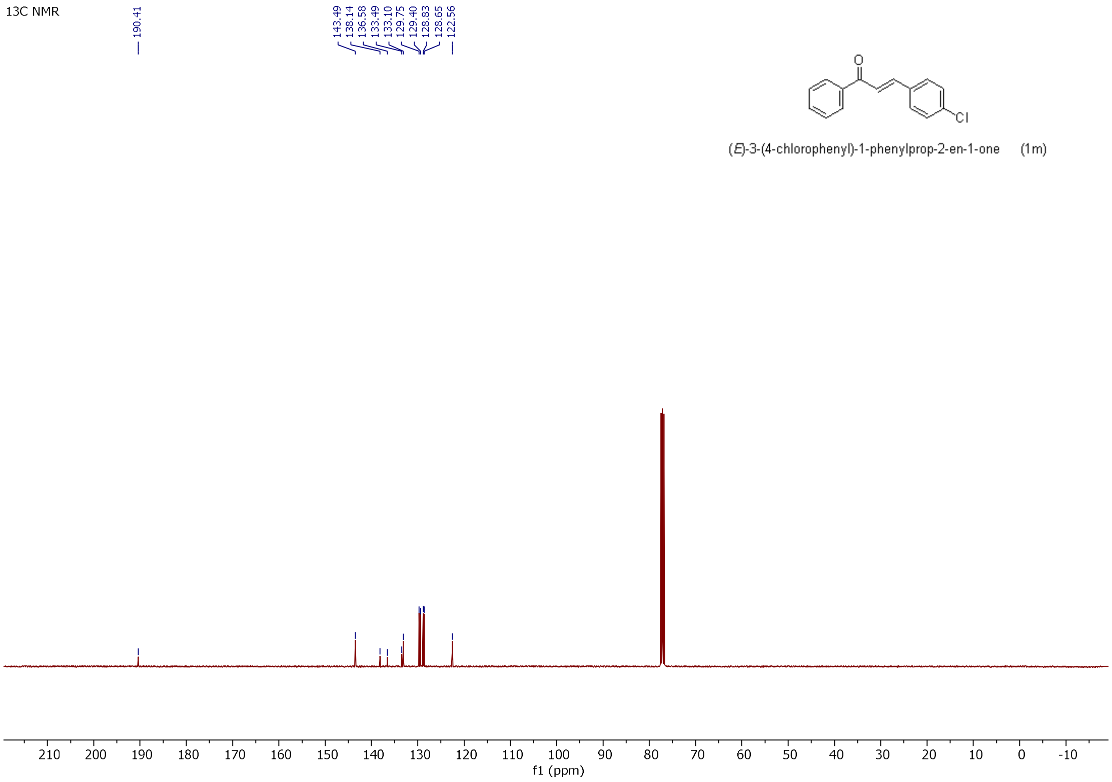

Supplementary Figure 30. NMR spectrum of **1m**.

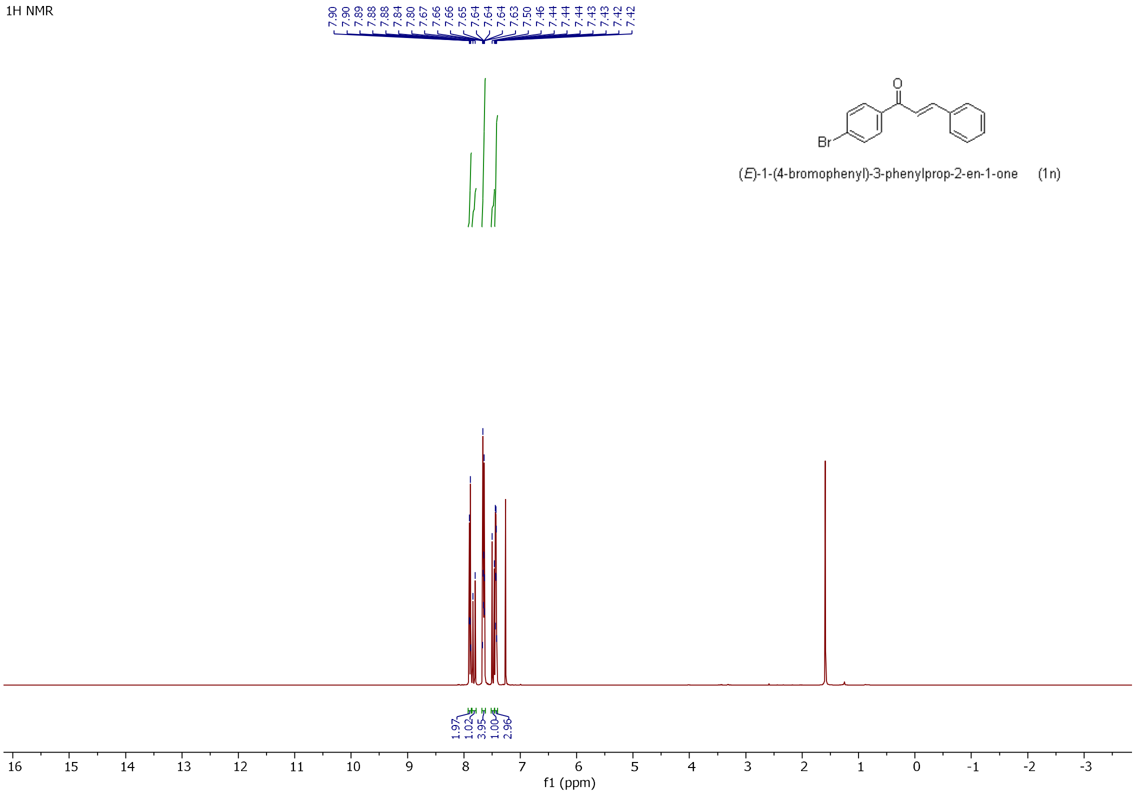

Supplementary Figure 31. NMR spectrum of 1n.

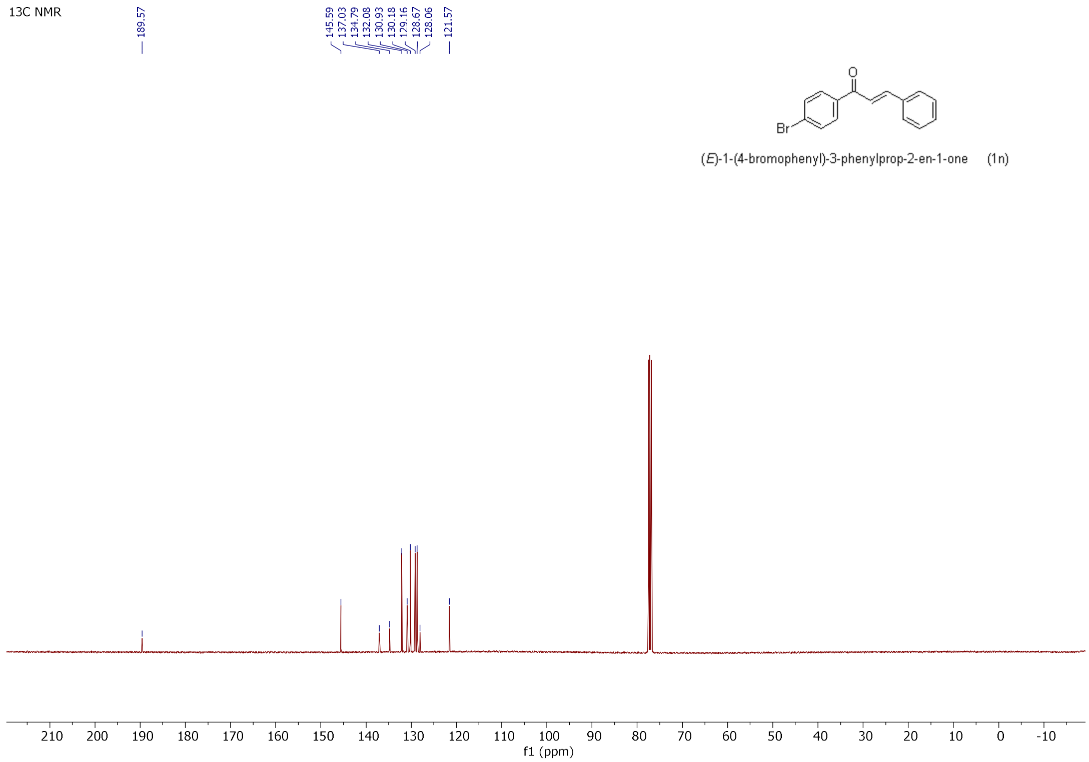

Supplementary Figure 32. NMR spectrum of 1n.

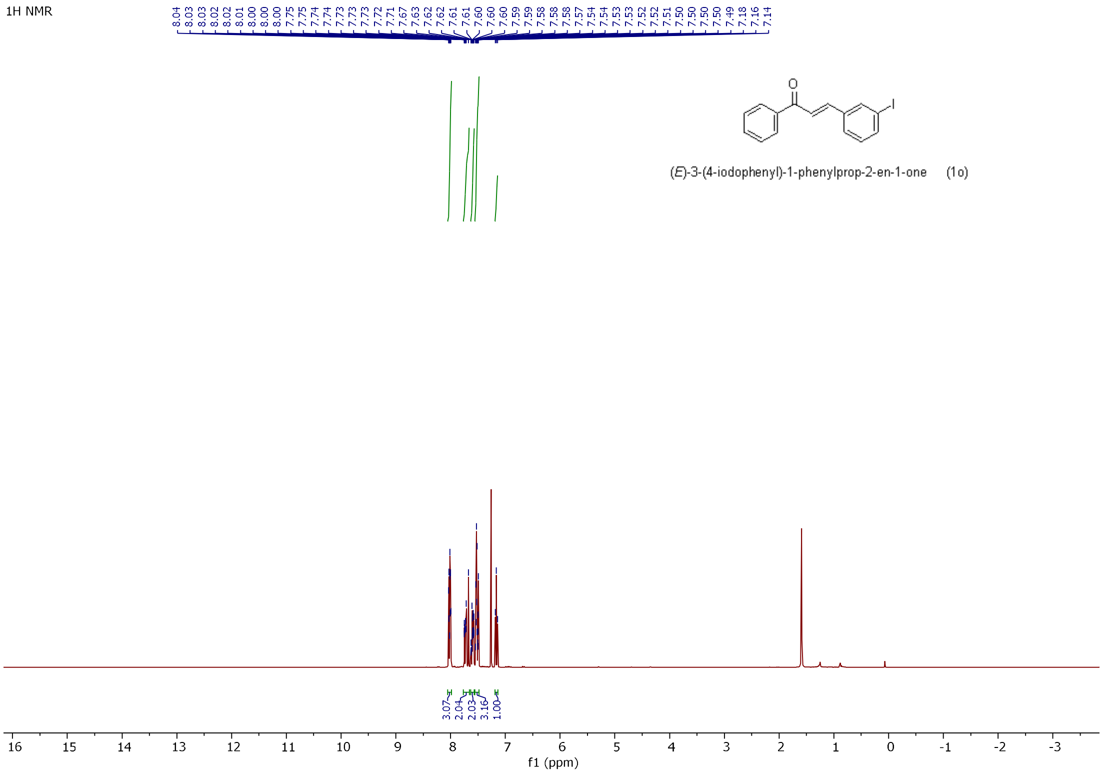

Supplementary Figure 33. NMR spectrum of 1o.

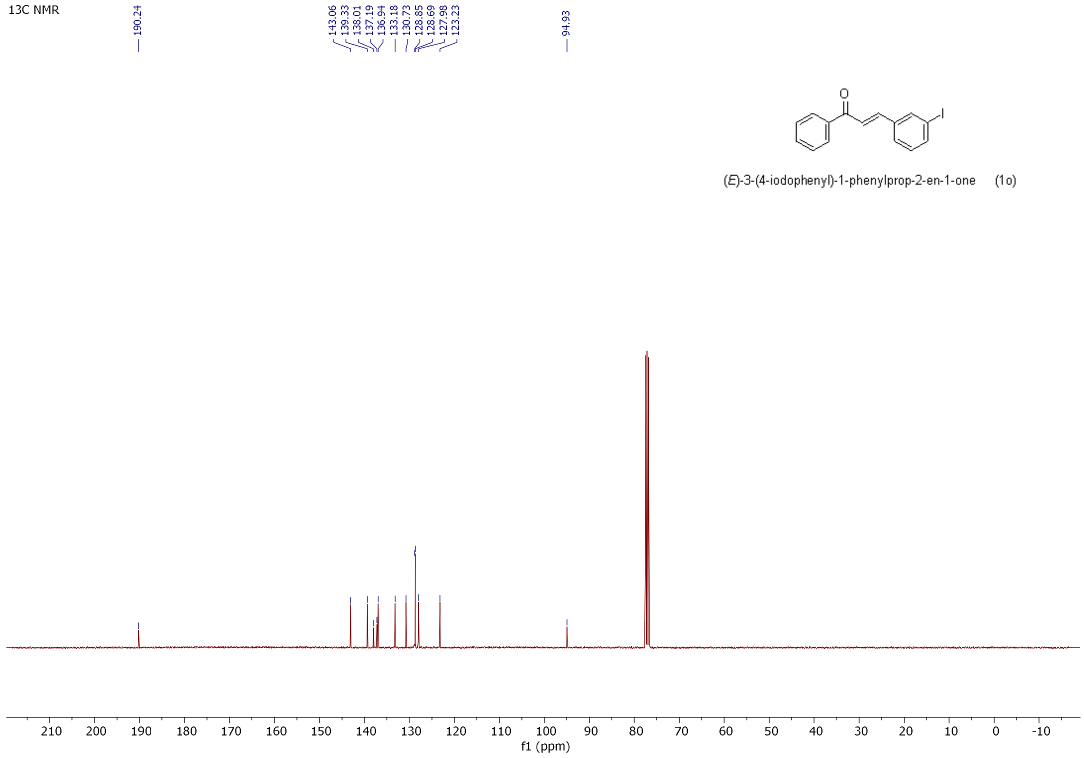

Supplementary Figure 34. NMR spectrum of 1o.

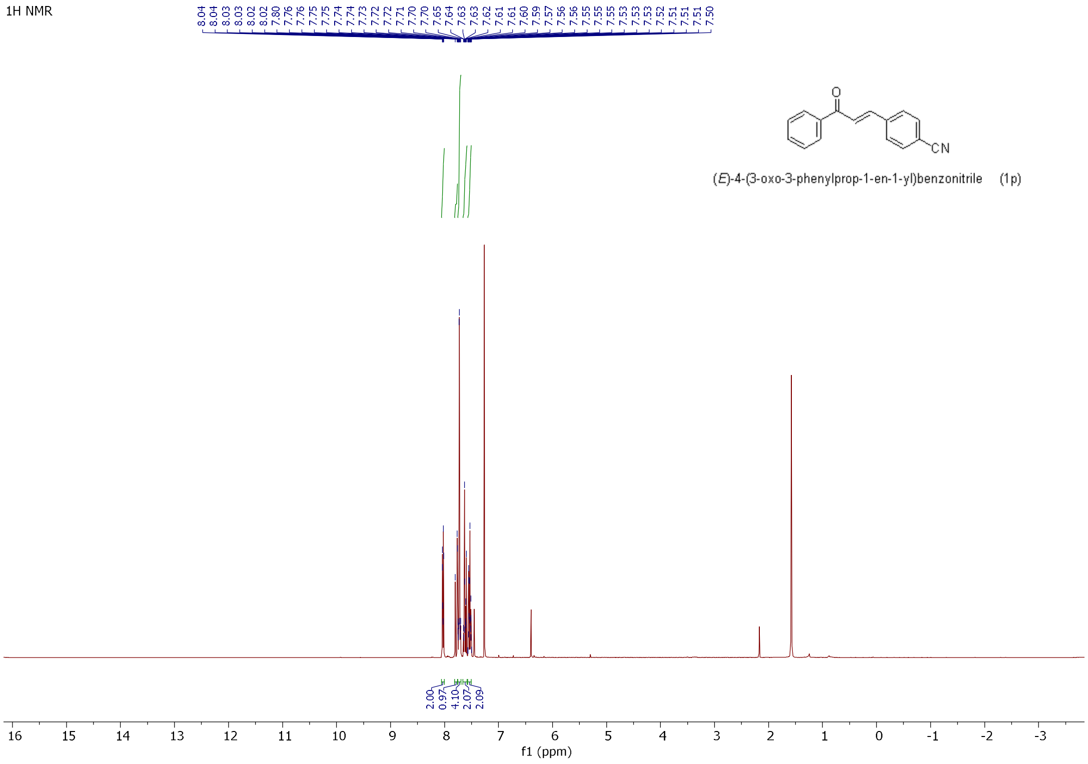

Supplementary Figure 35. NMR spectrum of **1p**.

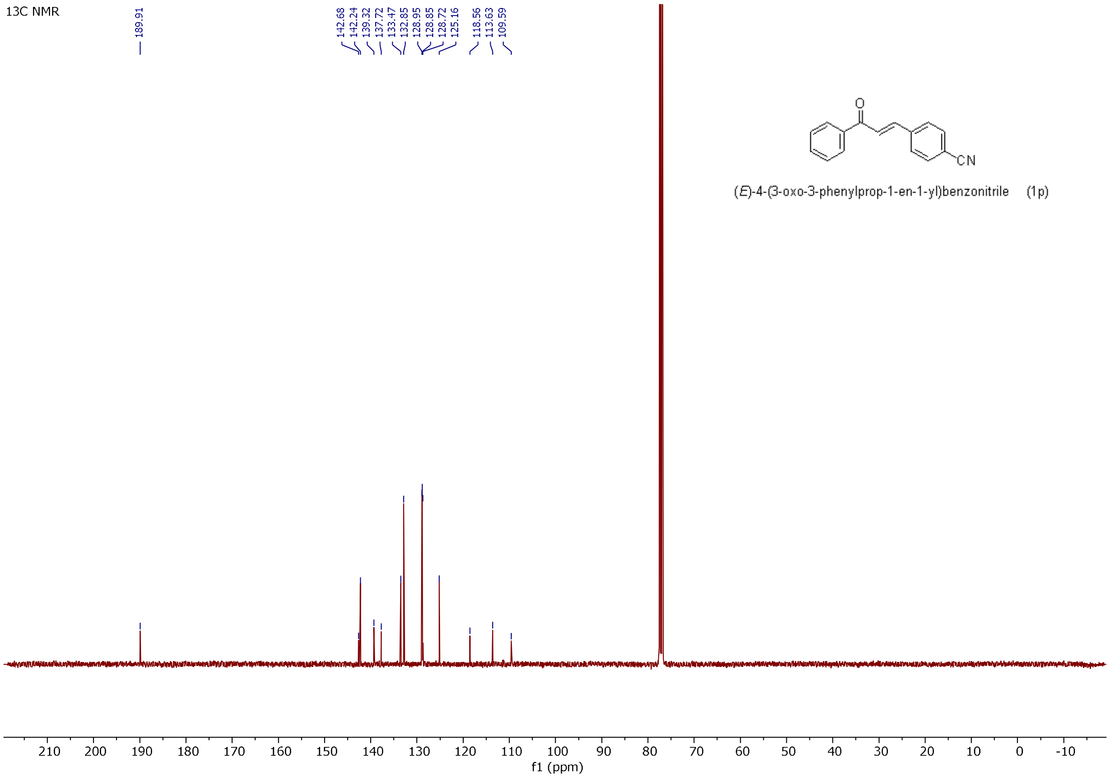

Supplementary Figure 36. NMR spectrum of **1p**.

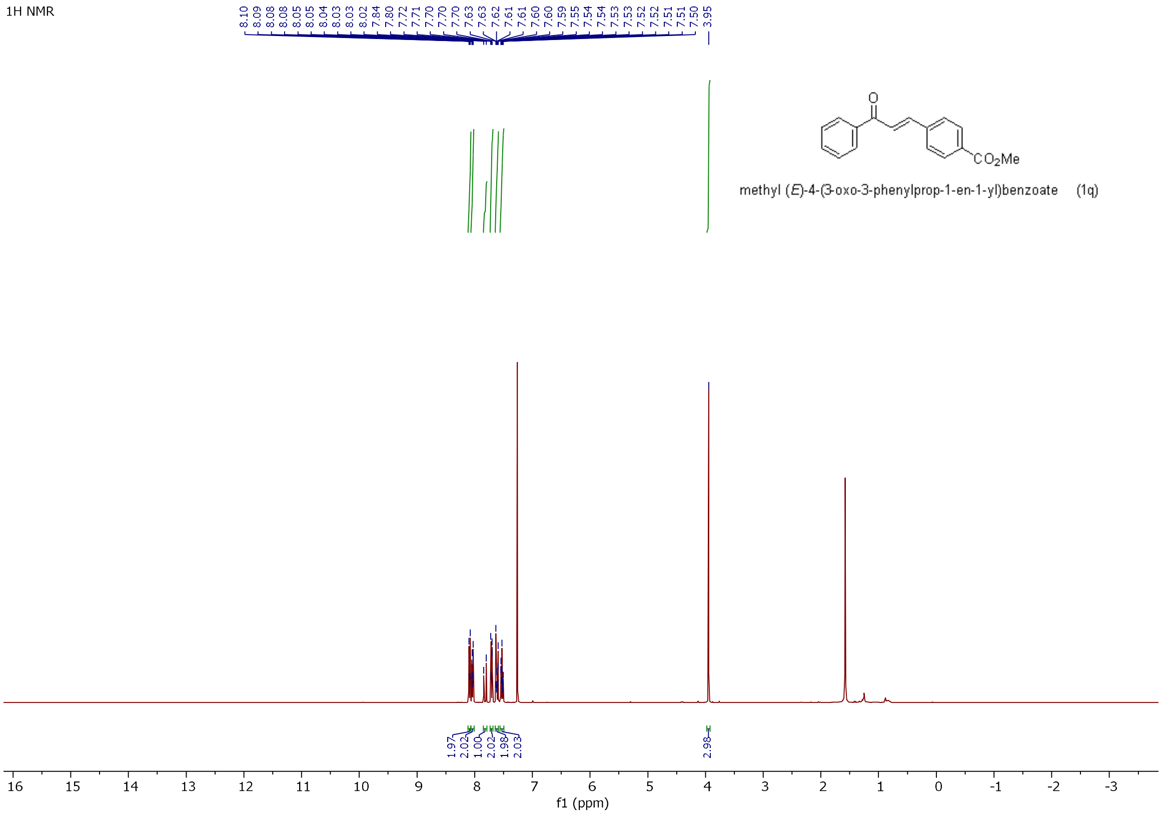

Supplementary Figure 37. NMR spectrum of **1q**.

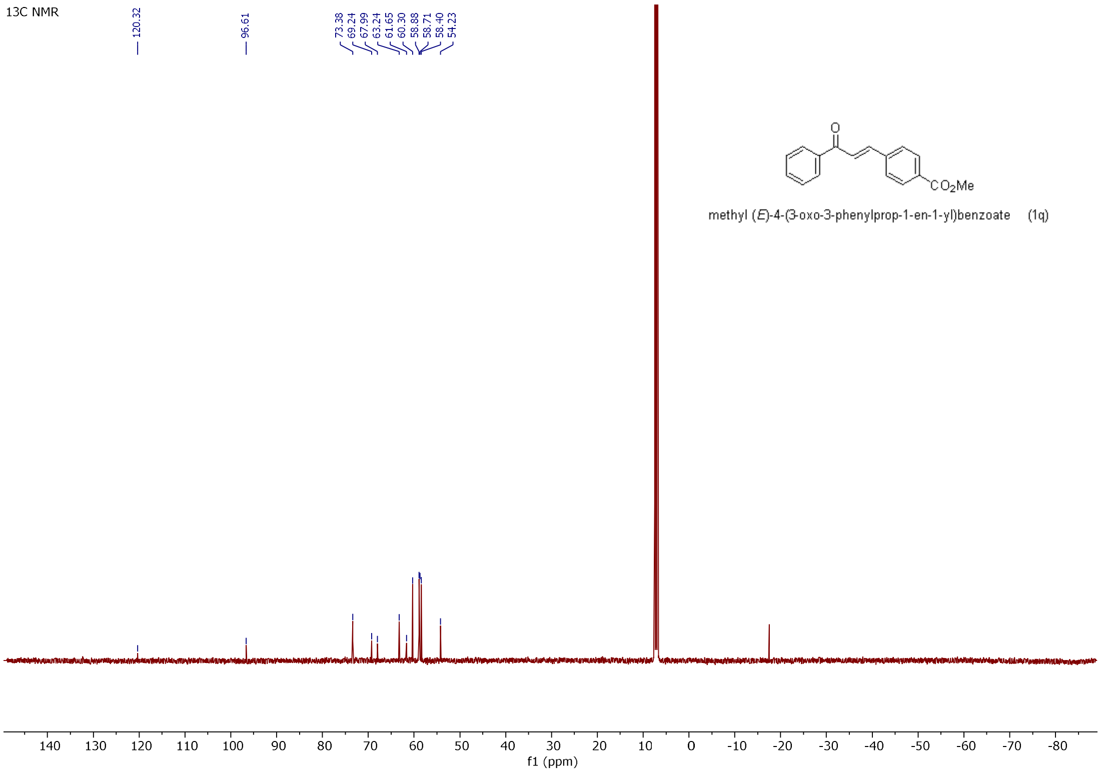

Supplementary Figure 38. NMR spectrum of **1q**.

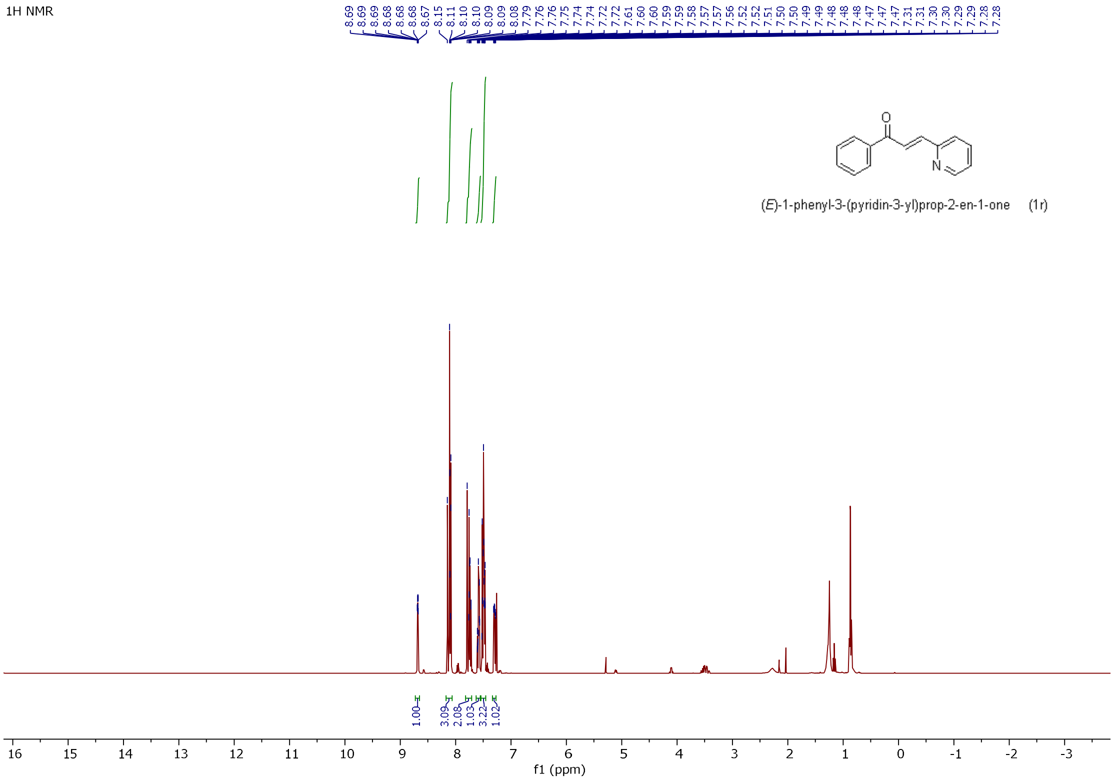

Supplementary Figure 39. NMR spectrum of **1r**.

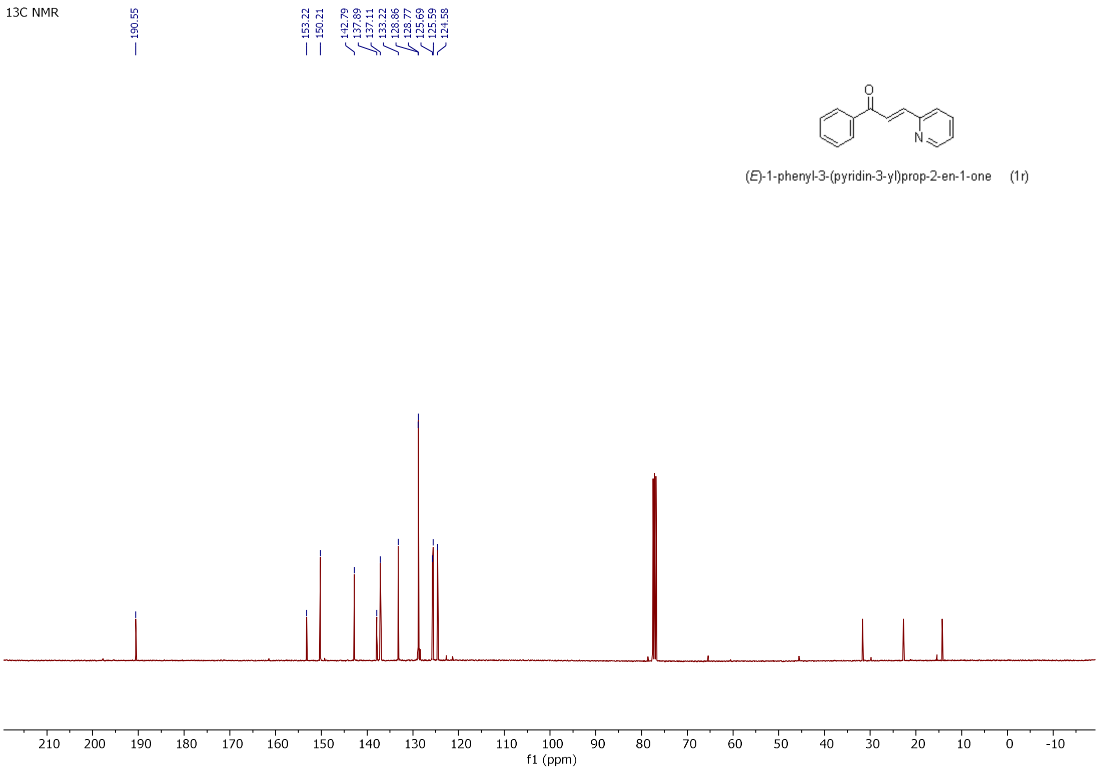

Supplementary Figure 40. NMR spectrum of **1r**.

^{1}H , ^{13}C and ^{19}F NMR spectra of γ,γ-dichloroketones (2a-o)

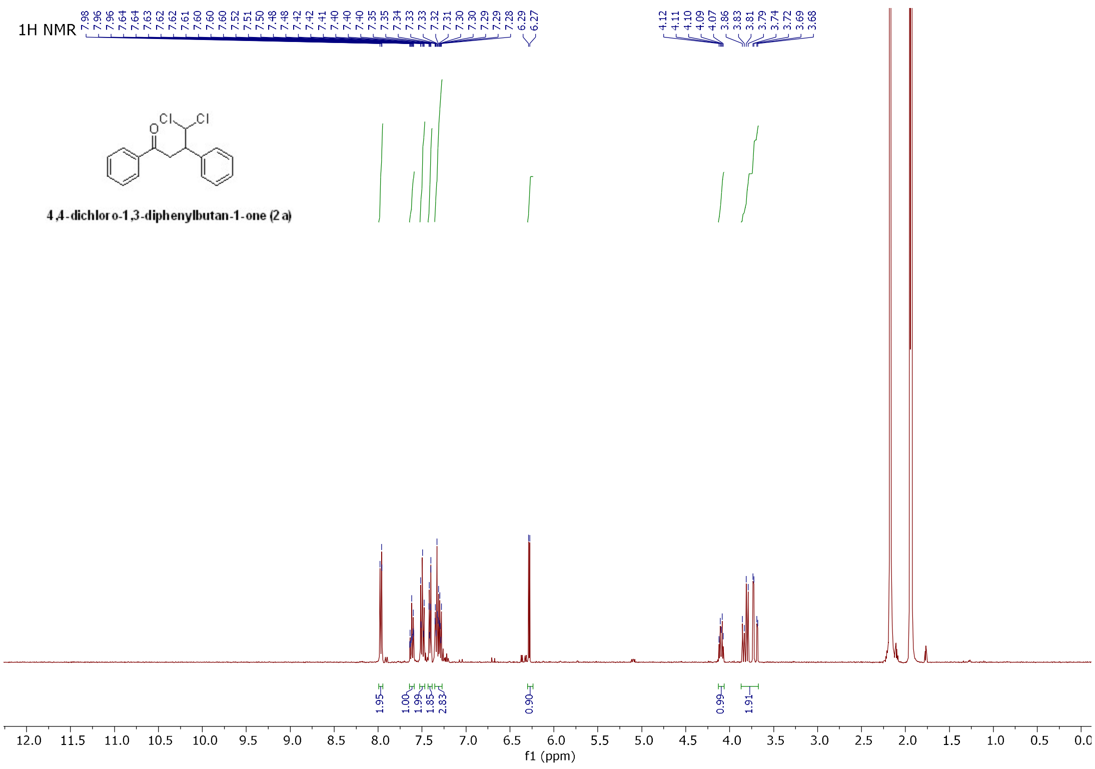

Supplementary Figure 41. NMR spectrum of 2a.

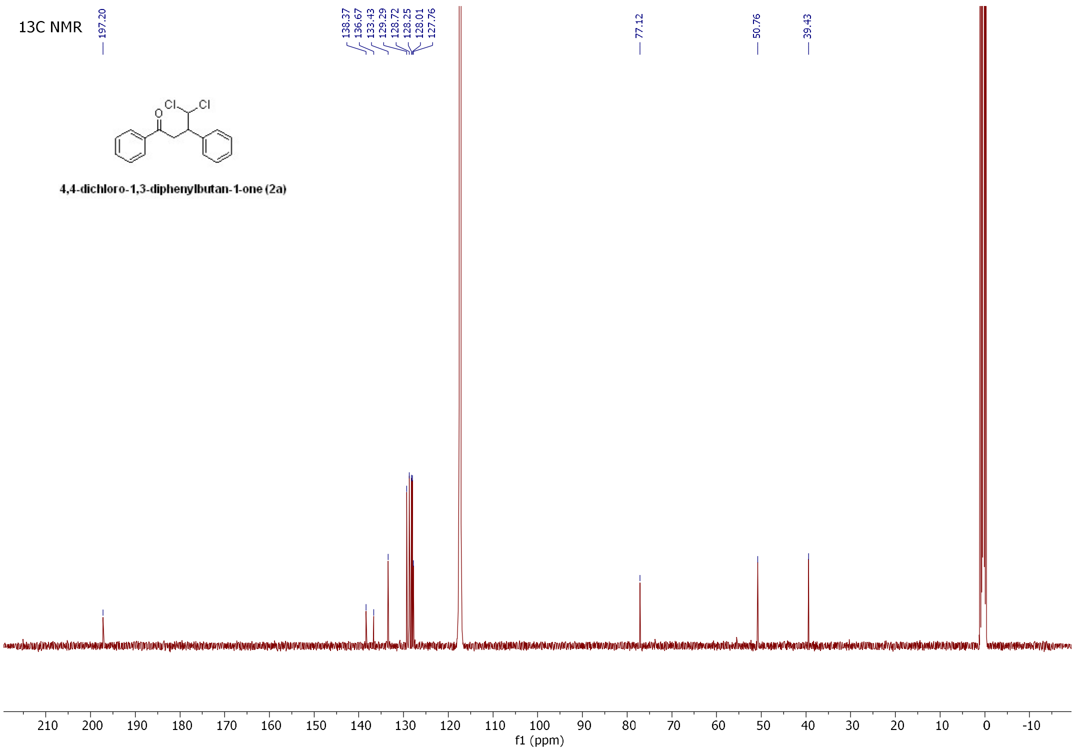

Supplementary Figure 42. NMR spectrum of 2a.

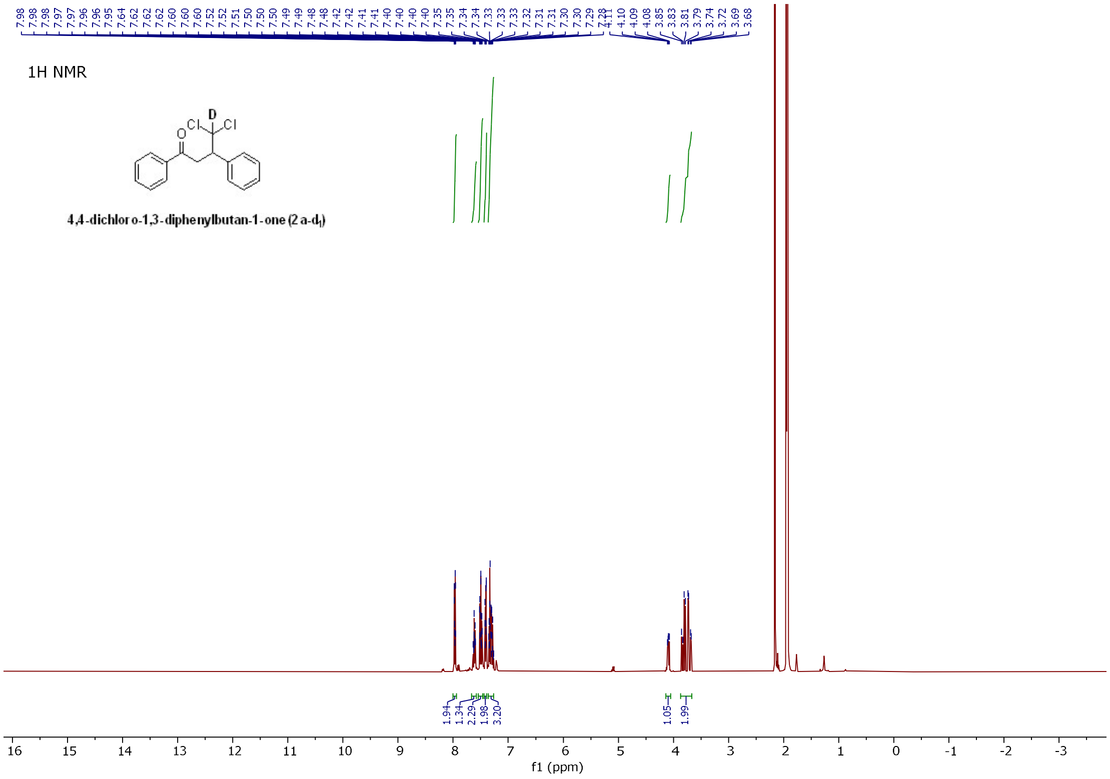

Supplementary Figure 43. NMR spectrum of 2a-d$_1$.

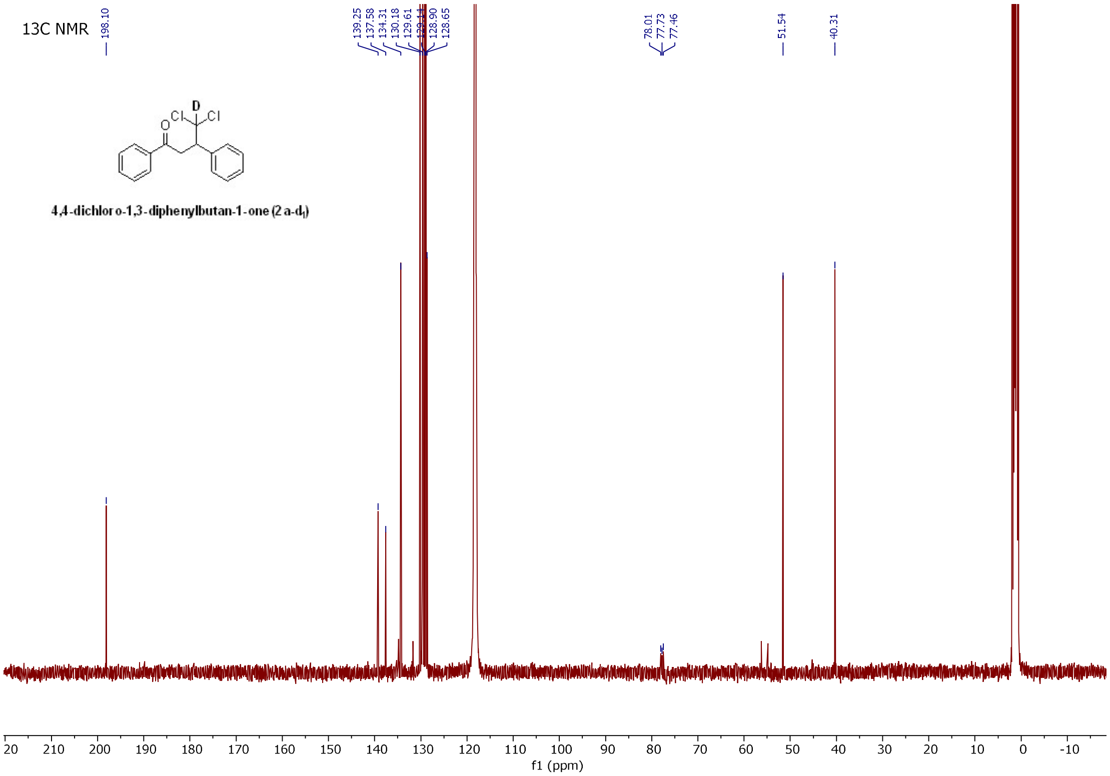

Supplementary Figure 44. NMR spectrum of 2a-d$_1$.

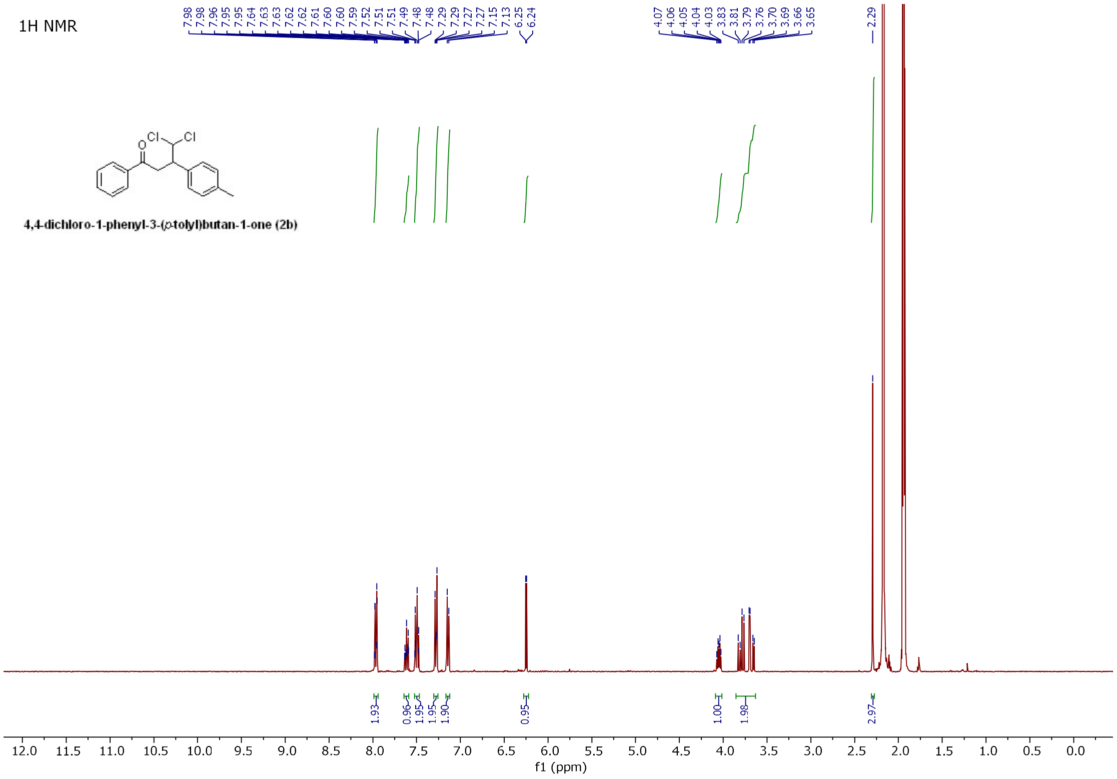

Supplementary Figure 45. NMR spectrum of 2b.

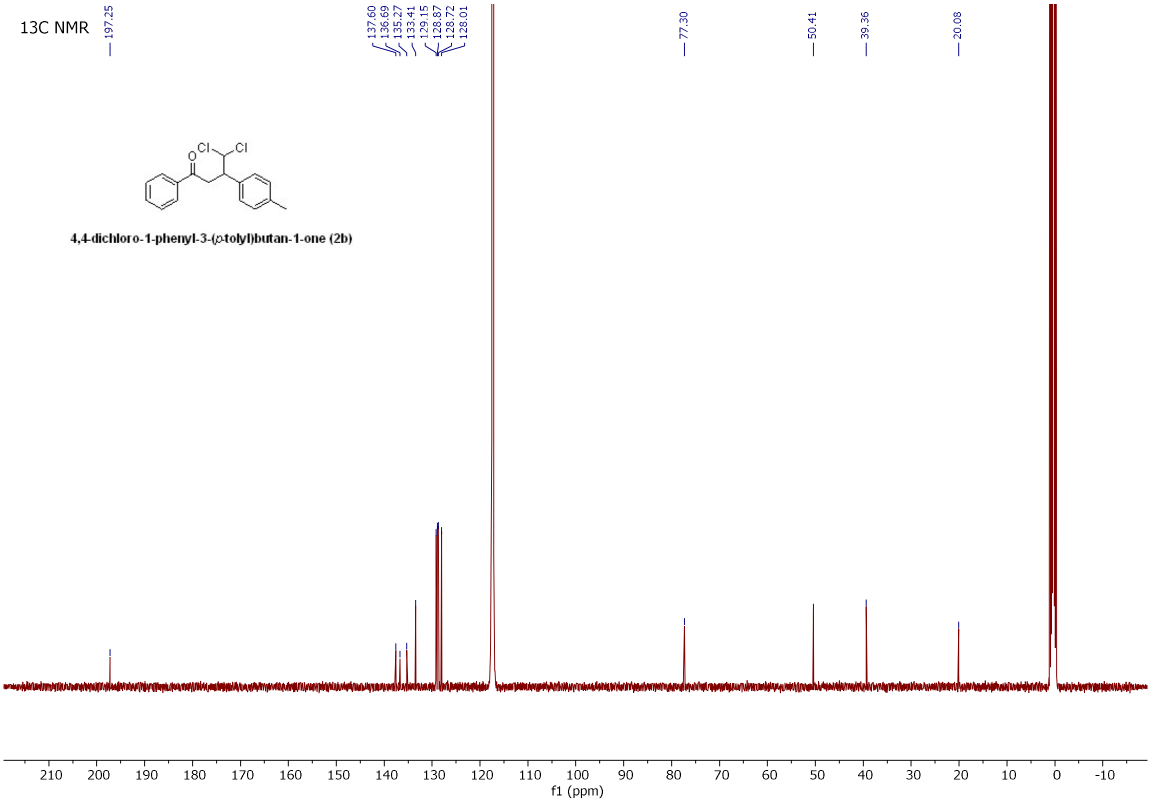

Supplementary Figure 46. NMR spectrum of 2b.

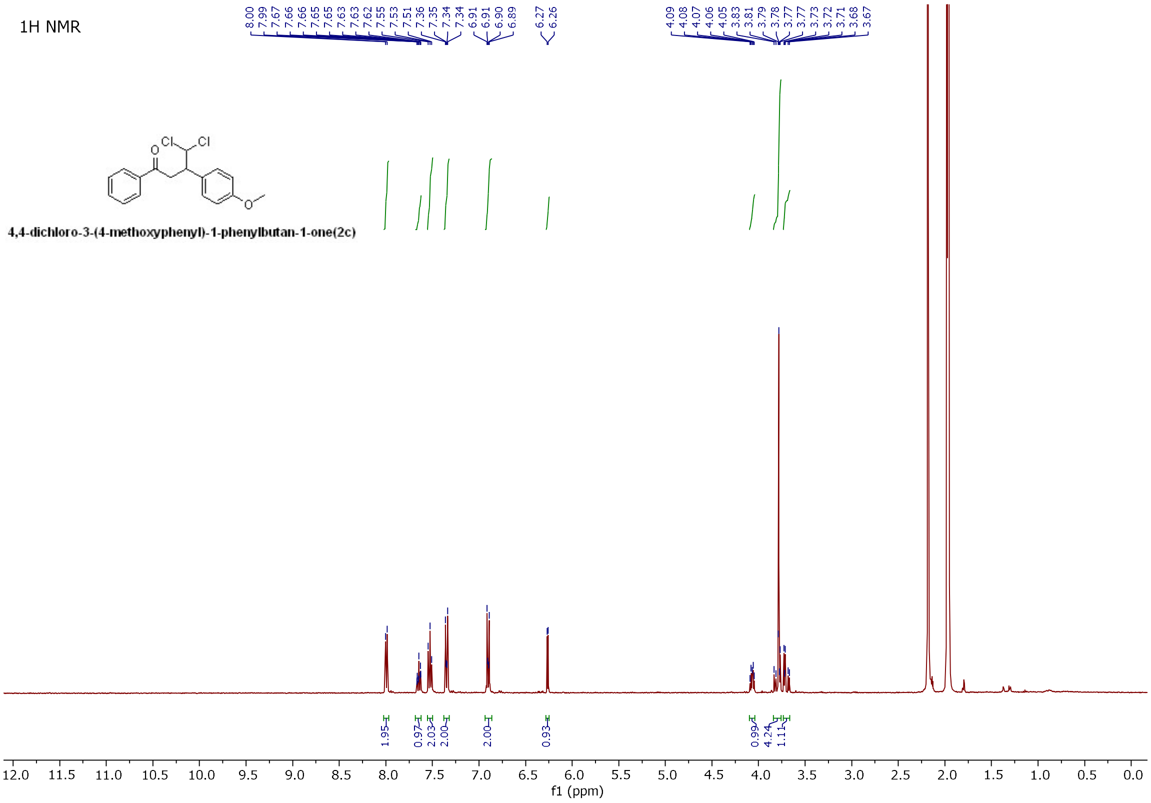

Supplementary Figure 47. NMR spectrum of 2c.

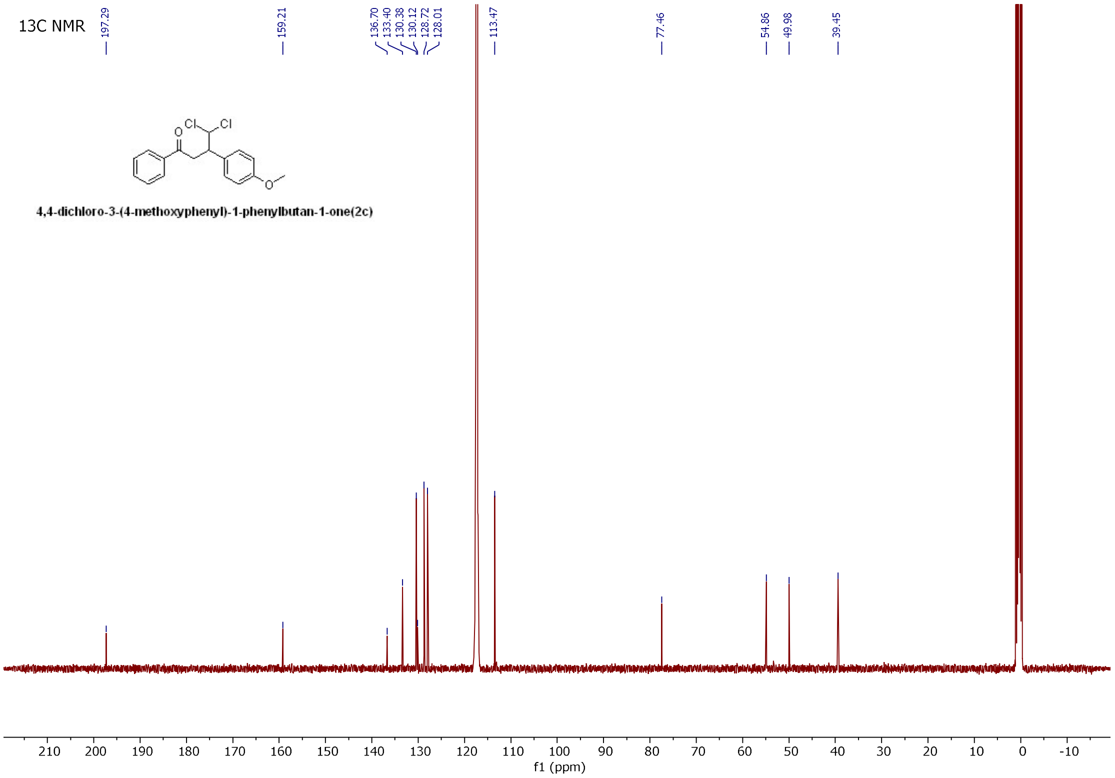

Supplementary Figure 48. NMR spectrum of 2c.

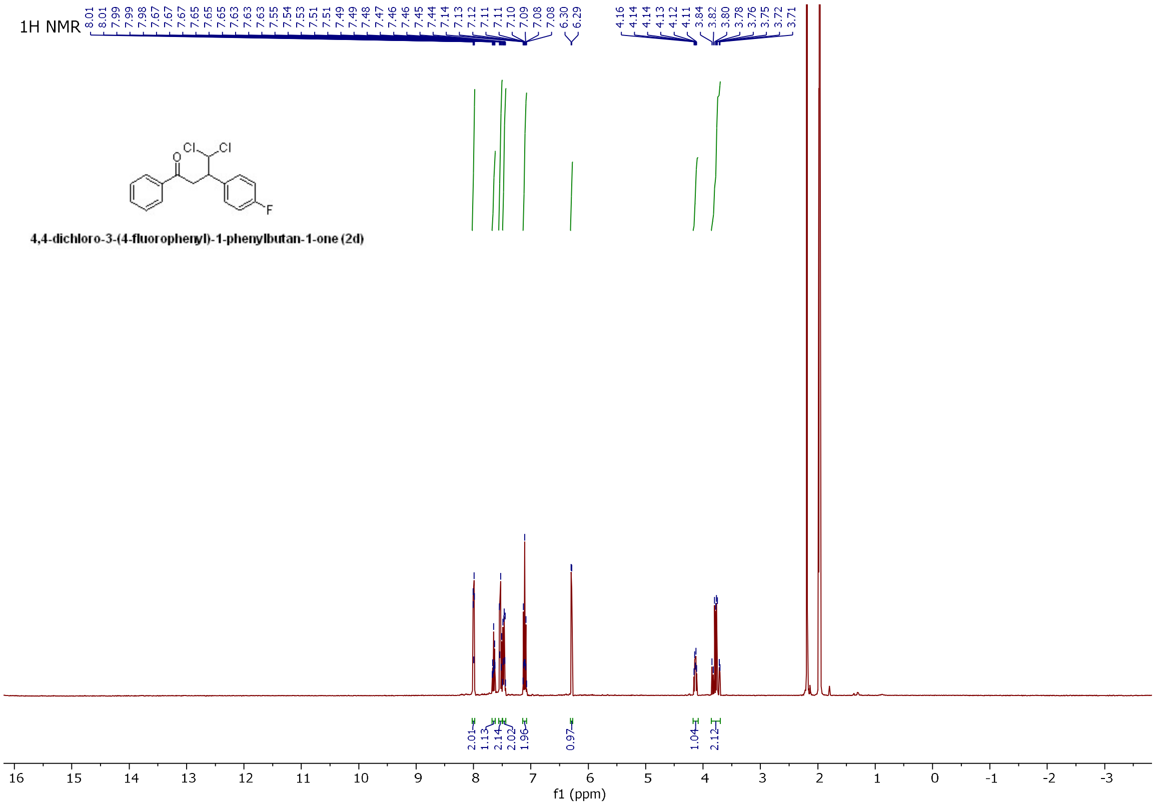

Supplementary Figure 49. NMR spectrum of 2d.

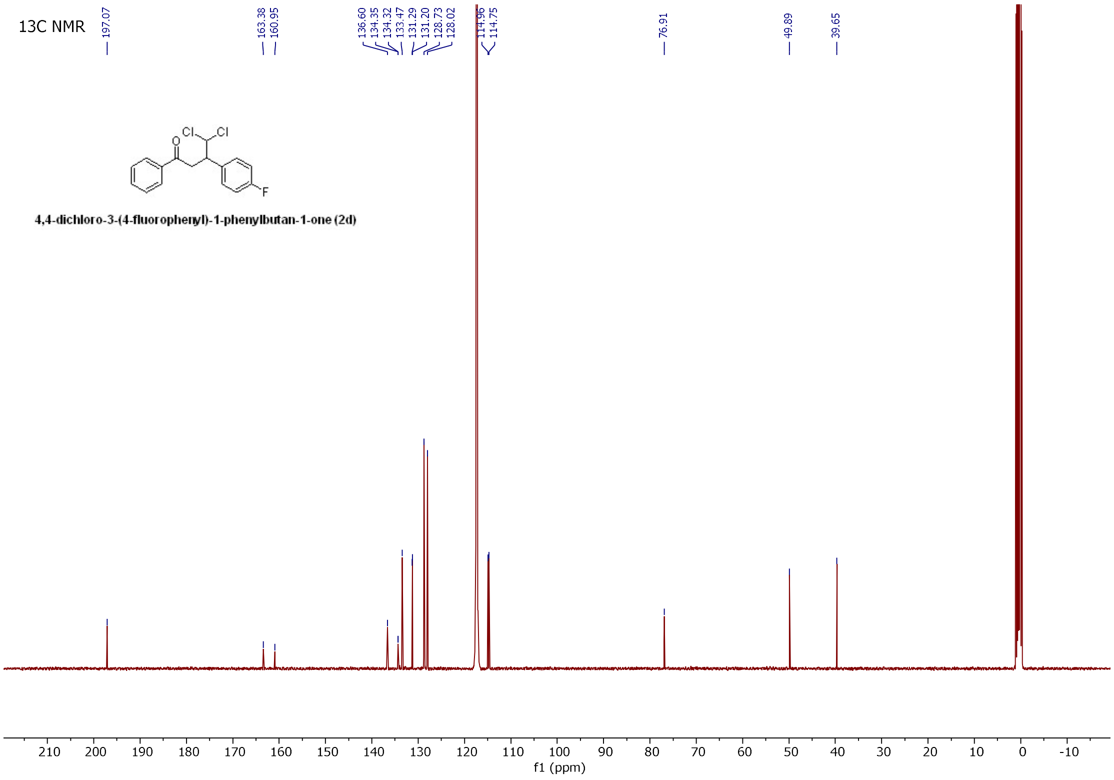

Supplementary Figure 50. NMR spectrum of 2d.

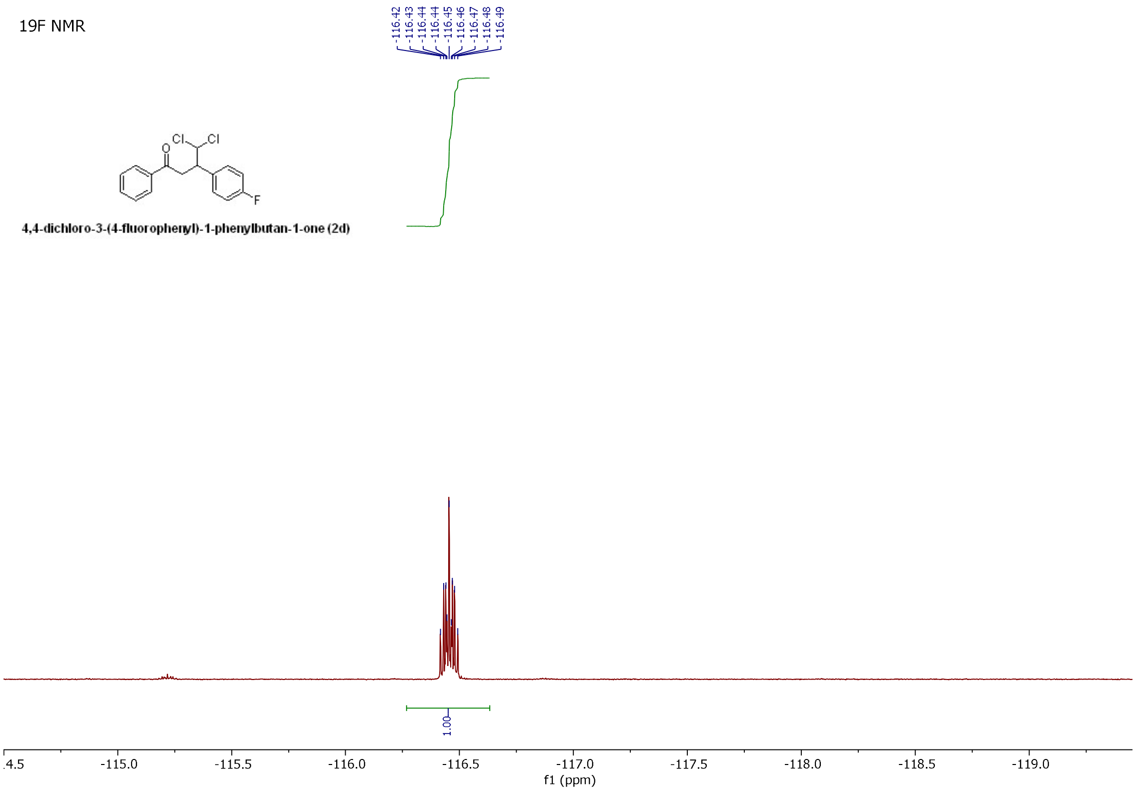

Supplementary Figure 51. NMR spectrum of 2d.

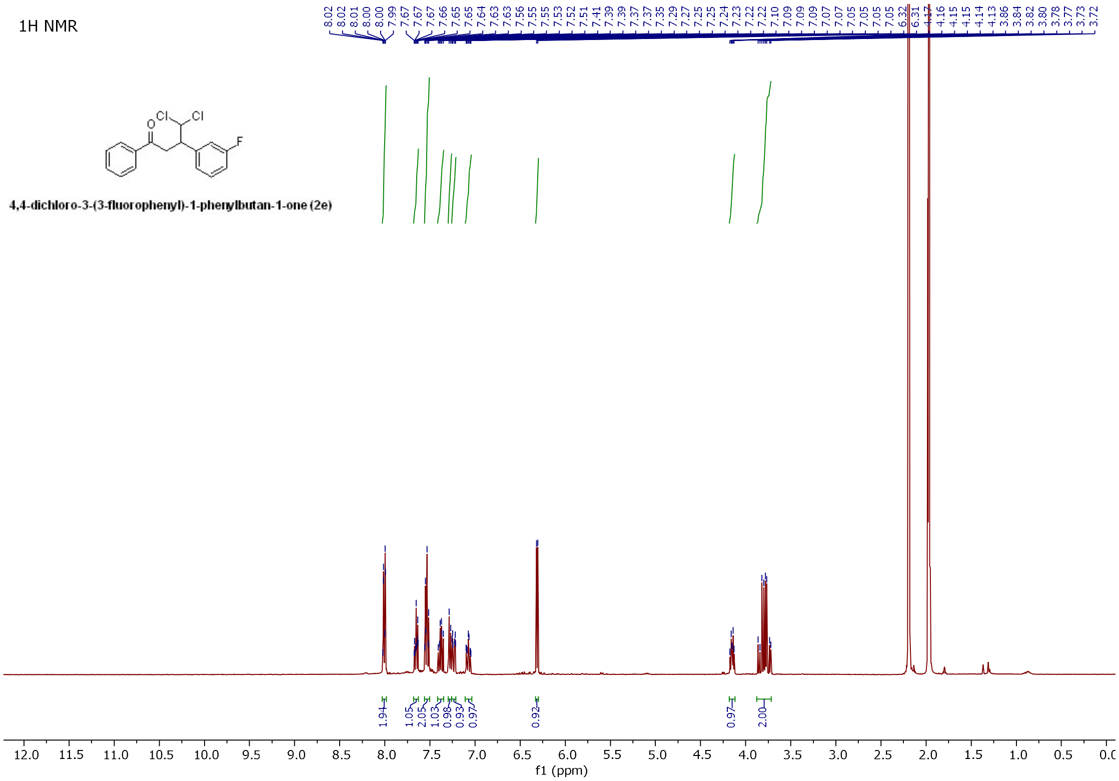

Supplementary Figure 52. NMR spectrum of 2e.

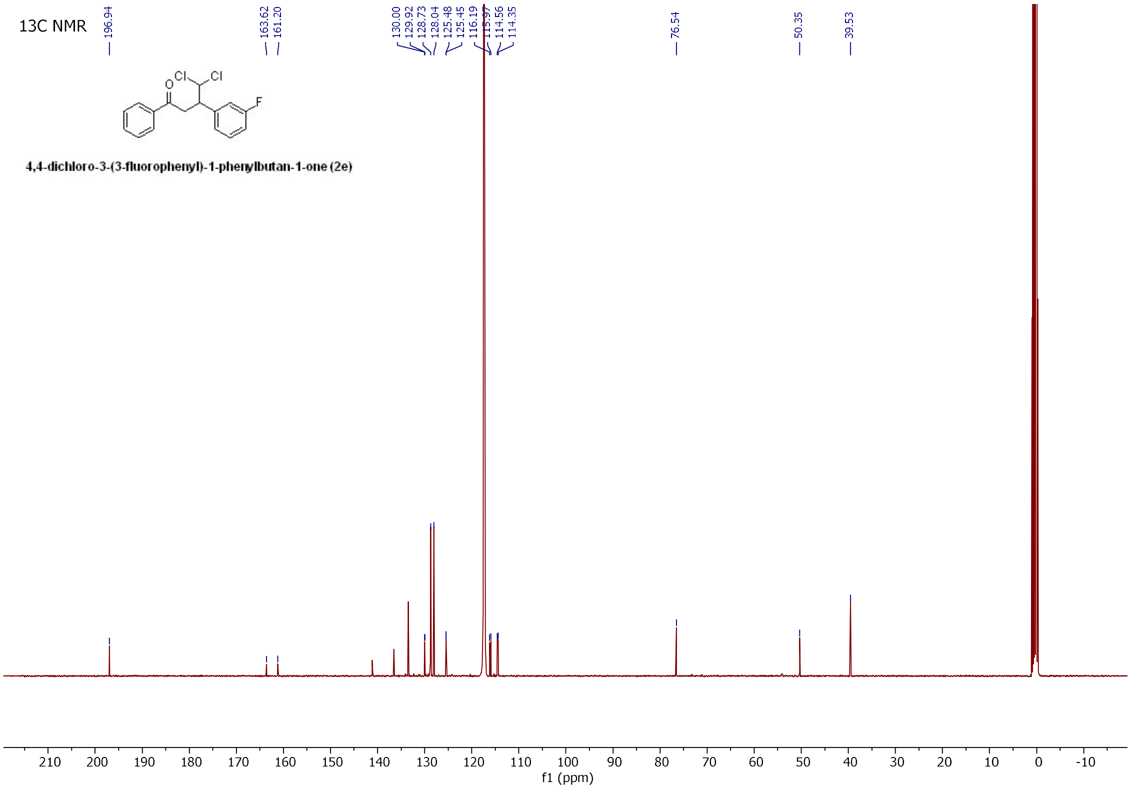

Supplementary Figure 53. NMR spectrum of 2e.

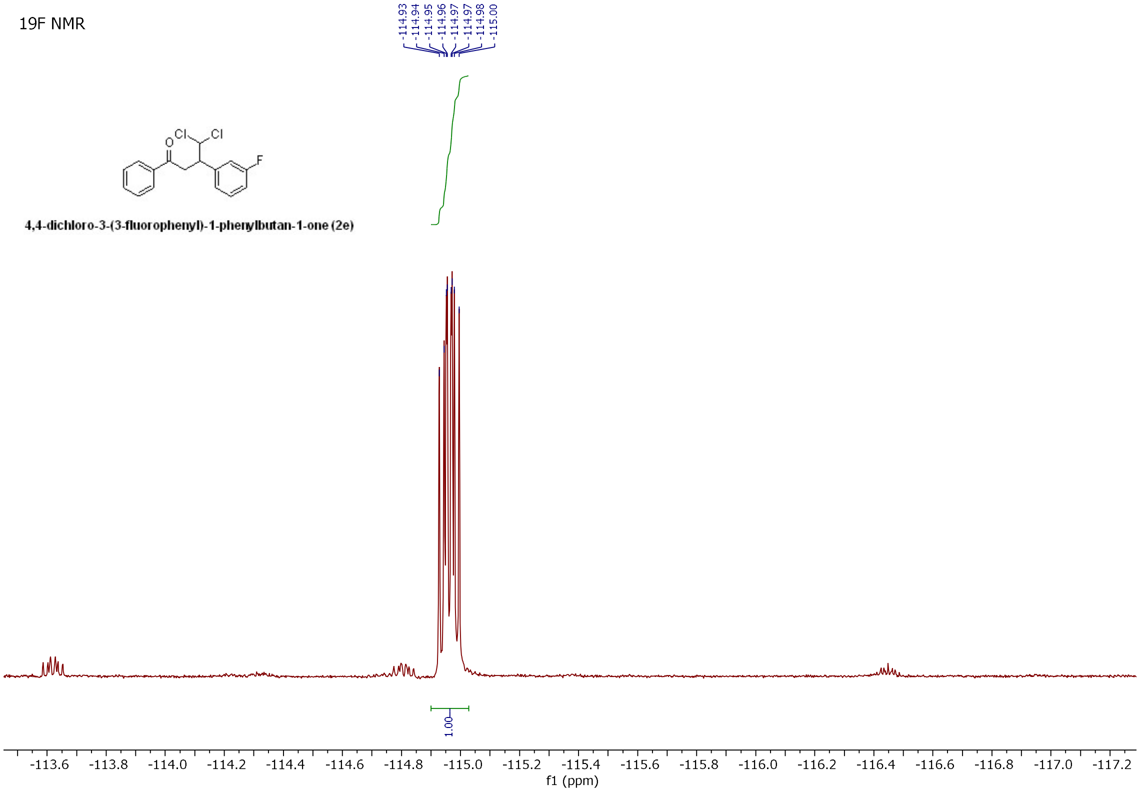

Supplementary Figure 54. NMR spectrum of 2e.

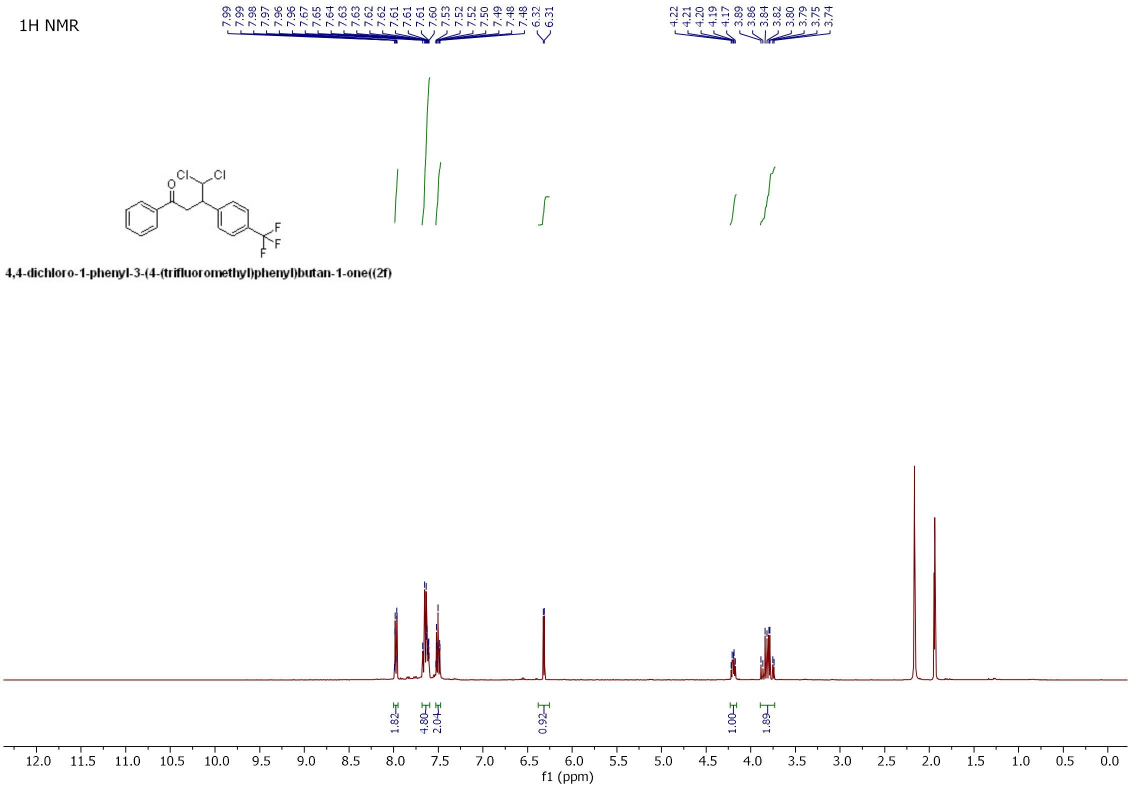

Supplementary Figure 55. NMR spectrum of 2f.

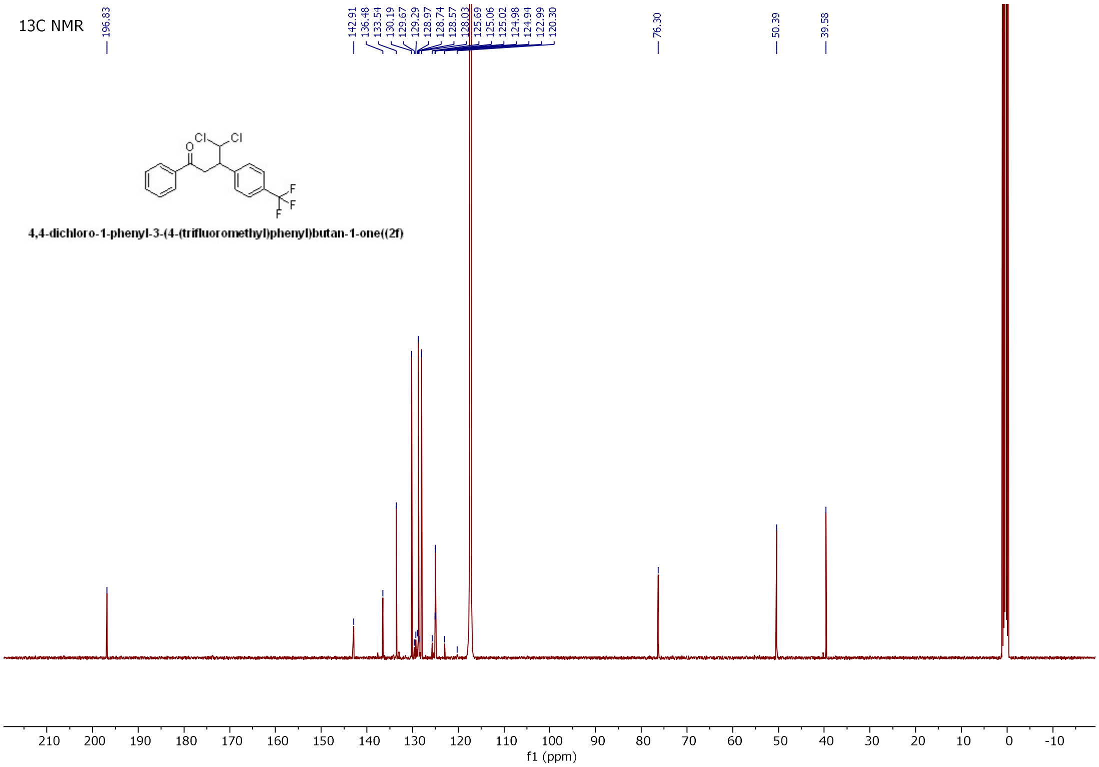

Supplementary Figure 56. NMR spectrum of 2f.

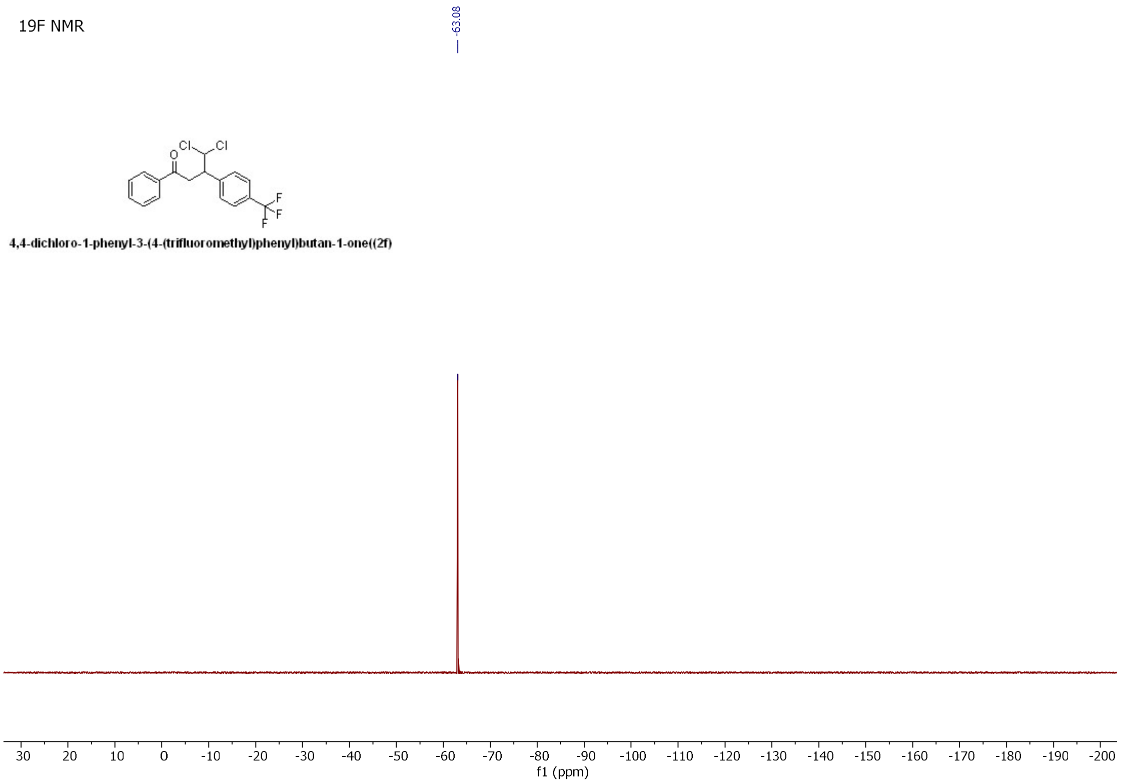

Supplementary Figure 57. NMR spectrum of 2f.

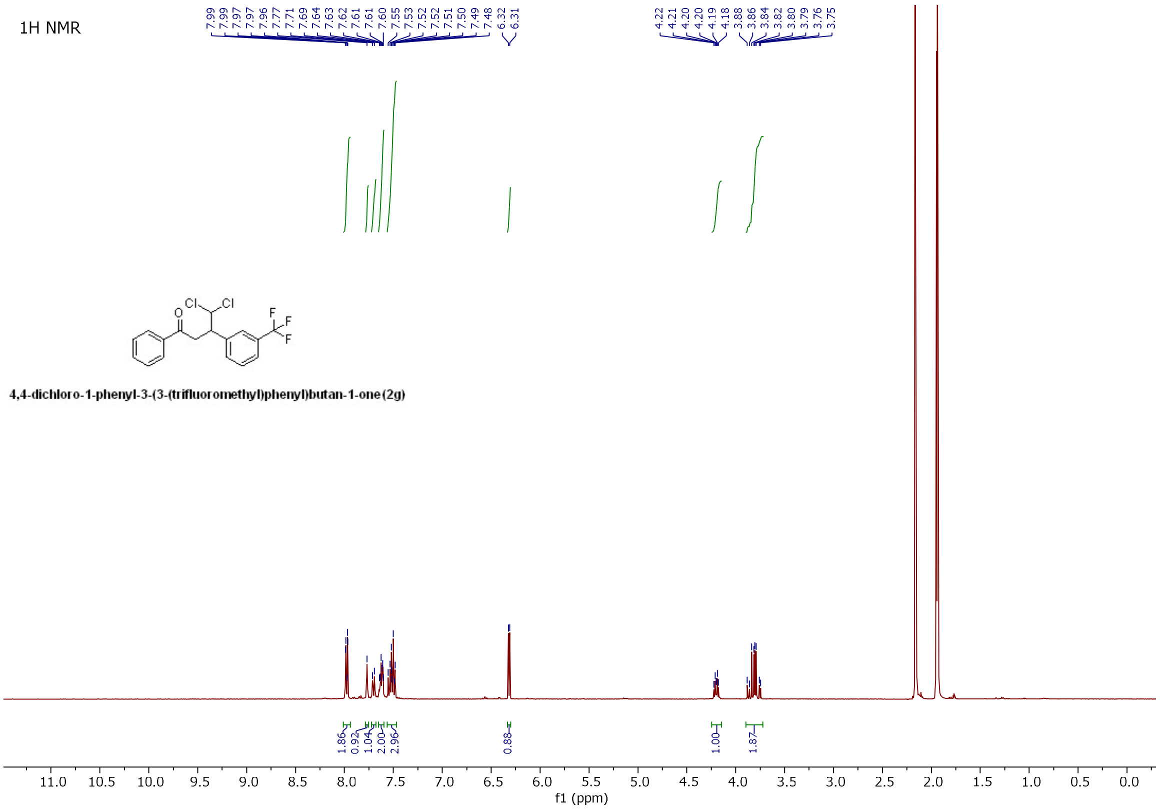

Supplementary Figure 58. NMR spectrum of 2g.

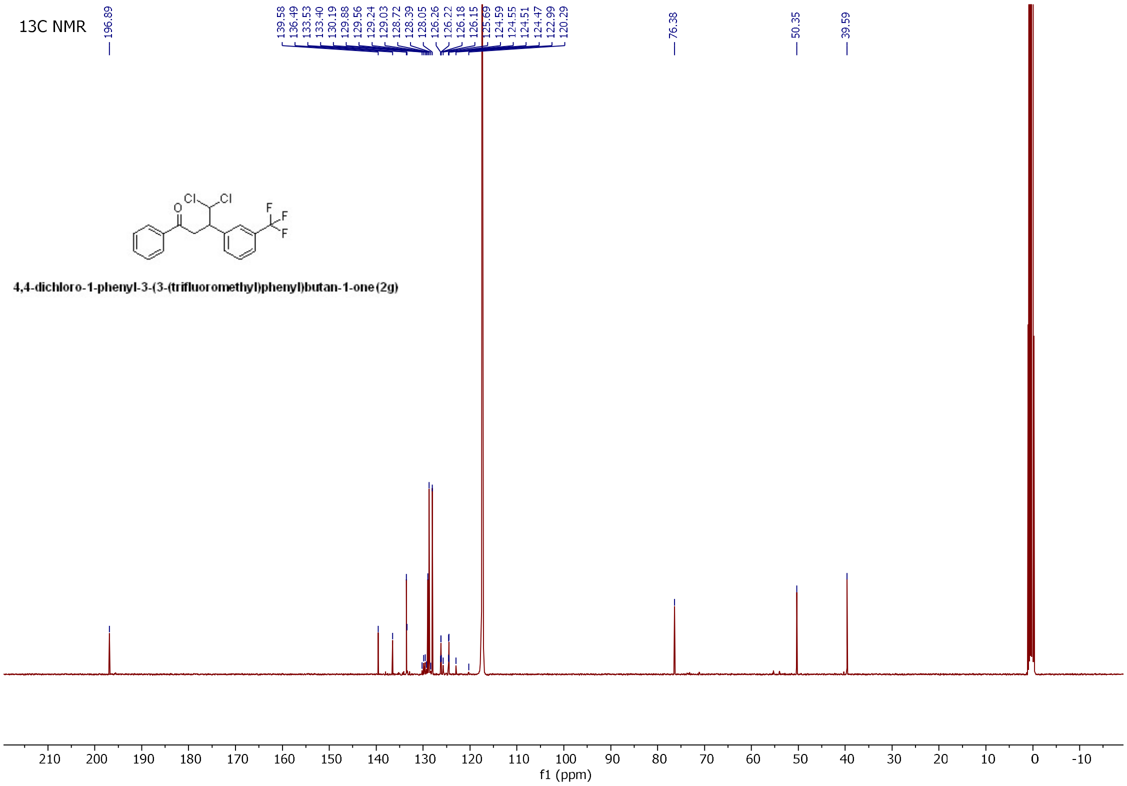

Supplementary Figure 59. NMR spectrum of **2g**.

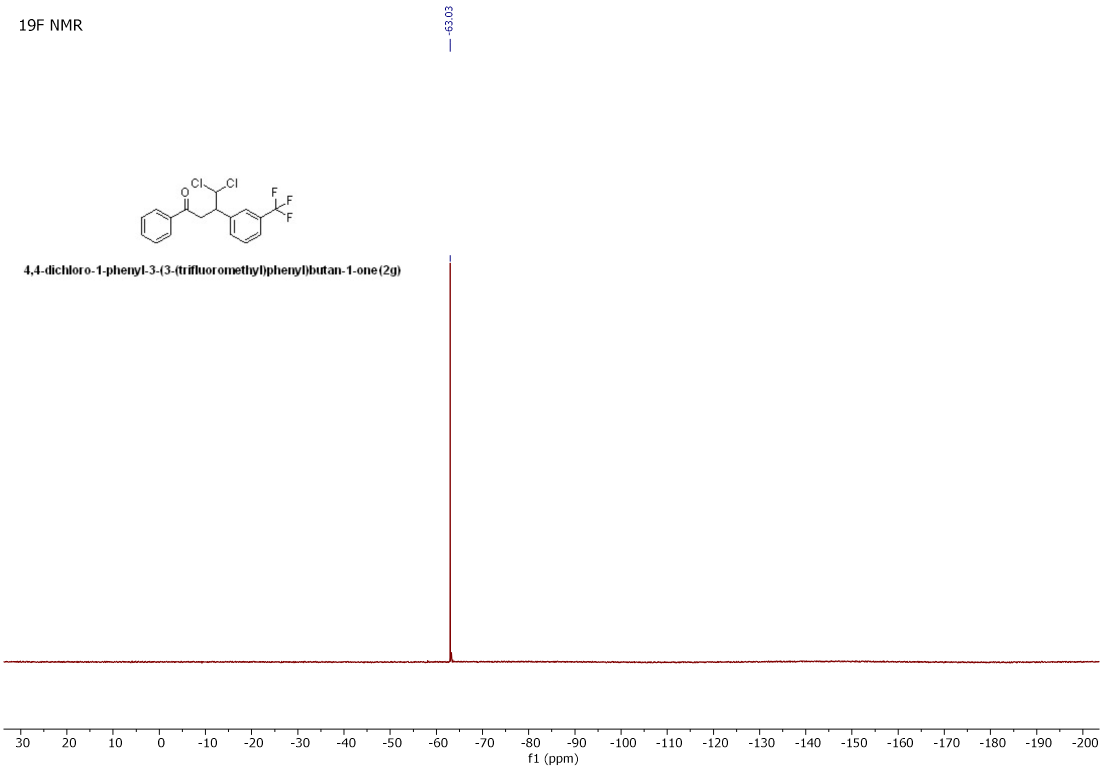

Supplementary Figure 60. NMR spectrum of **2g**.

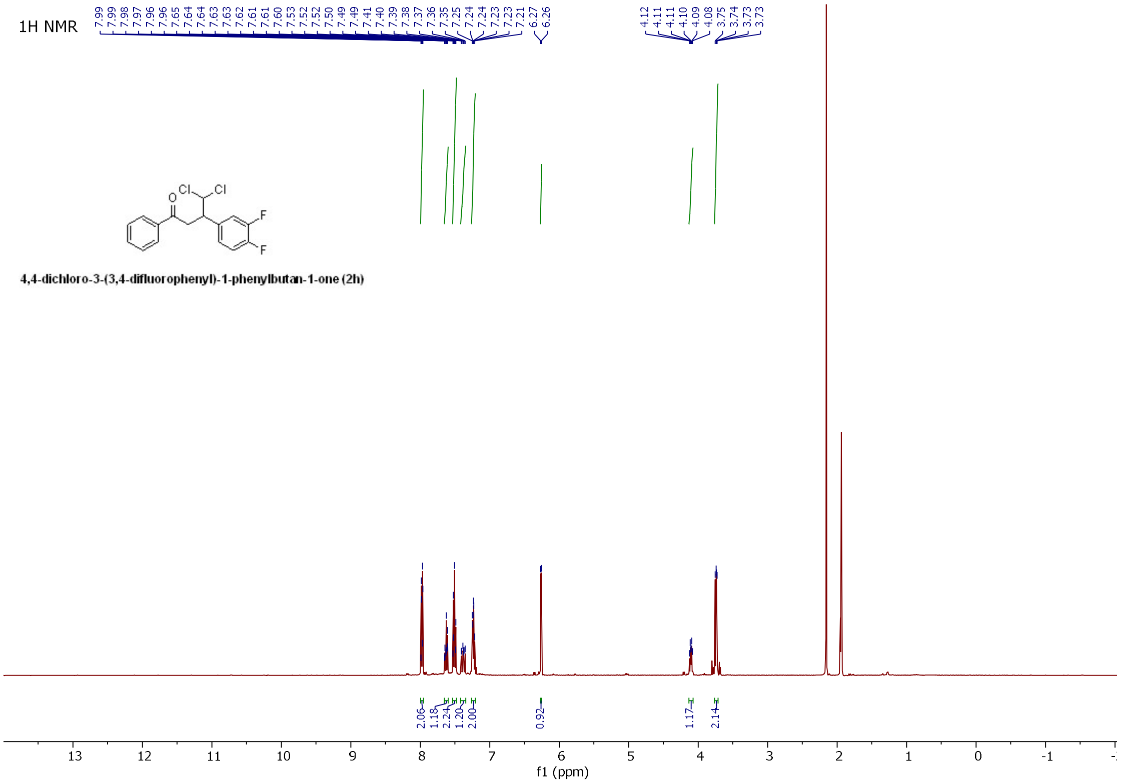

Supplementary Figure 61. NMR spectrum of 2h.

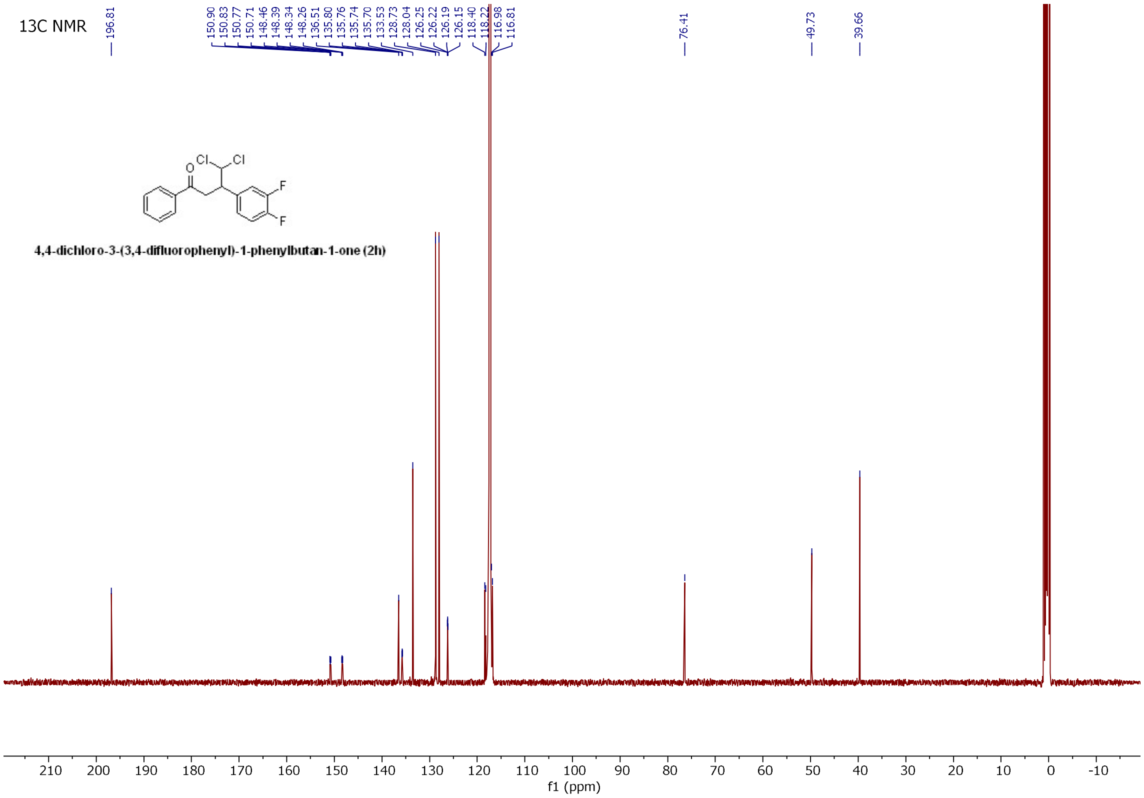

Supplementary Figure 62. NMR spectrum of 2h.

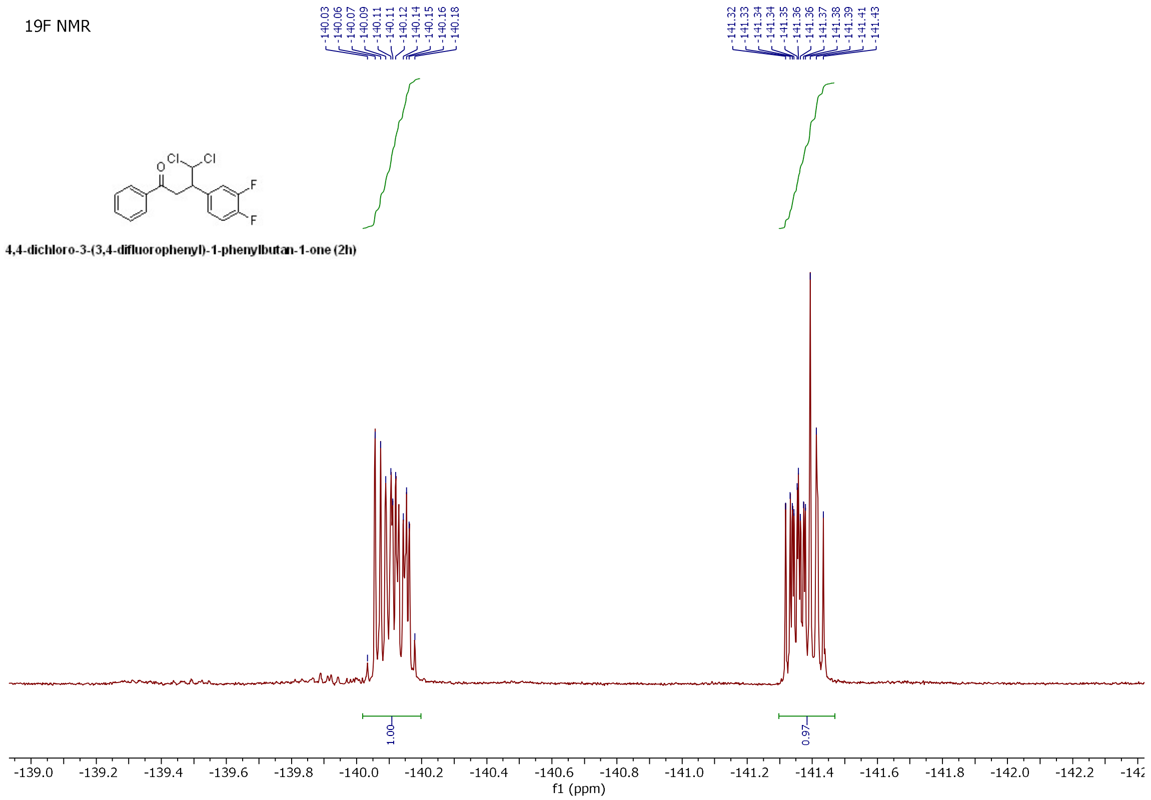

Supplementary Figure 63. NMR spectrum of 2h.

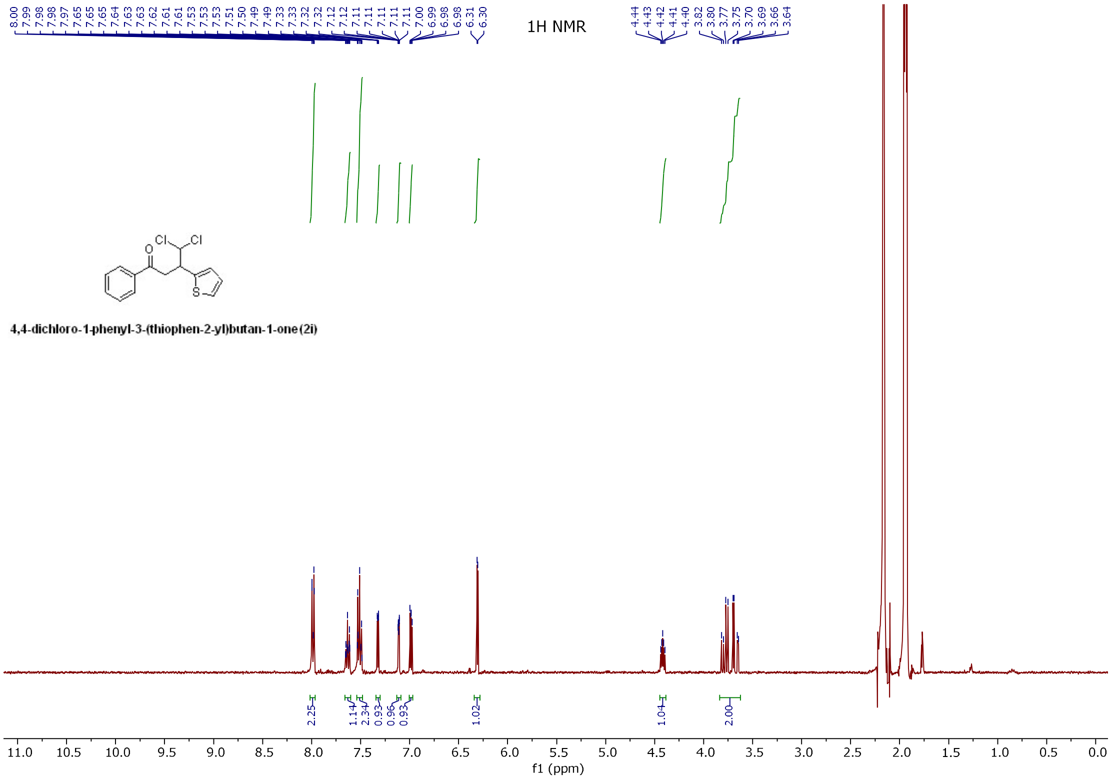

Supplementary Figure 64. NMR spectrum of 2i.

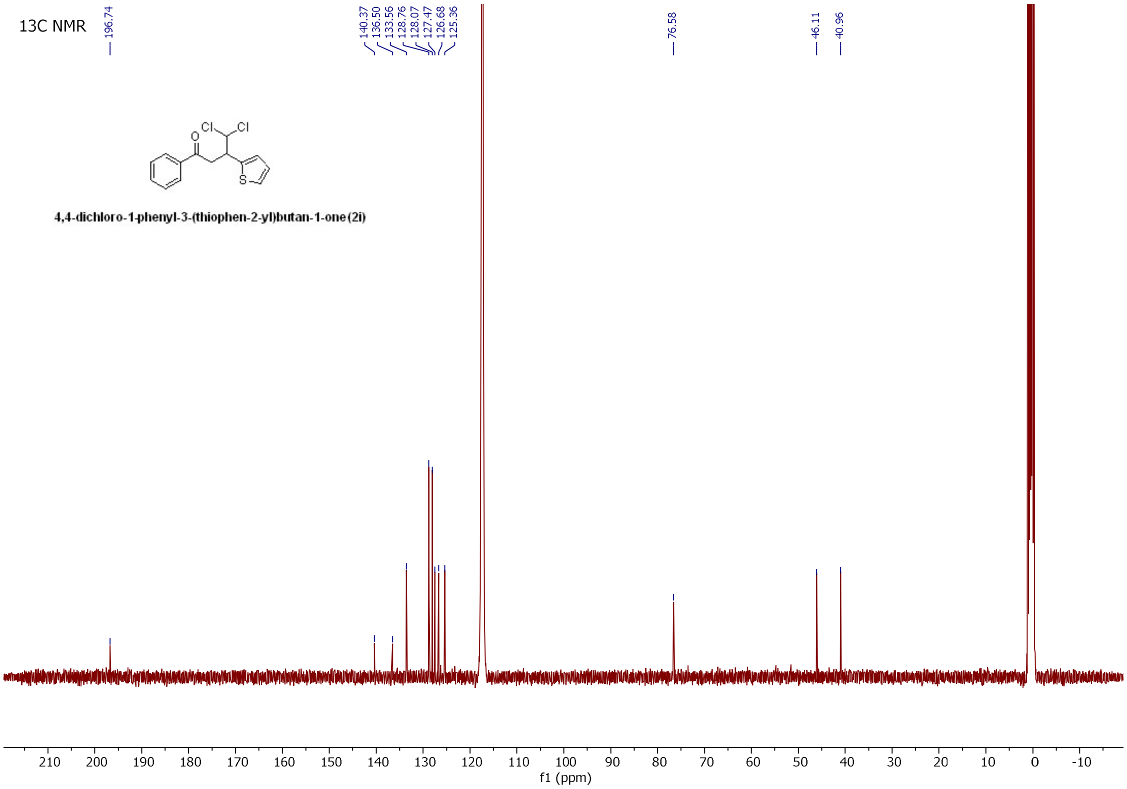

Supplementary Figure 65. NMR spectrum of 2i.

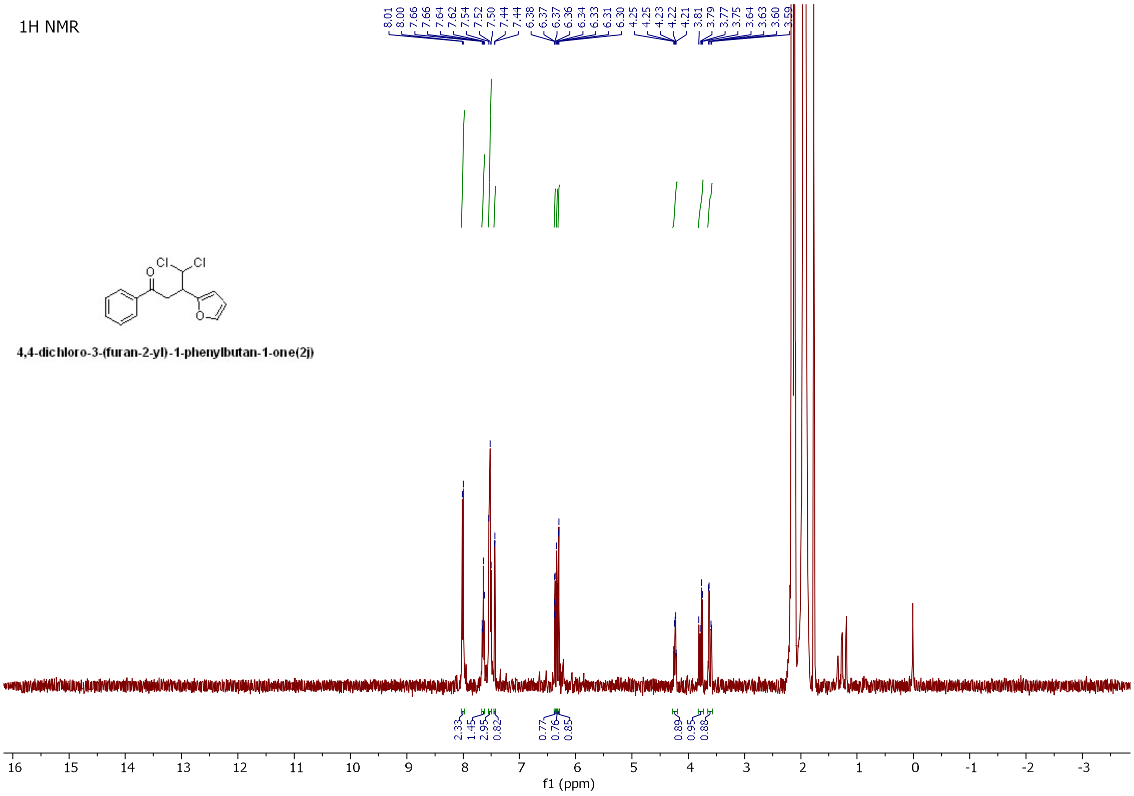

Supplementary Figure 66. NMR spectrum of 2j.

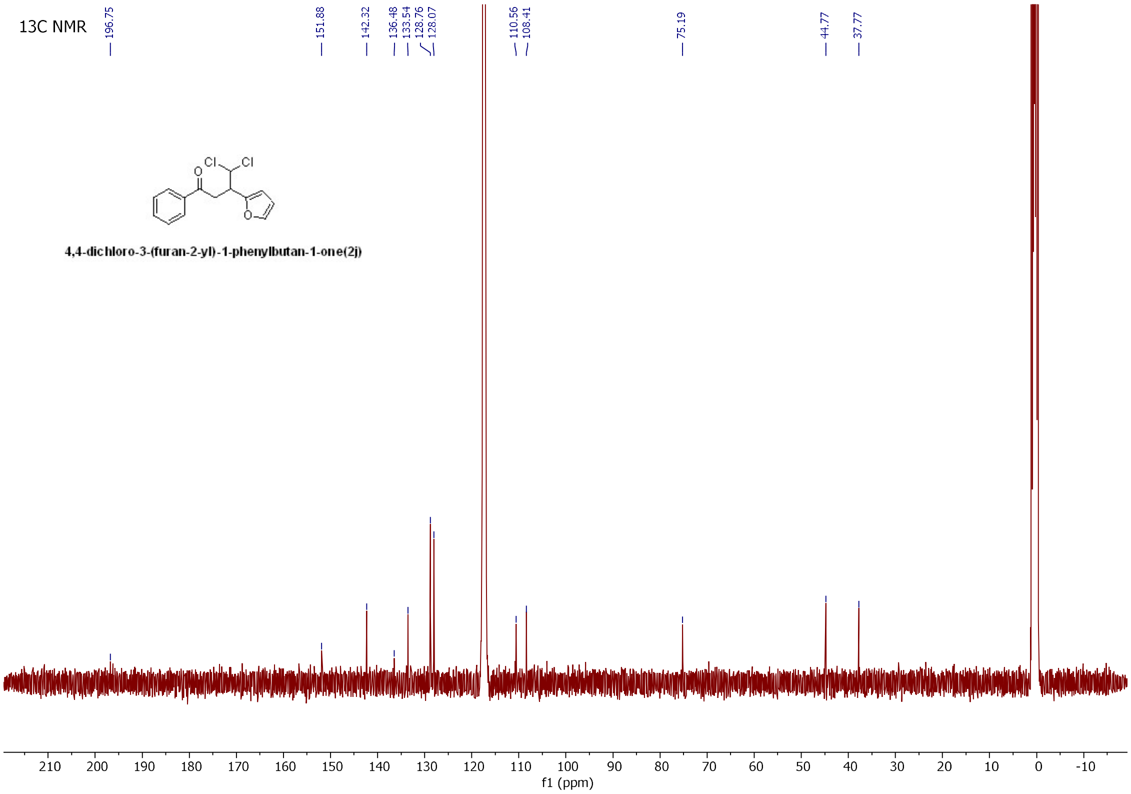

Supplementary Figure 67. NMR spectrum of 2j.

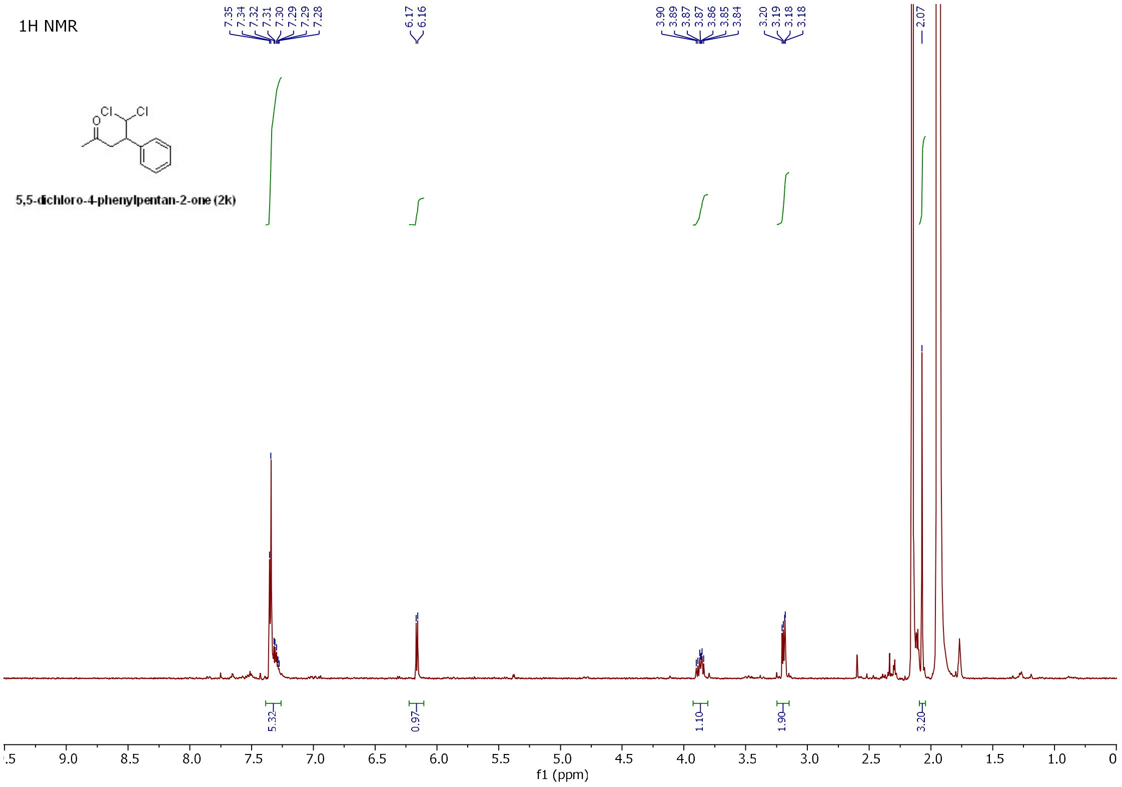

Supplementary Figure 68. NMR spectrum of 2k.

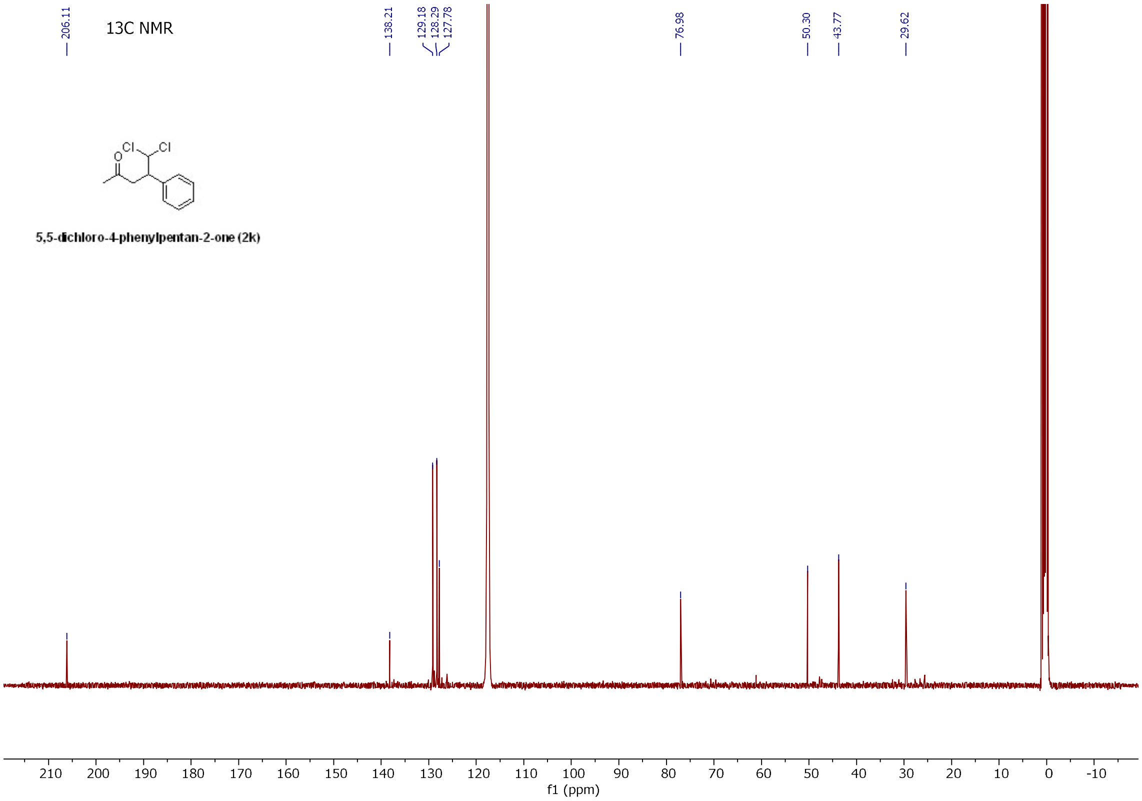

Supplementary Figure 69. NMR spectrum of 2k.

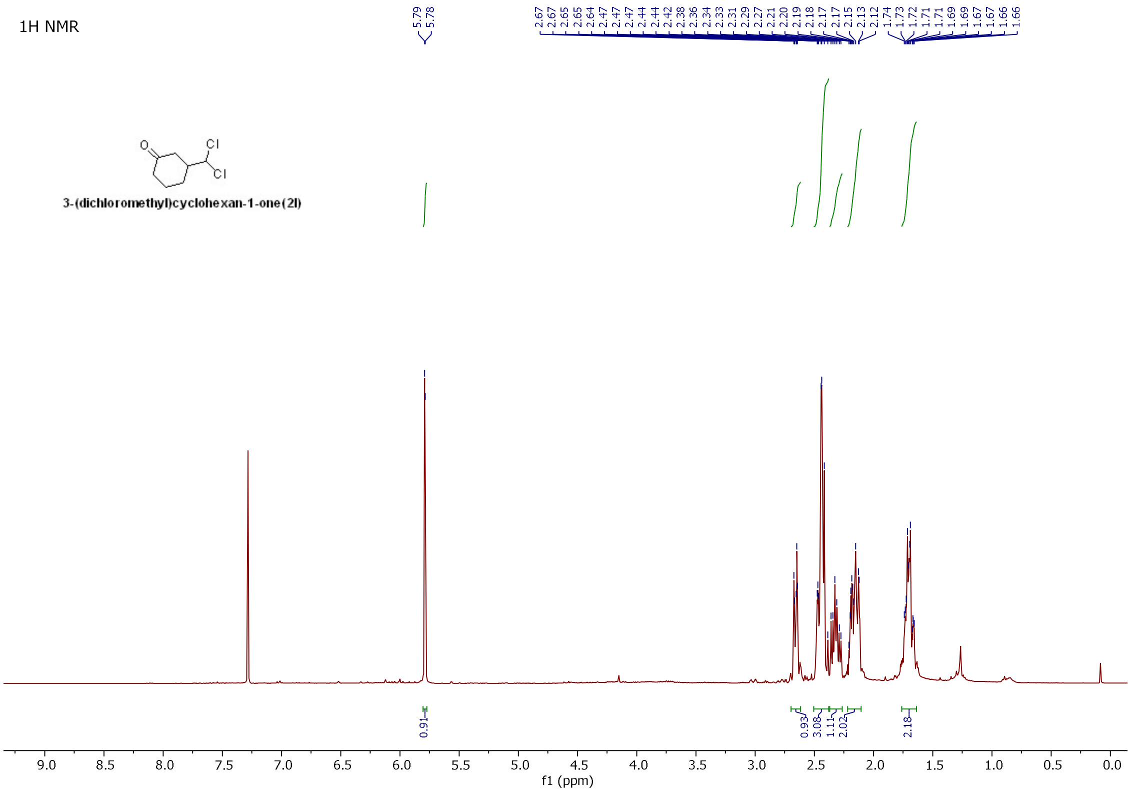

Supplementary Figure 70. NMR spectrum of 2l.

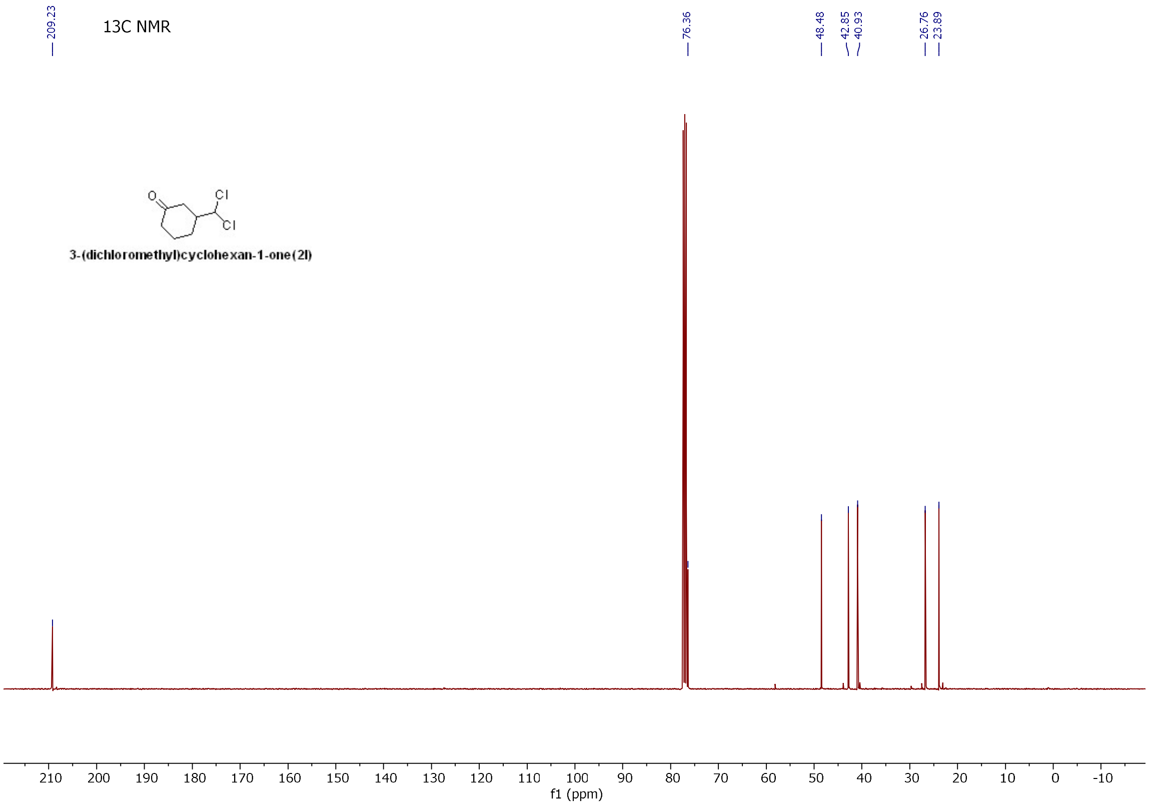

Supplementary Figure 71. NMR spectrum of 2l.

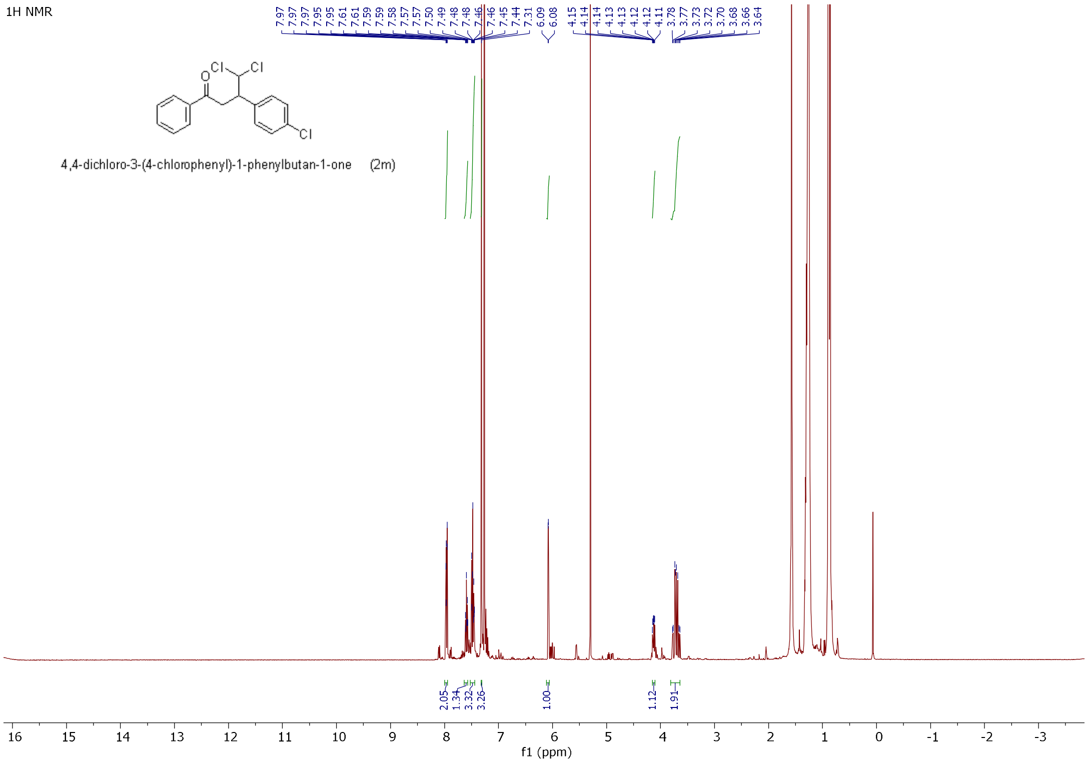

Supplementary Figure 72. NMR spectrum of 2m.

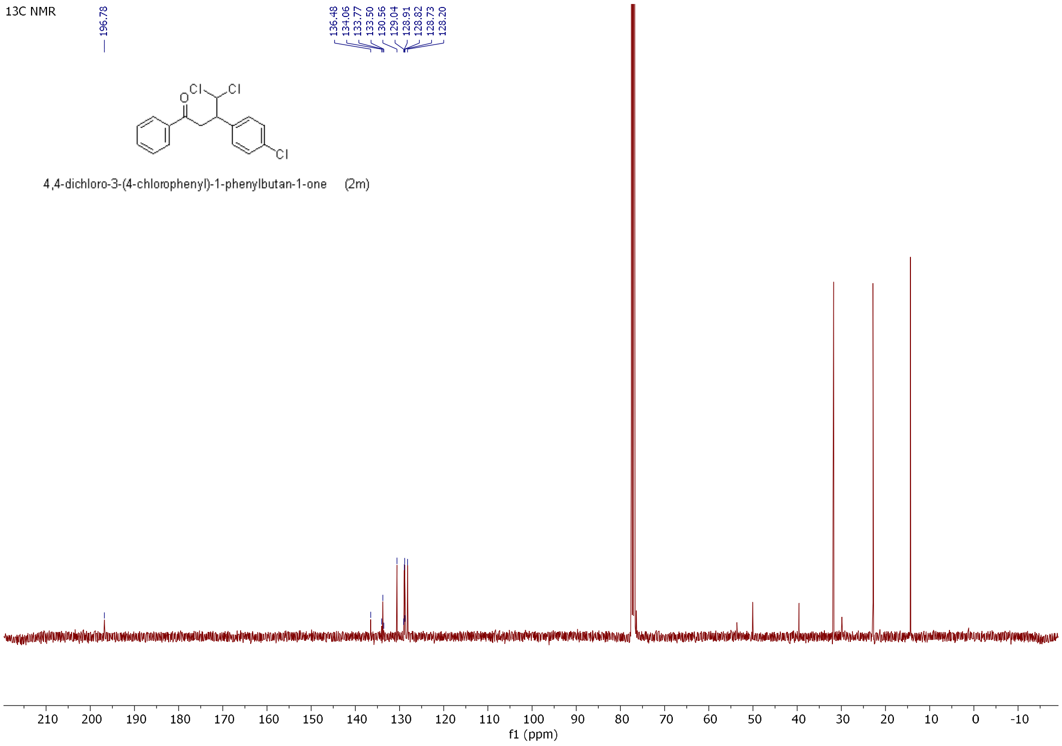

Supplementary Figure 73. NMR spectrum of **2m**.

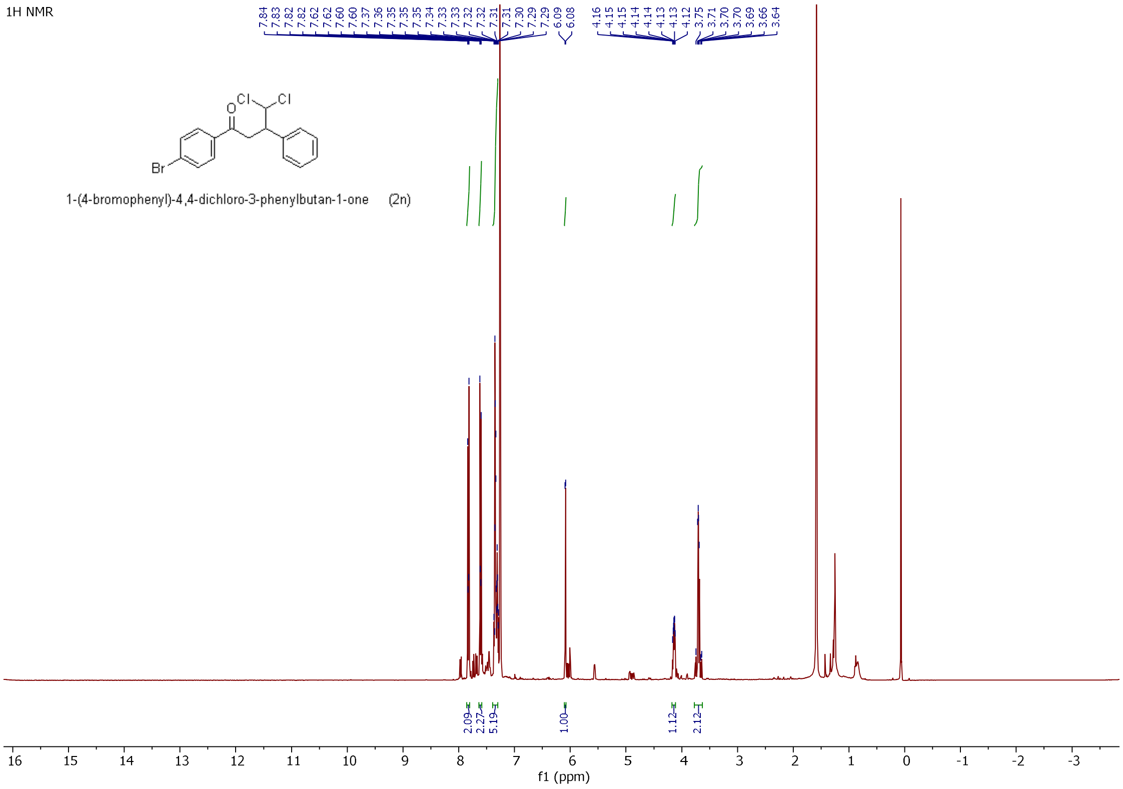

Supplementary Figure 74. NMR spectrum of **2n**.

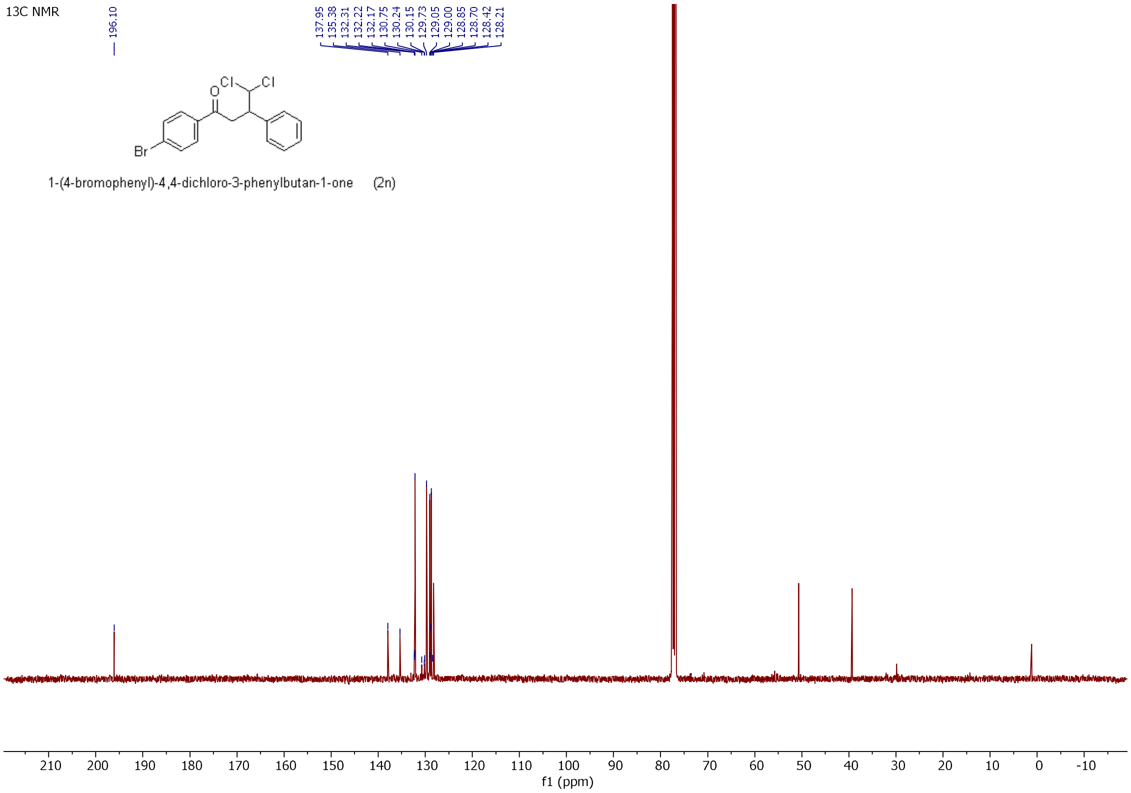

Supplementary Figure 75. NMR spectrum of 2n.

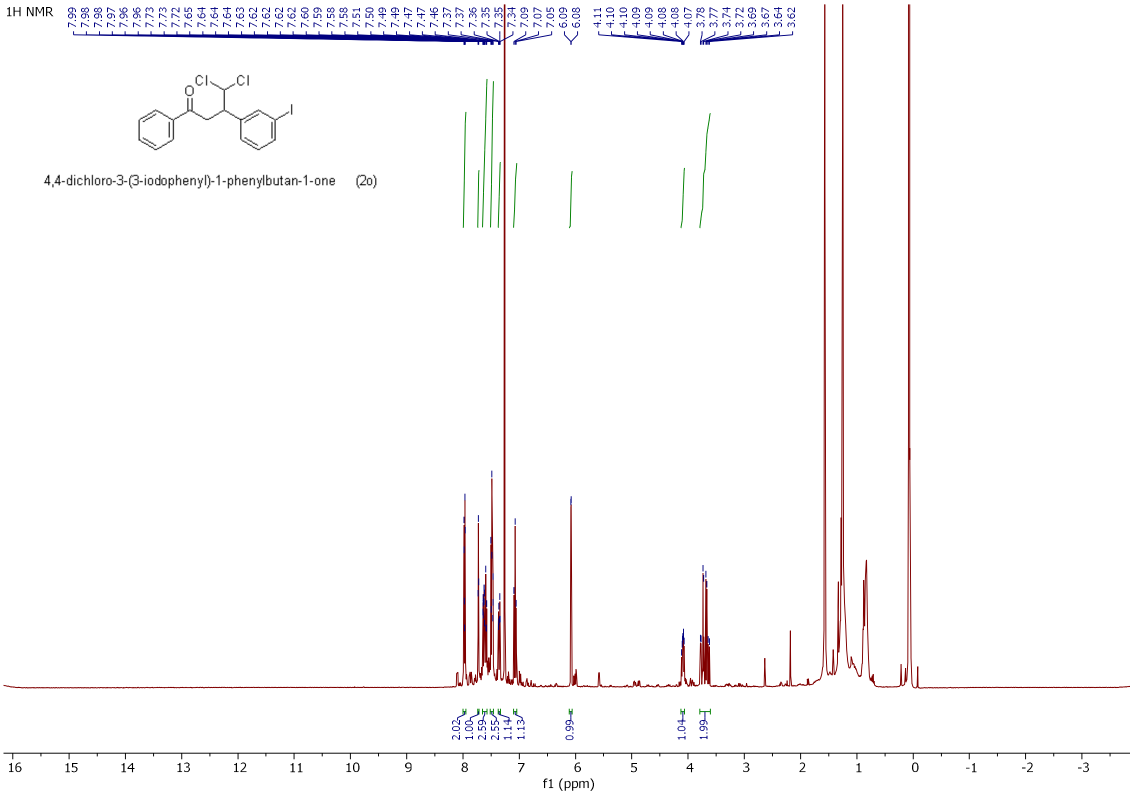

Supplementary Figure 76. NMR spectrum of 2o.

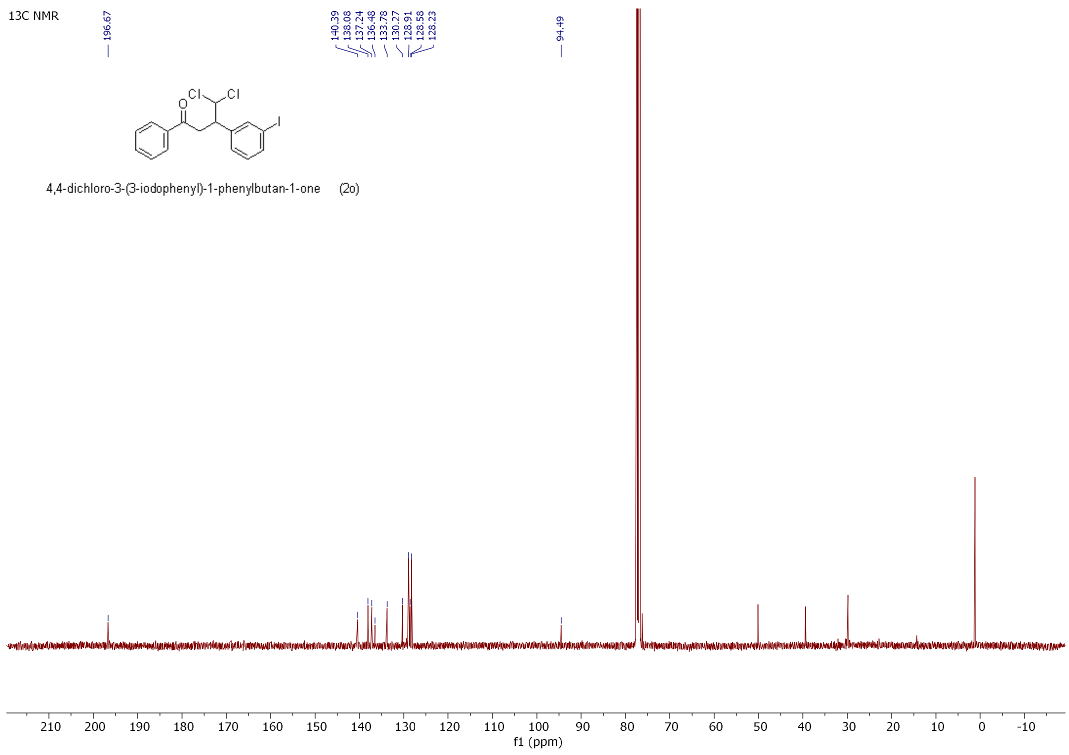

Supplementary Figure 77. NMR spectrum of 2o.

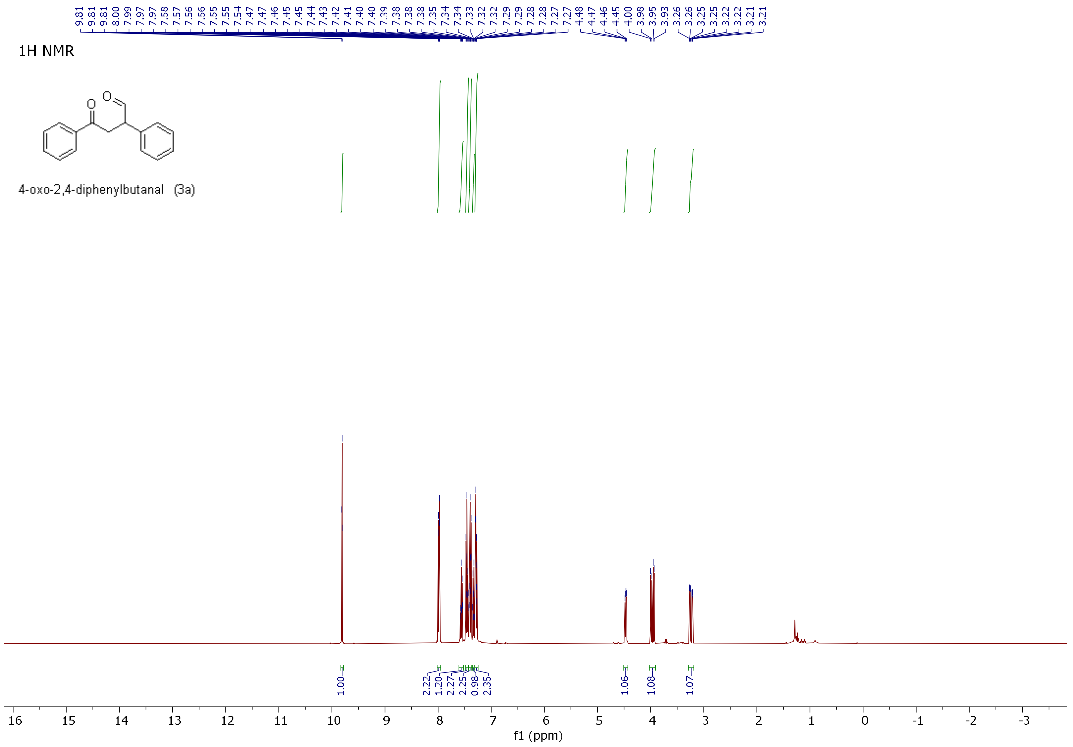

Supplementary Figure 78. NMR spectrum of 3a.

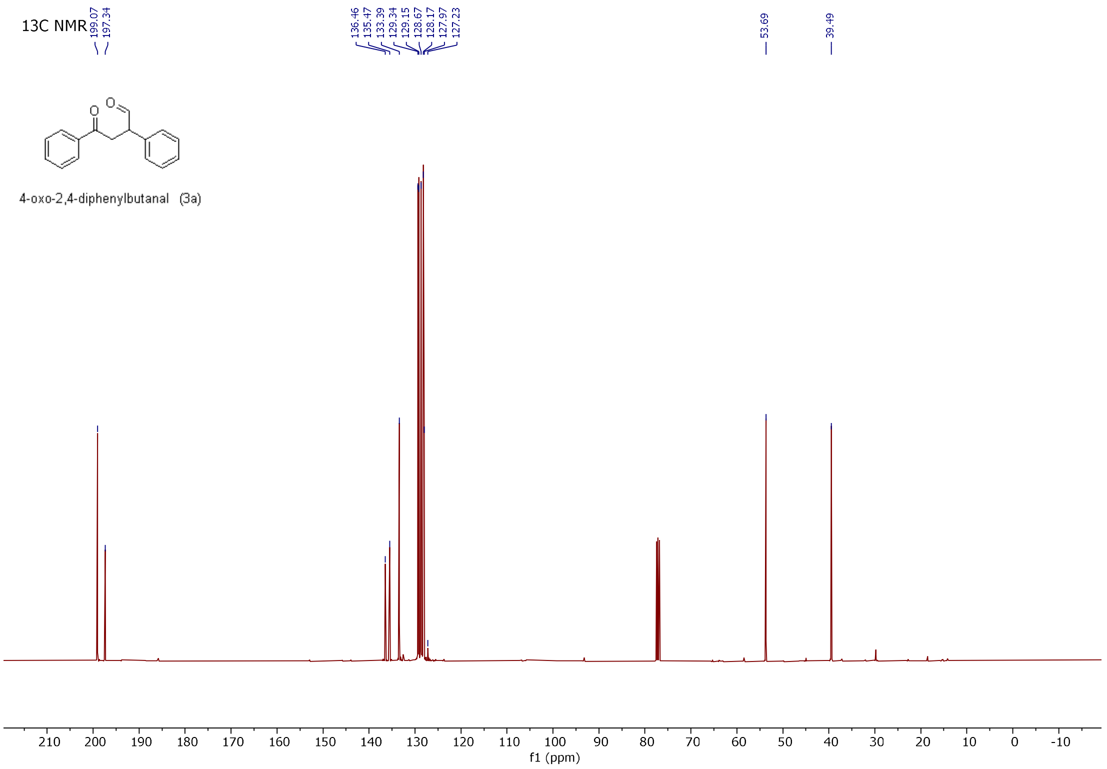

Supplementary Figure 79. NMR spectrum of 3a.

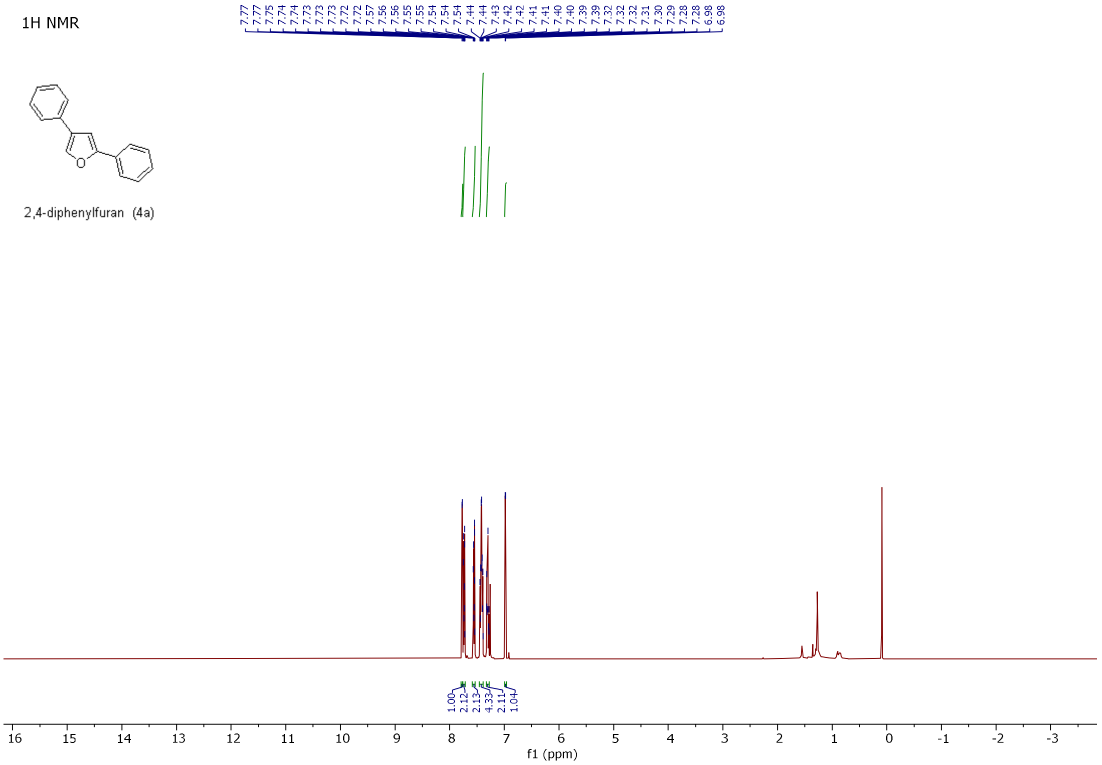

Supplementary Figure 80. NMR spectrum of 4a.

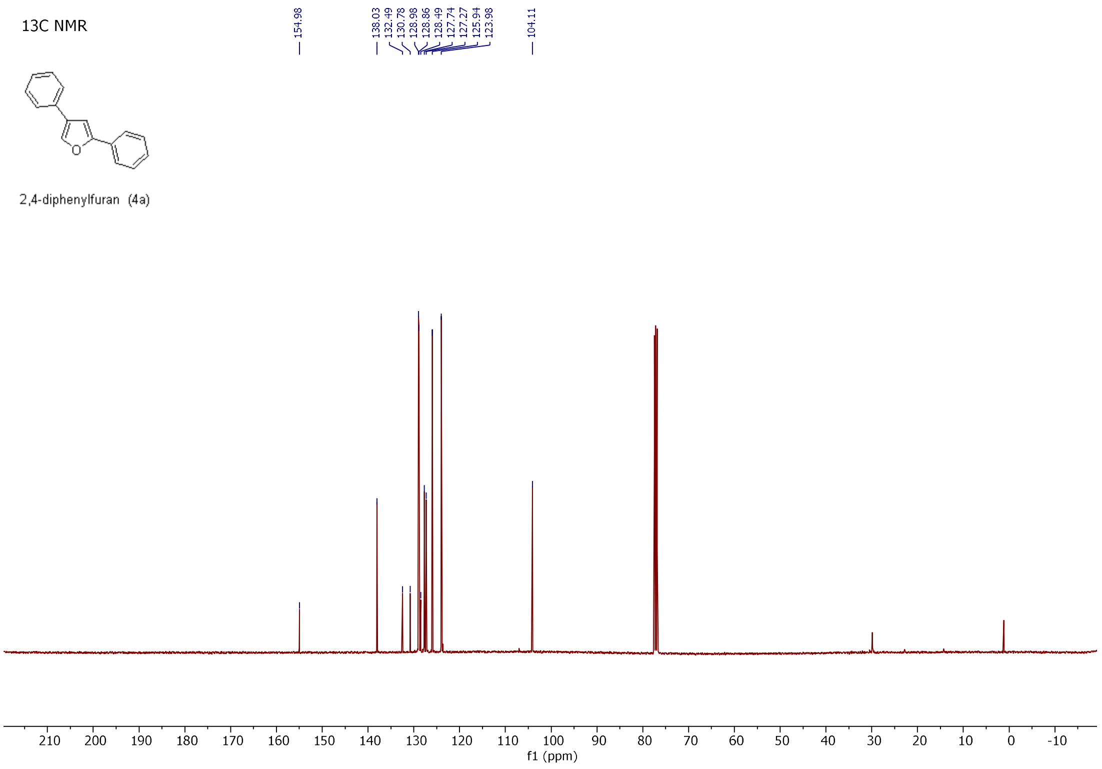

Supplementary Figure 81. NMR spectrum of 4a.

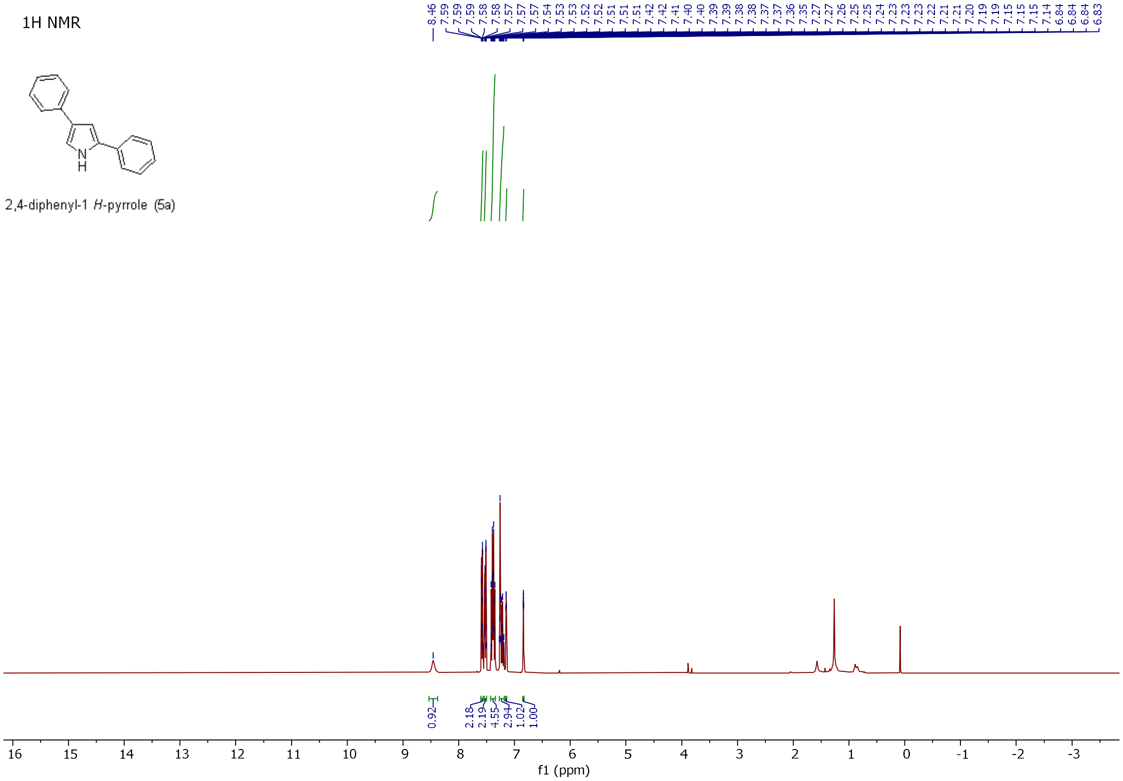

Supplementary Figure 82. NMR spectrum of 5a.

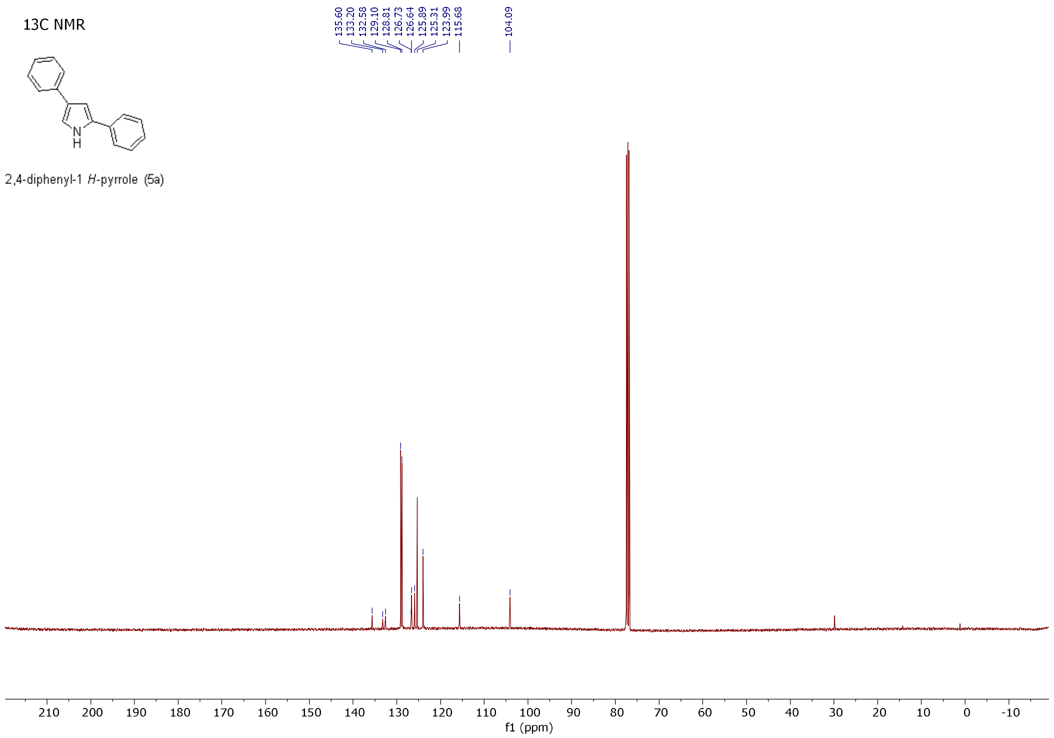

Supplementary

Figure 83. NMR spectrum of 5a.

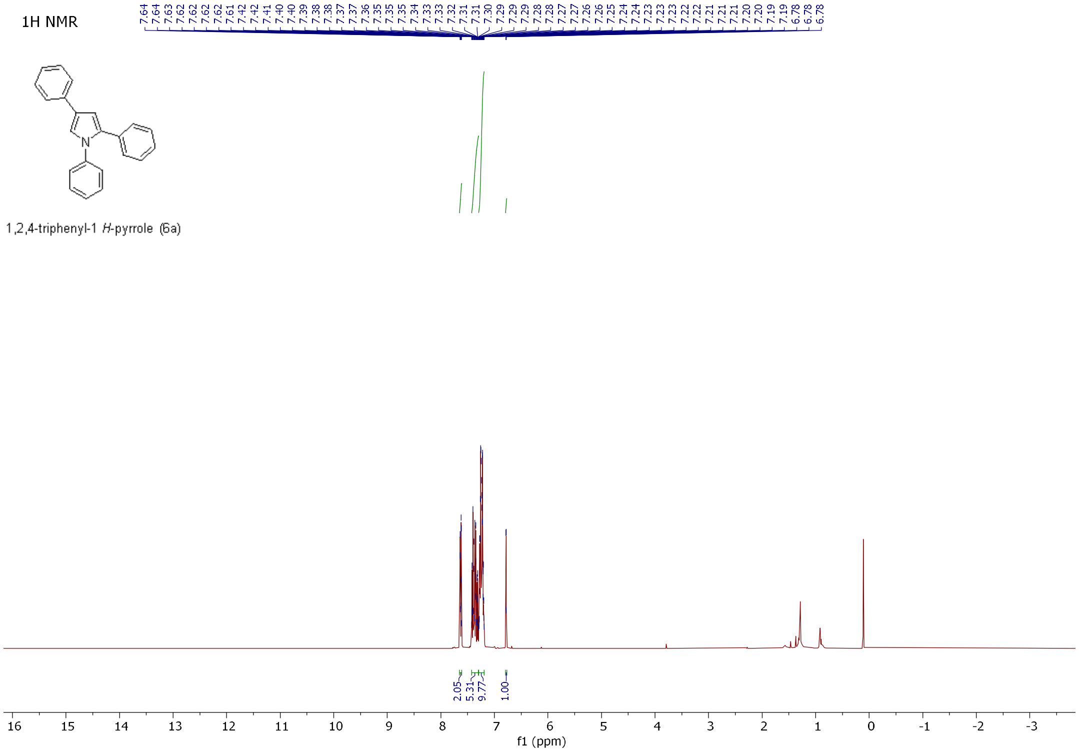

Supplementary Figure 84. NMR spectrum of 6a.

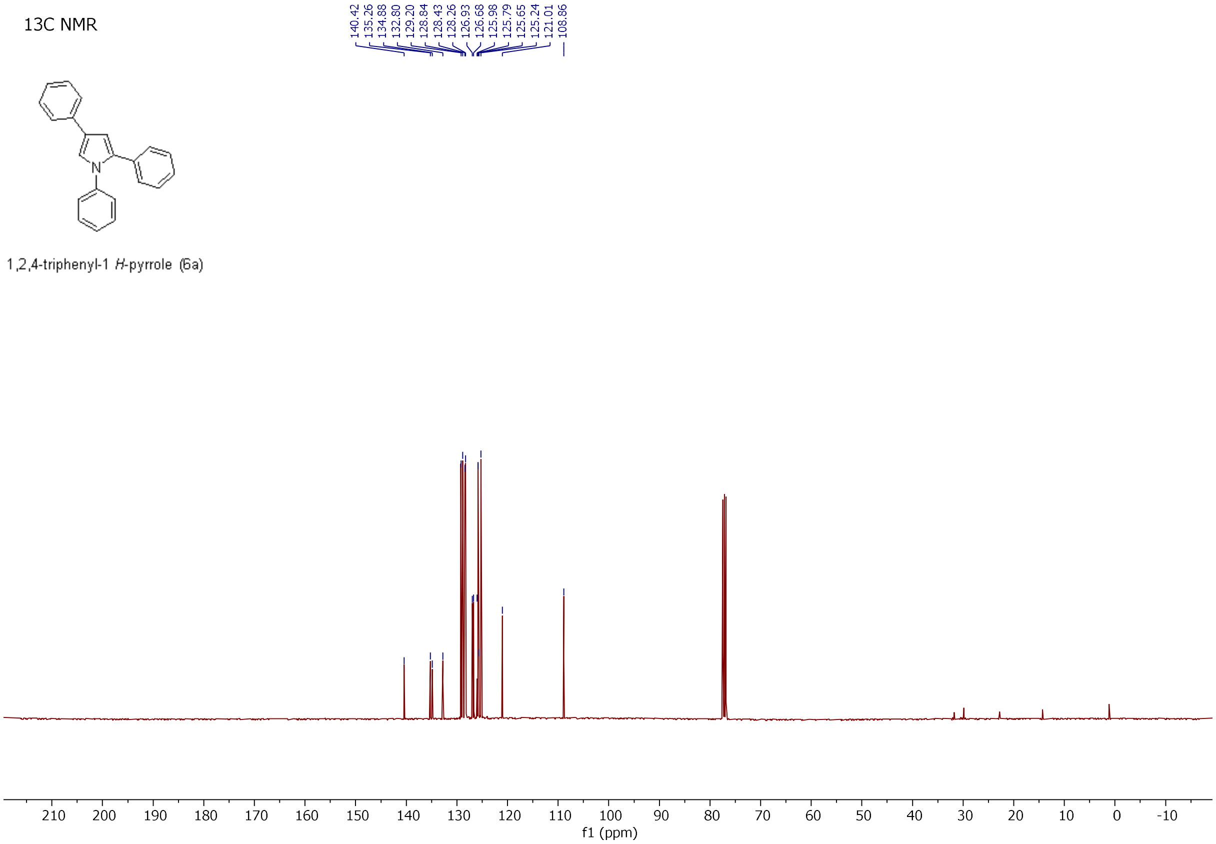

Supplementary Figure 85. NMR spectrum of 6a.

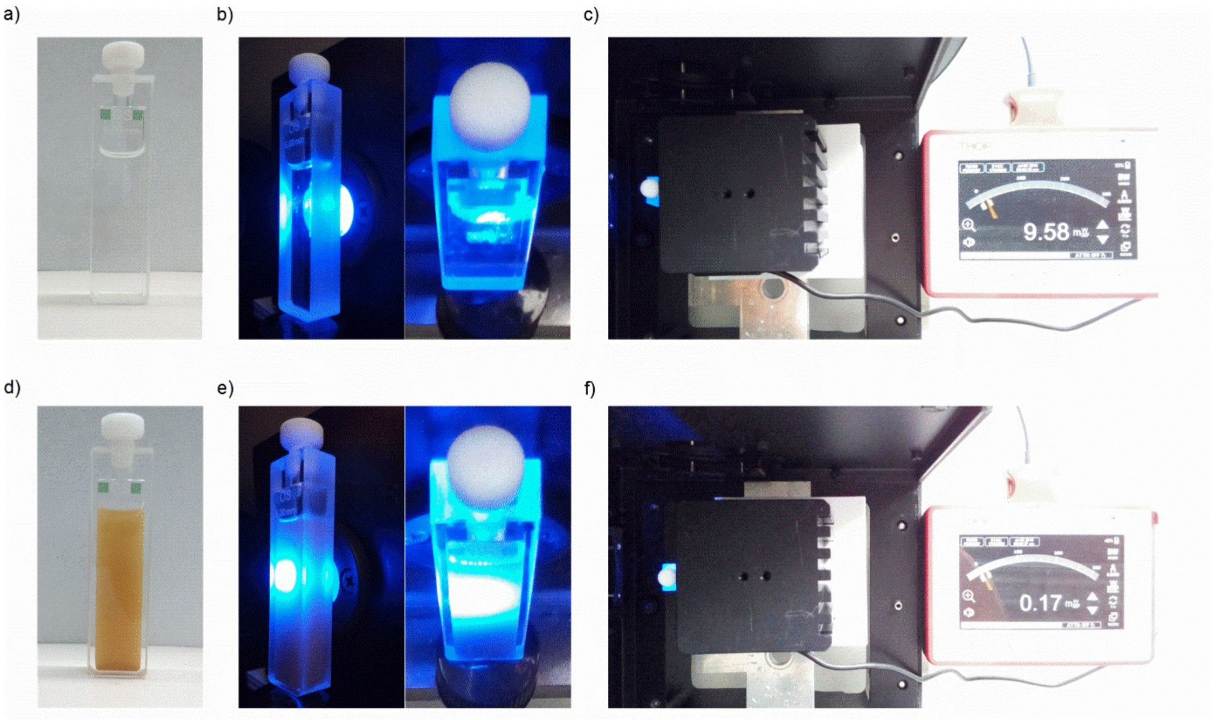

Supplementary Figure 86. Light scattering by K-PHI suspension. a) 5 mm cuvette filled with $CHCl_3$:DMSO (9:1) under day light; b) 5 mm cuvette filled with $CHCl_3$:DMSO (9:1) under 461±20 nm (I_0 = 10.6 mW cm^{-2}) does not scatter light (no halo in the middle of the cuvette); c) Measurement of light intensity transmitted through a 5 mm cuvette filled with $CHCl_3$:DMSO (9:1). Integrating sphere S142C connected to PM400 Optical Power and Energy Meter with readings on the left are shown; d) 5 mm cuvette filled with a suspension of K-PHI (2.5 mg mL^{-1}) in $CHCl_3$:DMSO (9:1) under day light; e) 5 mm cuvette filled with a suspension of K-PHI (2.5 mg mL^{-1}) in $CHCl_3$:DMSO (9:1) under 461±20 nm (I_0 = 10.6 mW cm^{-2}) scatters light strongly (strong halo in the middle of the cuvette); f) Measurement of light intensity transmitted through a 5 mm cuvette filled with a suspension of K-PHI (2.5 mg mL^{-1}) in $CHCl_3$:DMSO (9:1). Integrating sphere S142C connected to PM400 Optical Power and Energy Meter with readings on the left are shown.

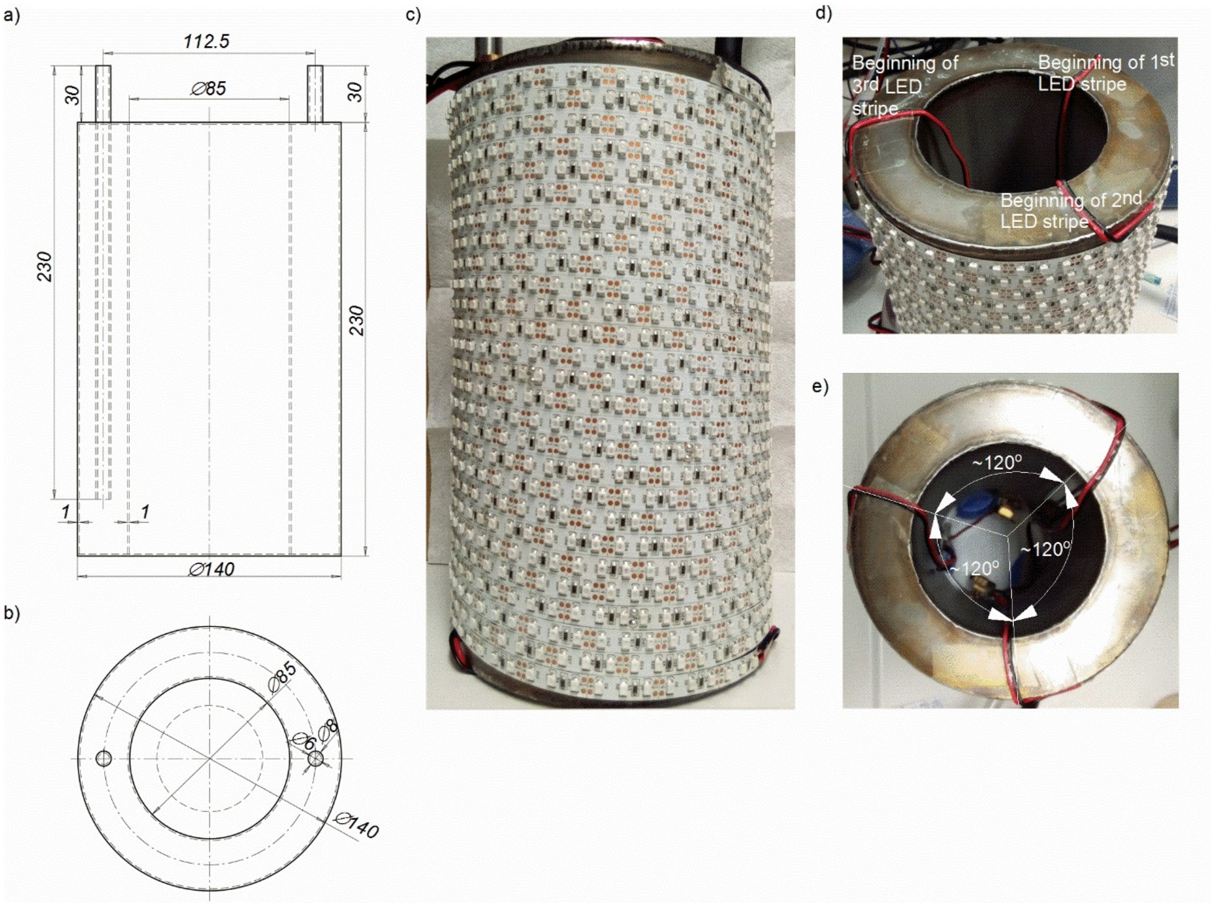

Supplementary Figure 87. a) Side view of the steel cylinder; b) Top view of the steel cylinder; c) Side view of the assempled light source; d) and e) Bottom view of the assembled light source. Displacement of ca. 120° between the leading ends of the LED stripes is shown.

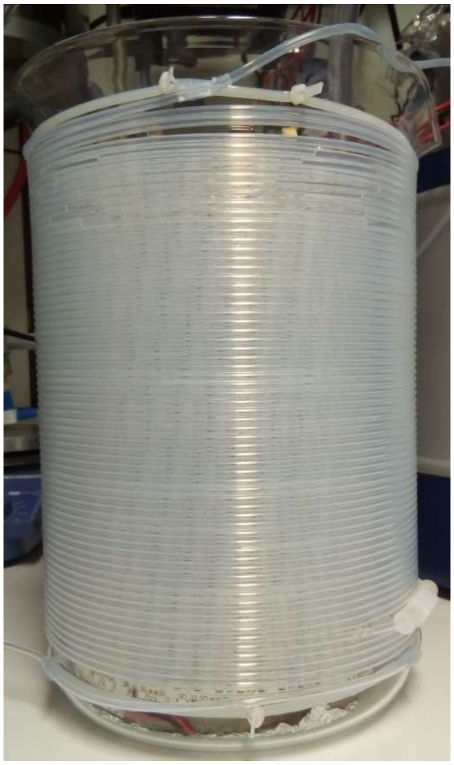

Supplementary Figure 88. Flow photoreactor tubing.

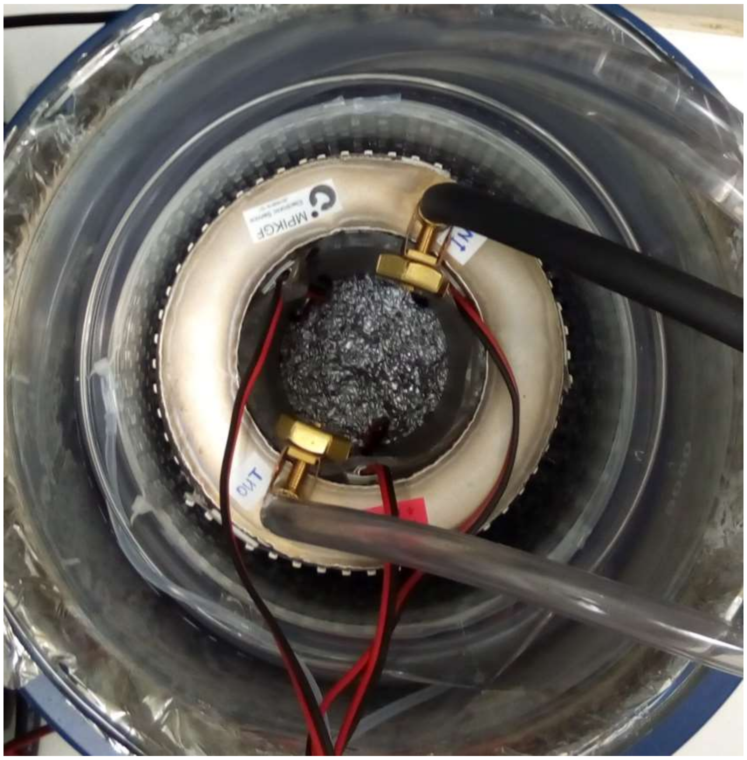

Supplementary Figure 89. External shell of the flow photoreactor.

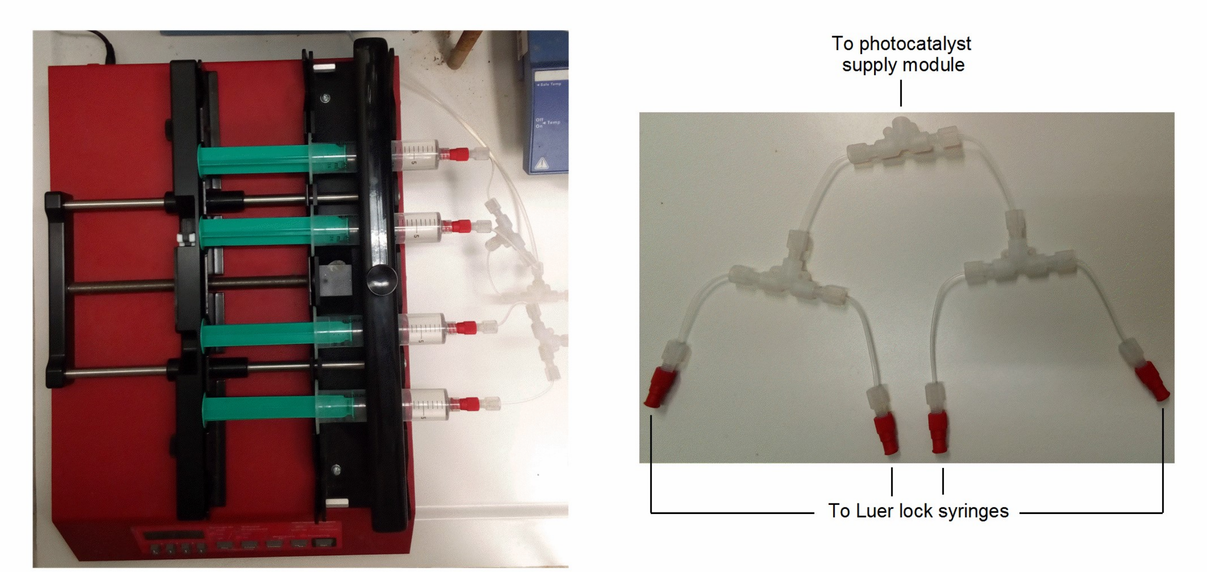

Supplementary Figure 90. Reaction mixture supply module.

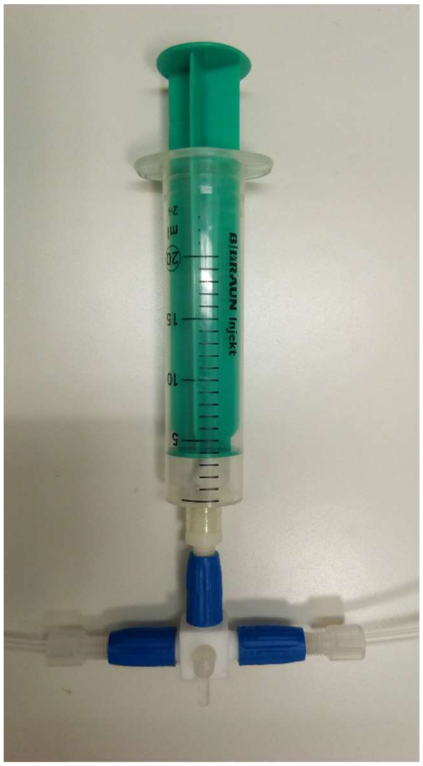

Supplementary Figure 91. Photocatalyst supply module.

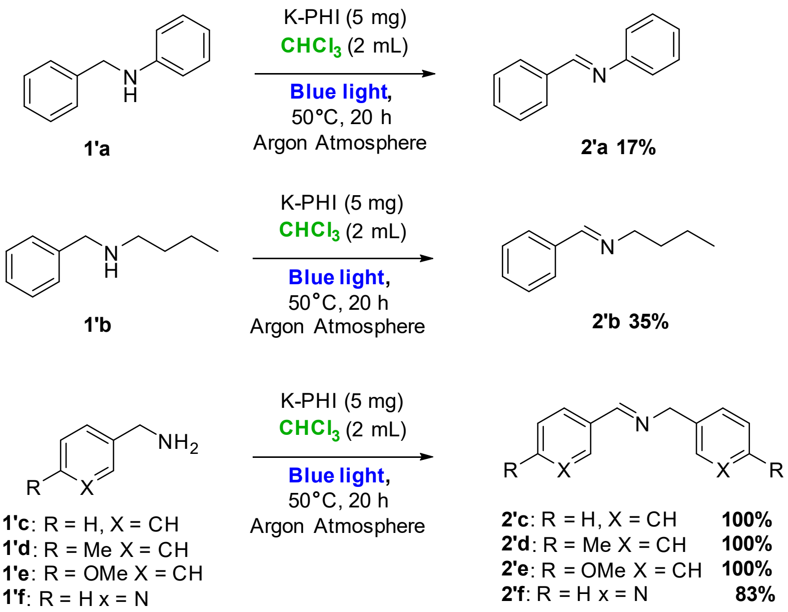

Supplementary Figure 92. Scope of imines. Yields were calculated by GC-MS

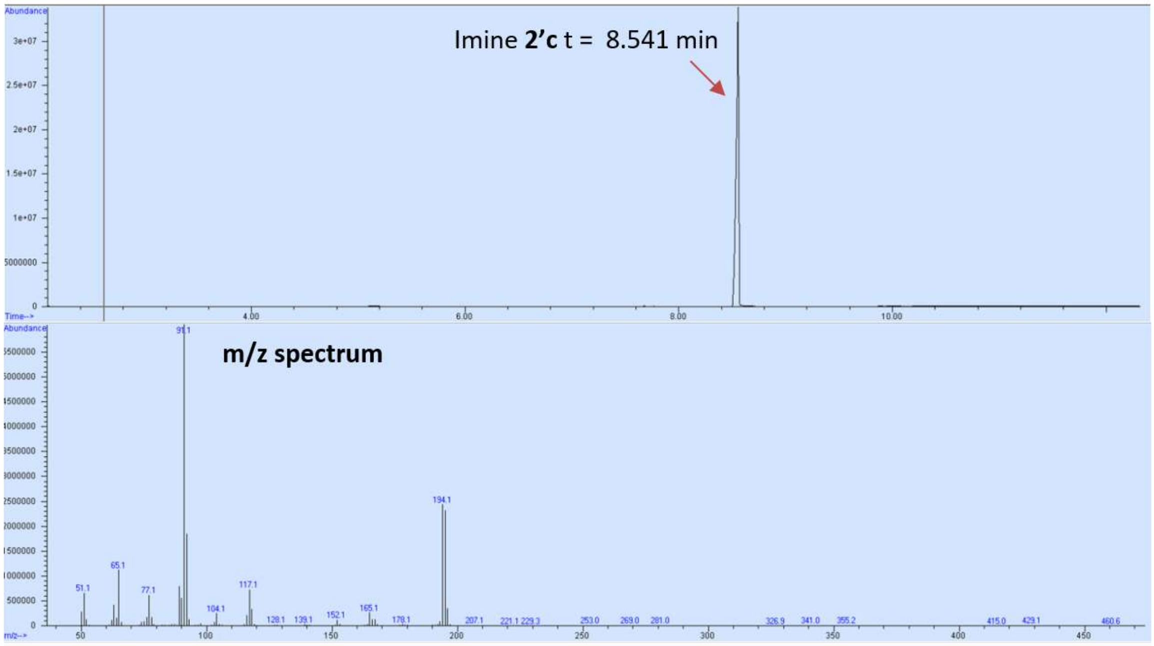

Supplementary Figure 93. Typical chromatogram after imine formation.

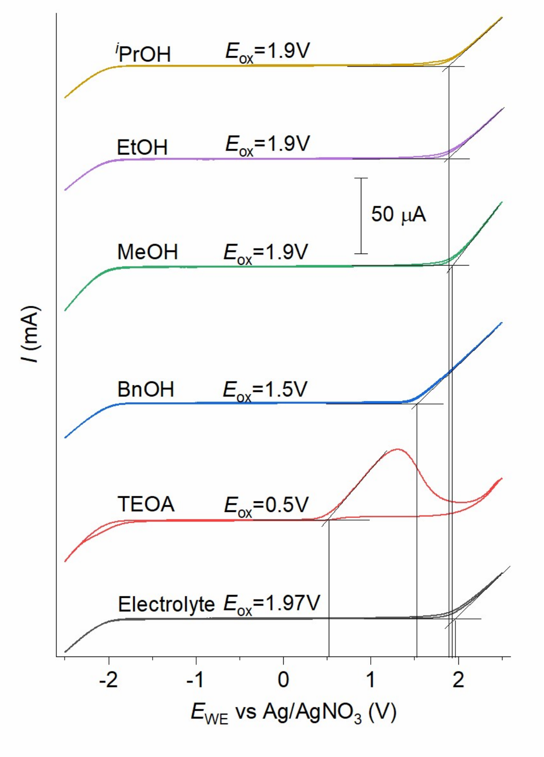

Supplementary Figure 94. Cyclic voltamogramms of different hole scavengers. From bottom to top: a) electrolyte $(n\text{Bu})_4N^+$ ClO_4^- in chloroform (0.1M) purged with Ar; b) TEOA solution (50 mM) in electrolyte shows E_{OX} = +0.5 V; c) BnOH solution (50 mM) in electrolyte shows E_{OX} = +1.5 V; d) MeOH solution (50 mM) in electrolyte shows E_{OX} = +1.9 V; e) EtOH solution (50 mM) in electrolyte shows E_{OX} = +1.9 V; f) iPrOH solution (50 mM) in electrolyte shows E_{OX} = +1.9 V.

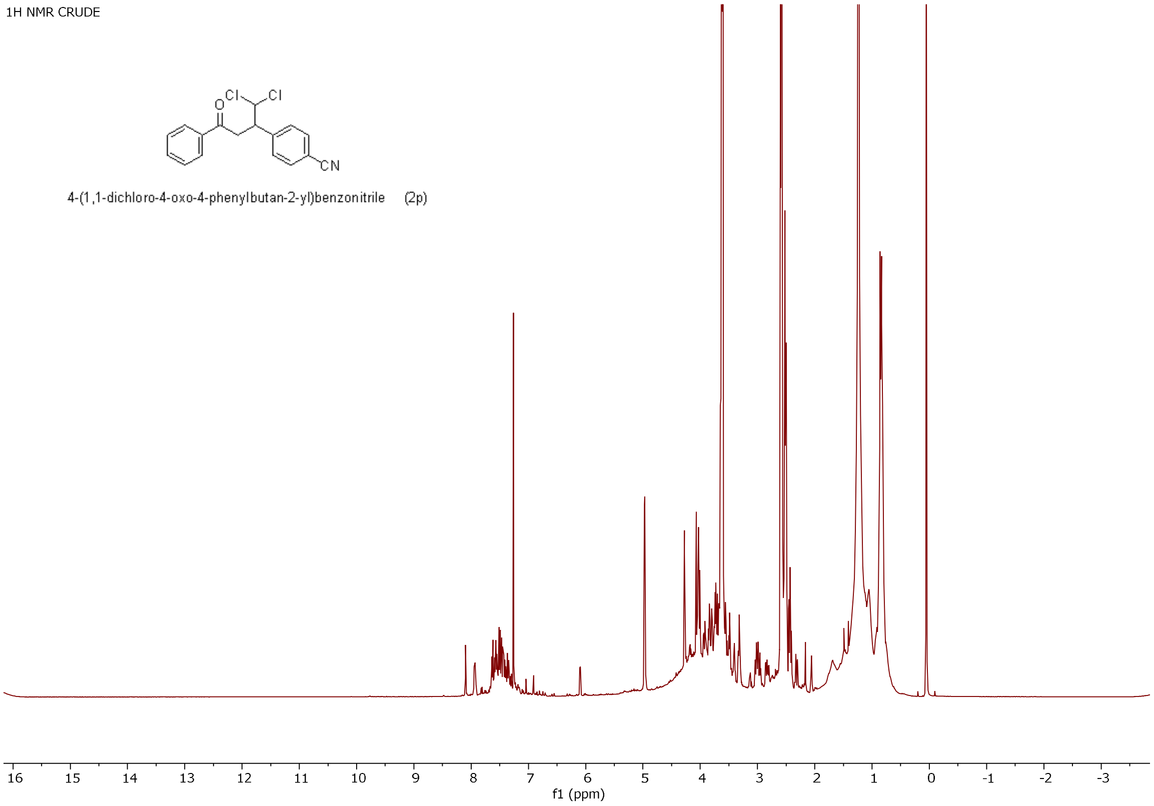

Supplementary Figure 95 - NMR spectrum of 2p.

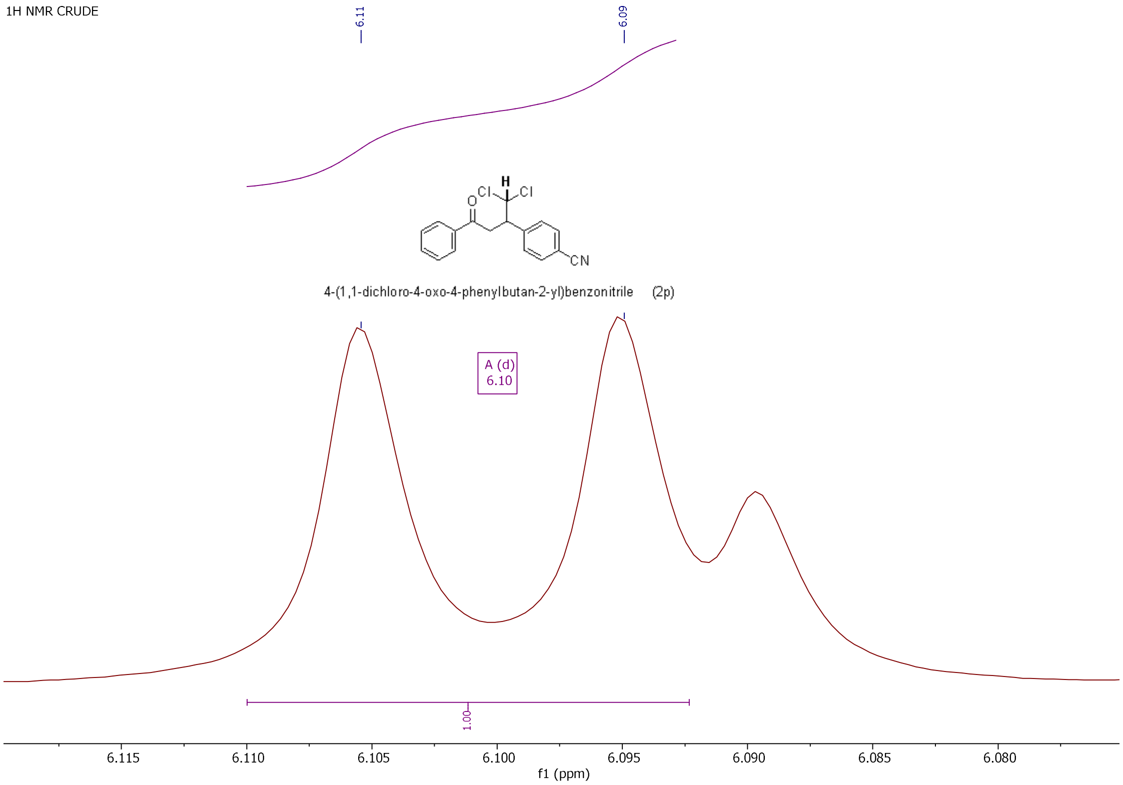

Supplementary Figure 96 - NMR spectrum of **2p**.

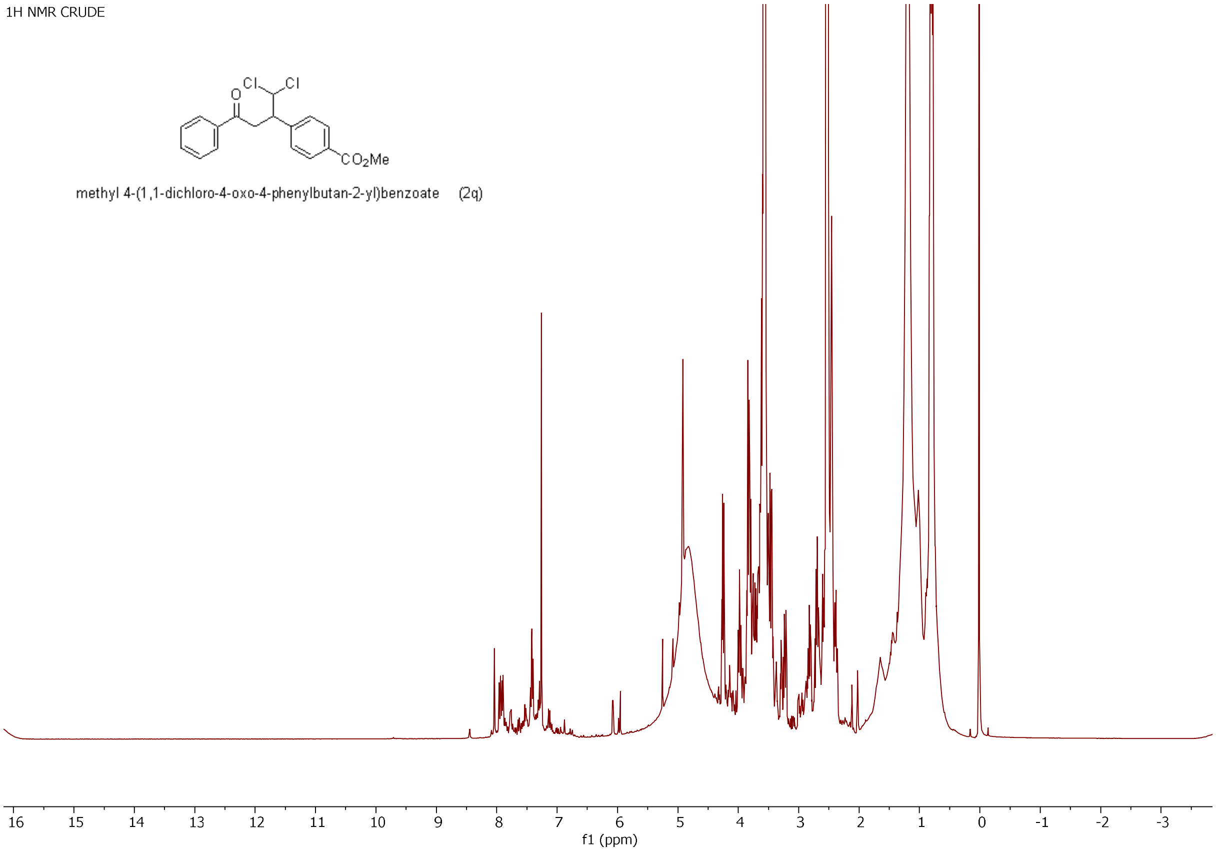

Supplementary Figure 97 - NMR spectrum of 2q.

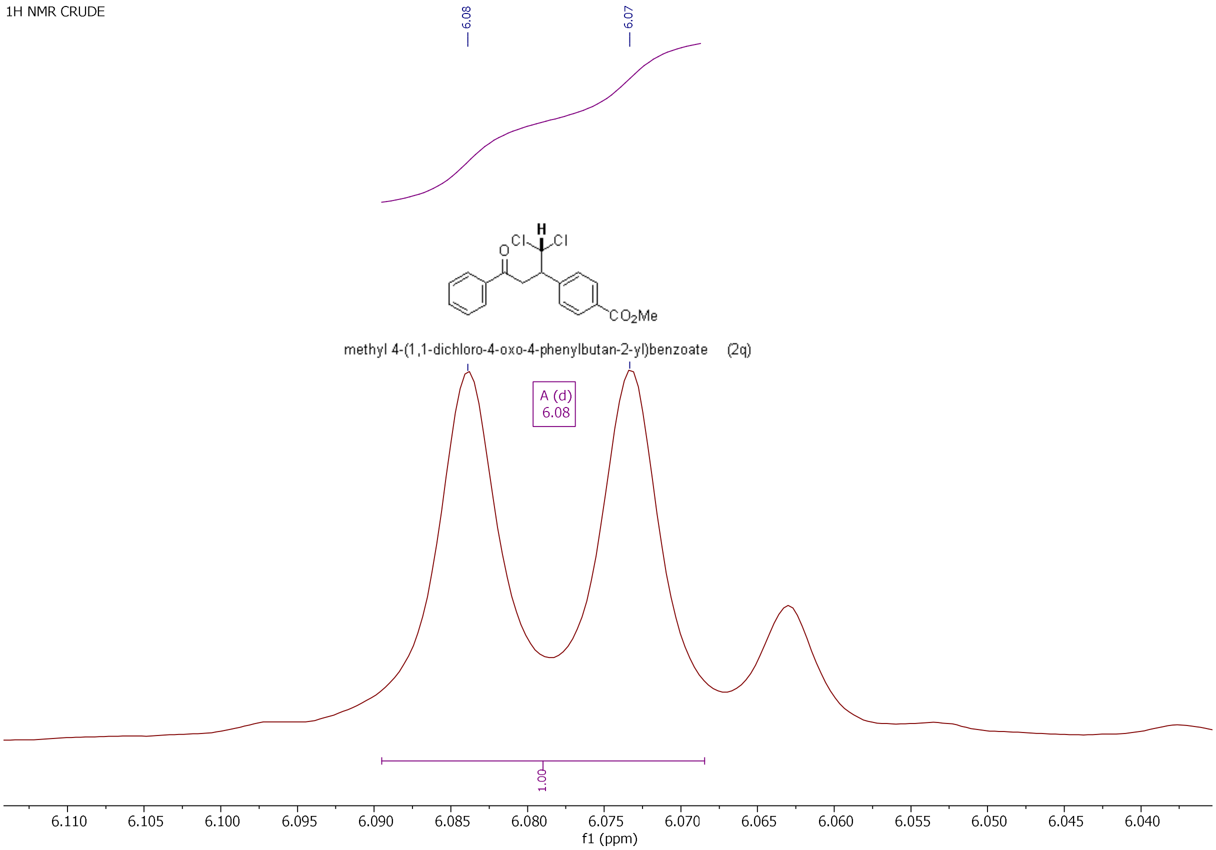

Supplementary Figure 98 - NMR spectrum of **2q**.

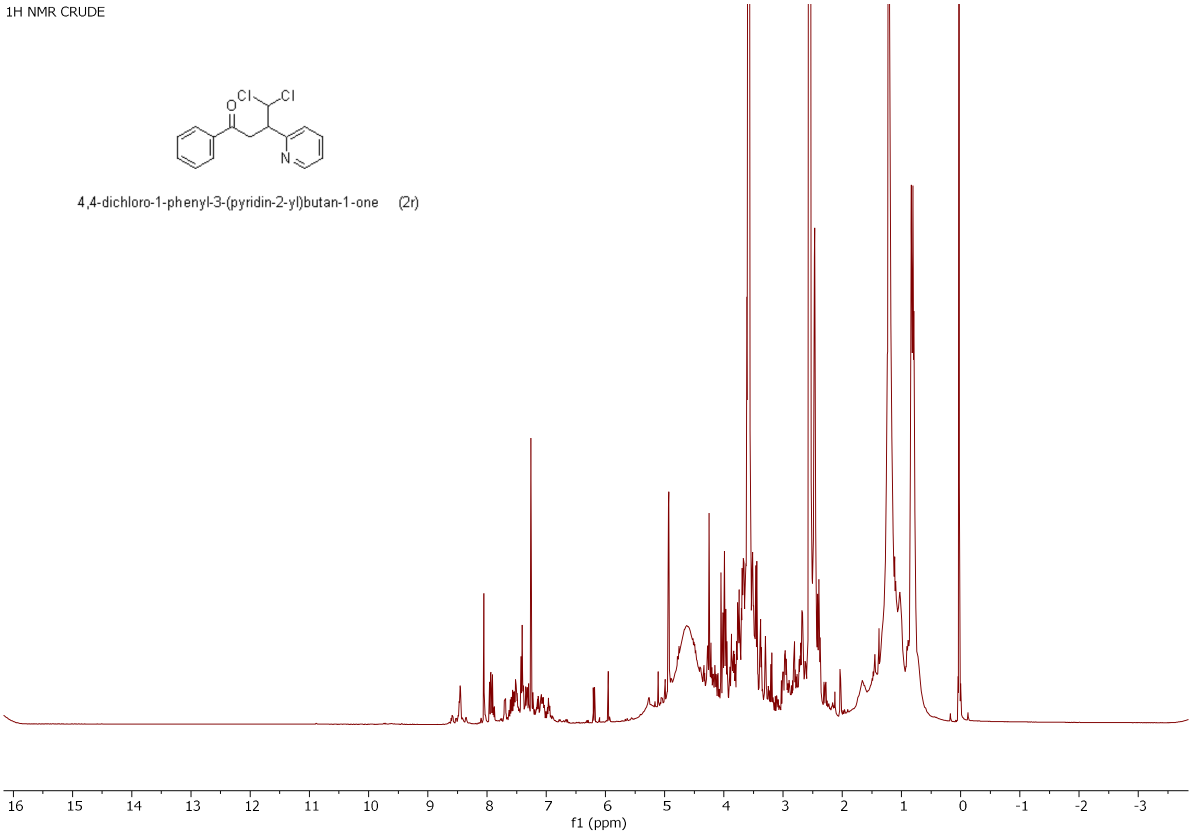

Supplementary Figure 99 - NMR spectrum of **2r**.

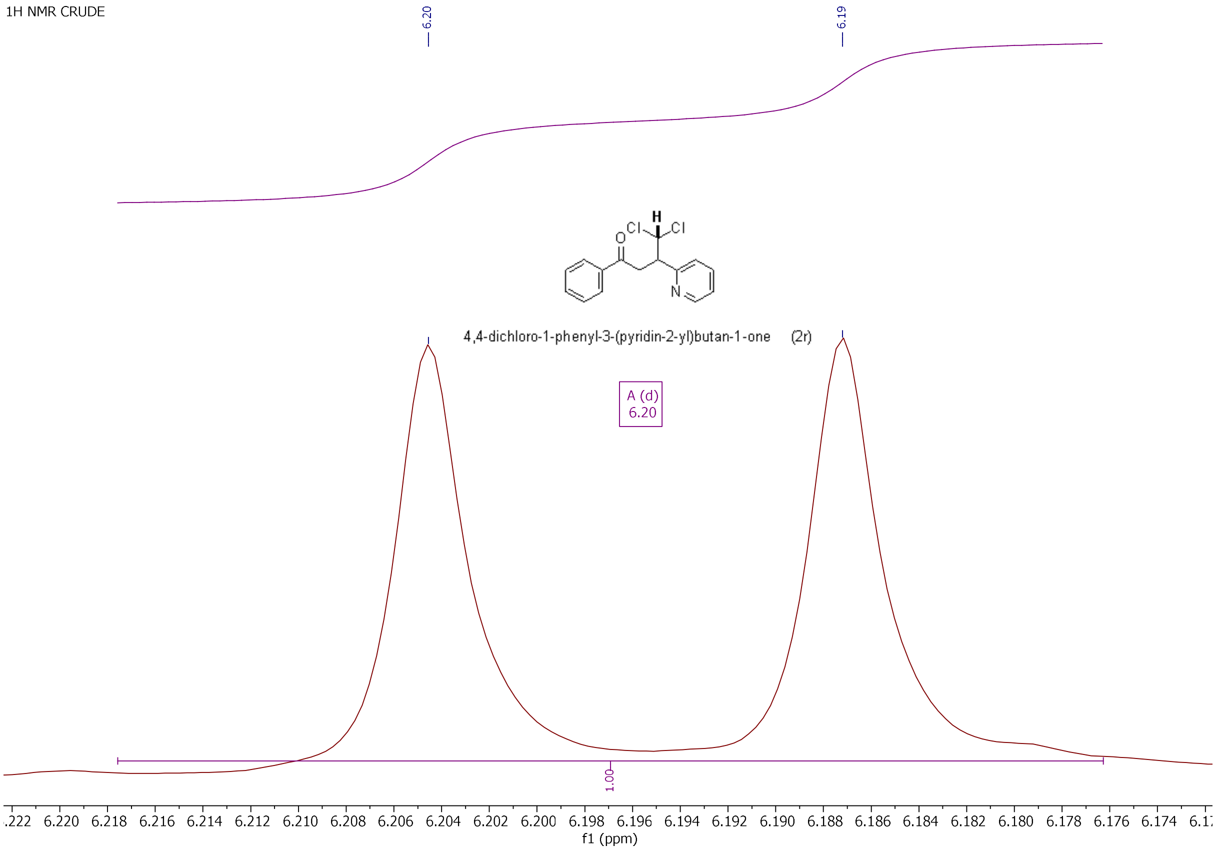

Supplementary Figure 100 - NMR spectrum of 2r.

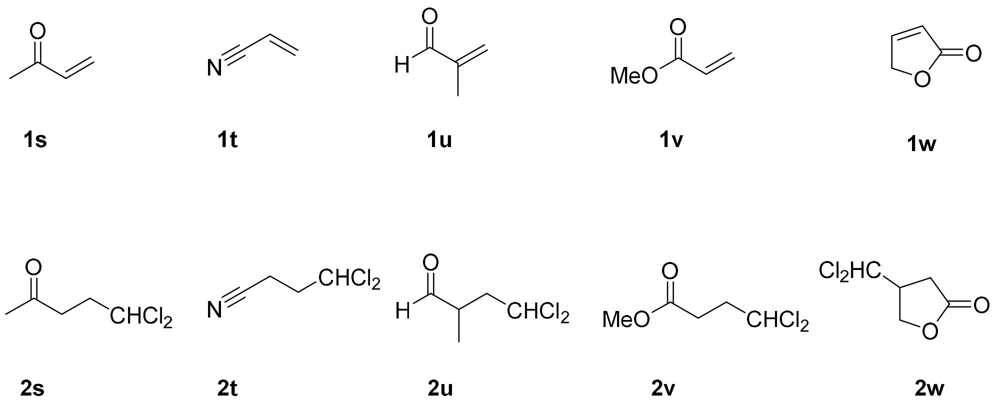

Supplementary Figure 101 – Scope of other Michael acceptors

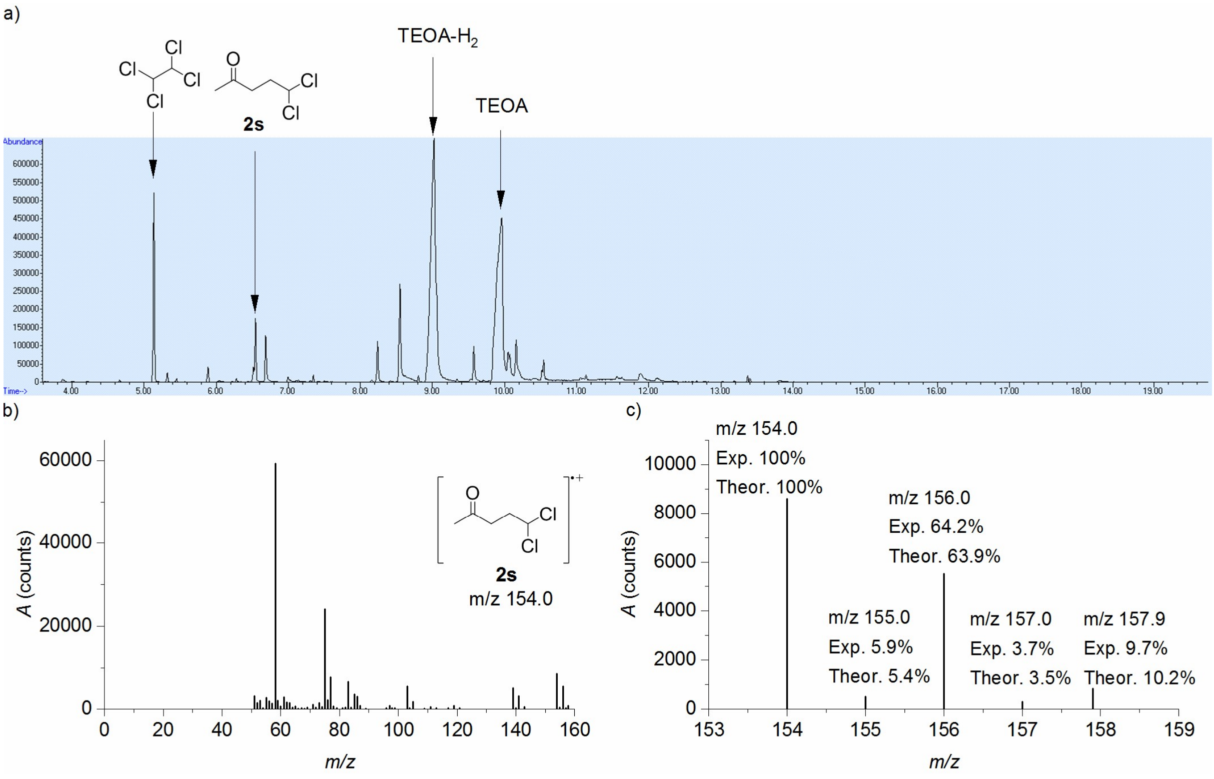

Supplementary Figure 102 - GC-MS chromatogram of **2s**. (a) GC-MS chromatogram of the reaction mixture using methyl vinyl ketone 1s as a reagent in dichloromethylation reaction. Main reaction mixture components peaks are labeled. b) Mass-spectrum of 2s radical cation with the theoretical m/z value. c) Zoomed in area of the mass spectrum. Experimental and theoretical m/z intensities with respect to the intensity of **2s** radical cation (m/z 154.0) are shown.

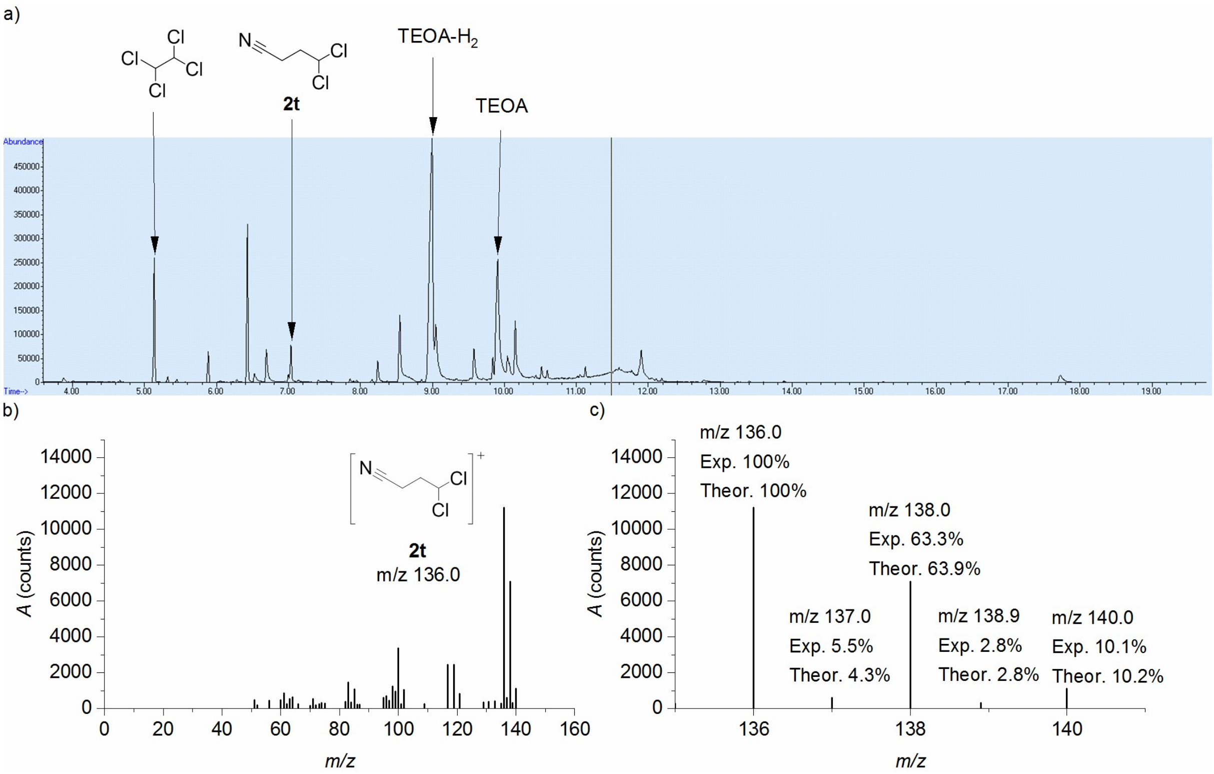

Supplementary Figure 103 - GC-MS chromatogram of **2t**. (a) GC-MS chromatogram of the reaction mixture using acrylonitrile **1t** as a reagent in dichloromethylation reaction. Peaks in GC of the main components of the reaction misture are labeled. b) Mass-spectrum of **2t** cation with the theoretical m/z value. c) Zoomed in area of the mass spectrum. Experimental and theoretical m/z intensities with respect to the intensity of **2t** cation (m/z 136.0) are shown.

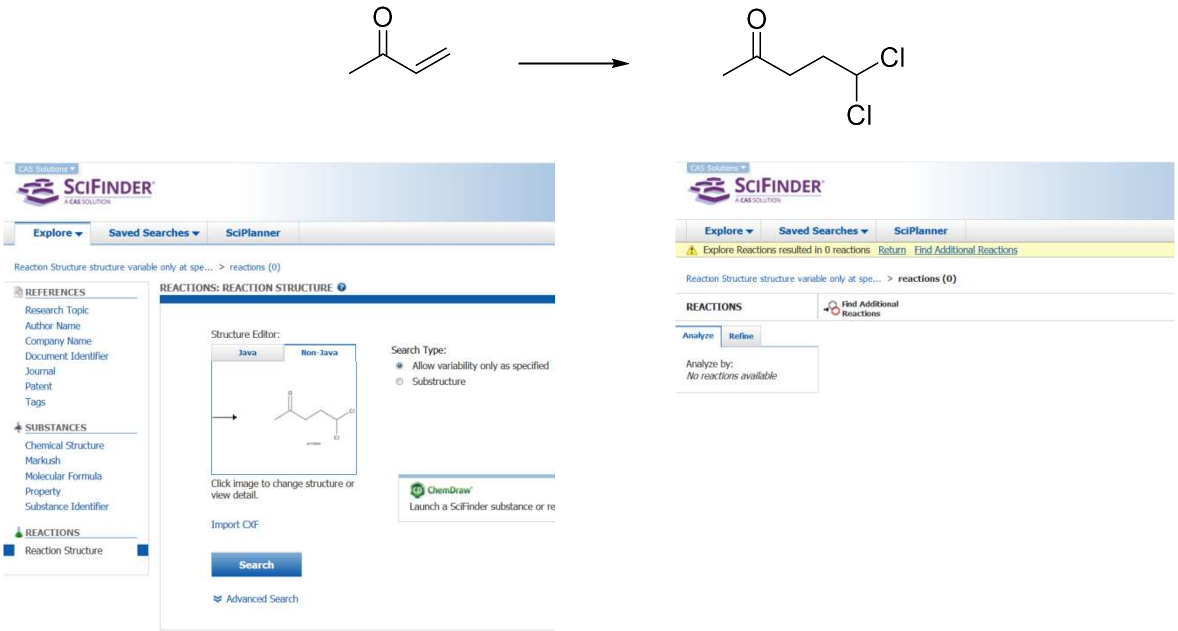

Supplementary Figure 104 – SciFinder outcome for preparation of 2s

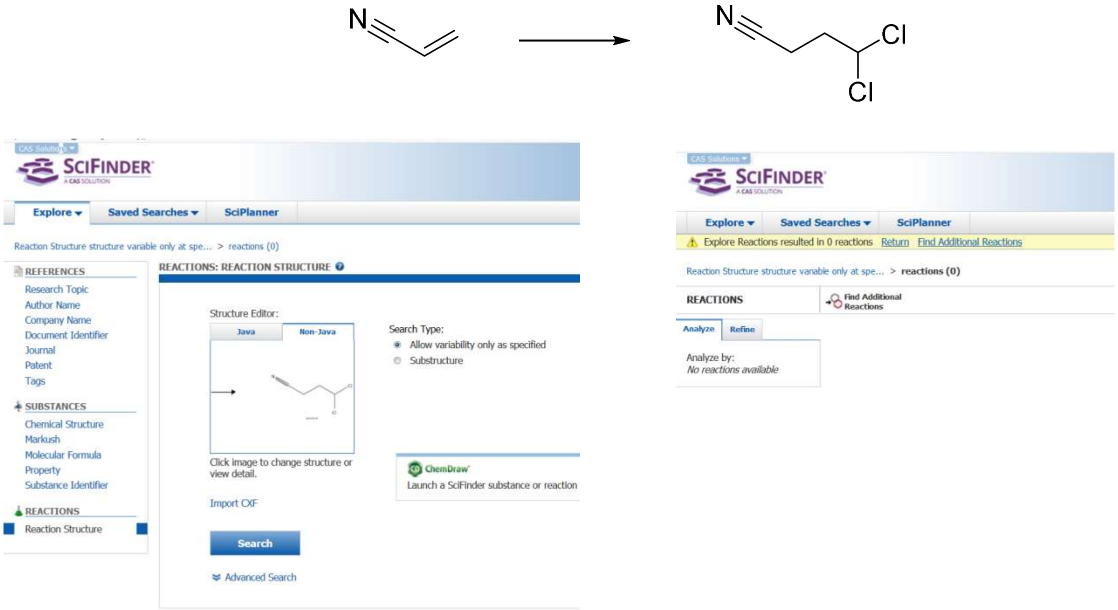

Supplementary Figure 105 – SciFinder outcome for preparation of 2t

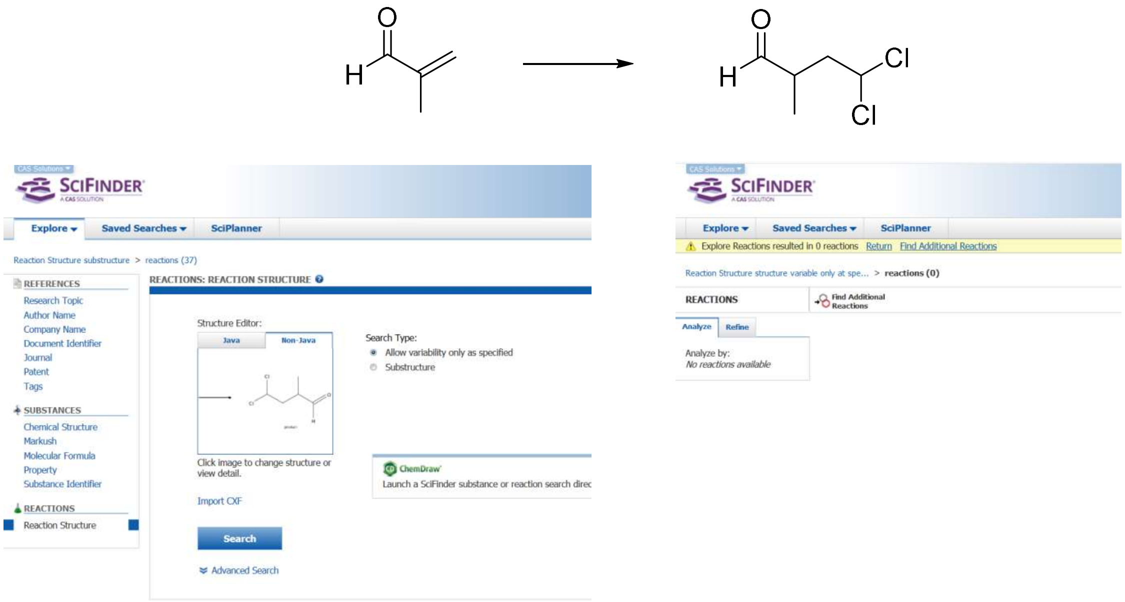

Supplementary Figure 106 – SciFinder outcome for preparation of 2u

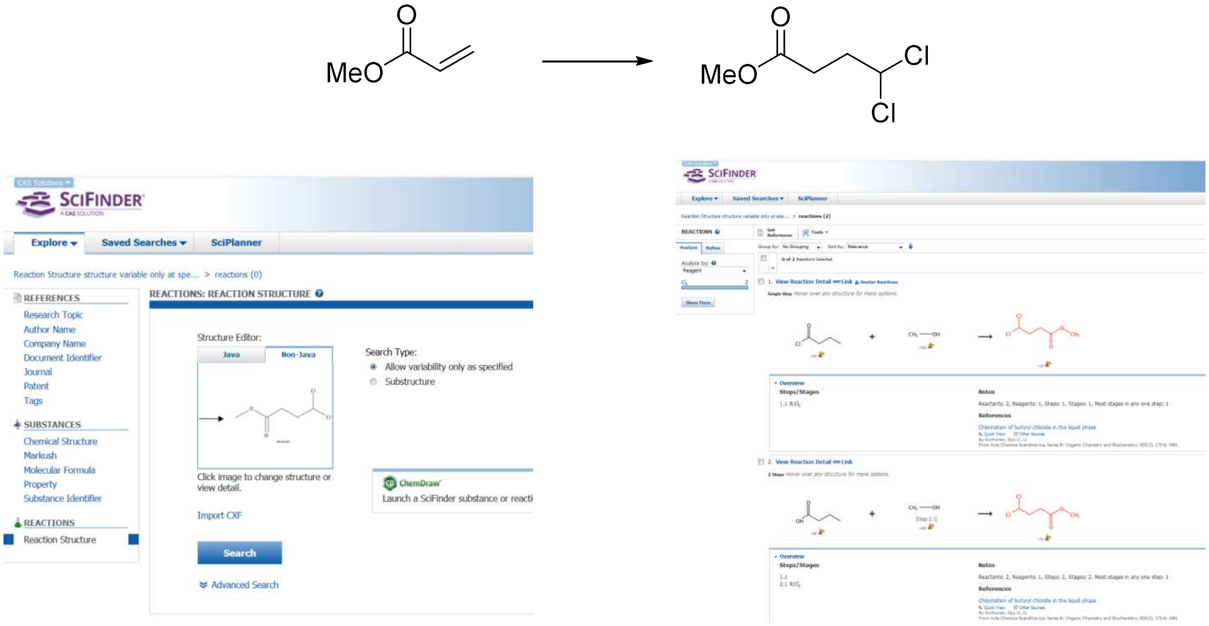

Supplementary Figure 107 – SciFinder outcome for preparation of 2v

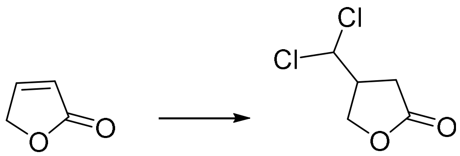

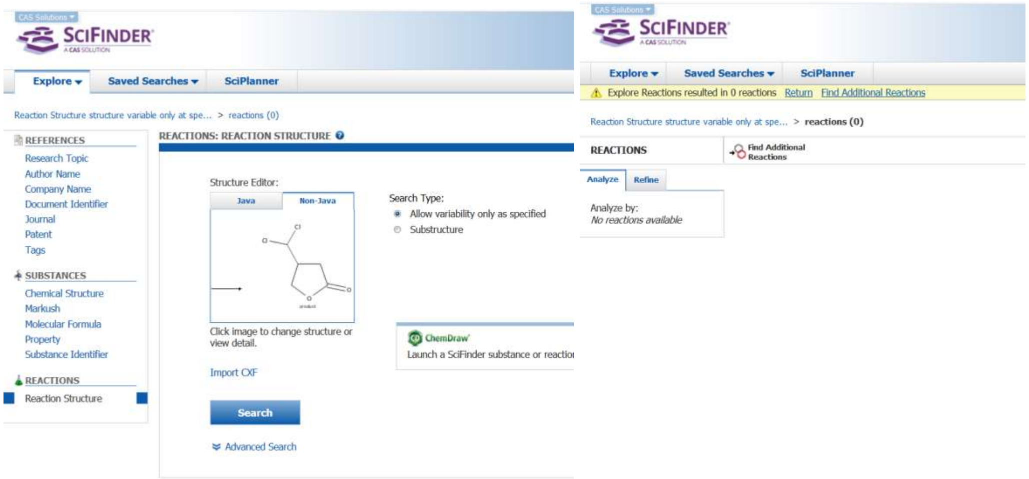

Supplementary Figure 108 – SciFinder outcome for preparation of 2w

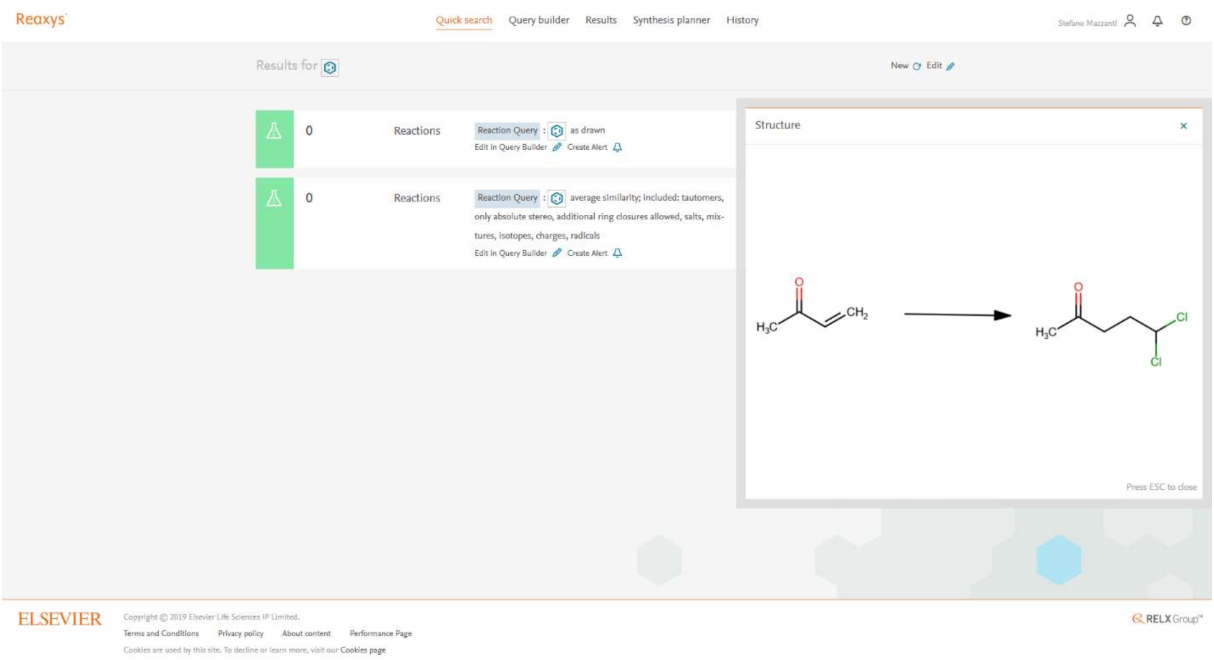

Supplementary Figure 109 – Reaxys outcome for the preparation of 2s

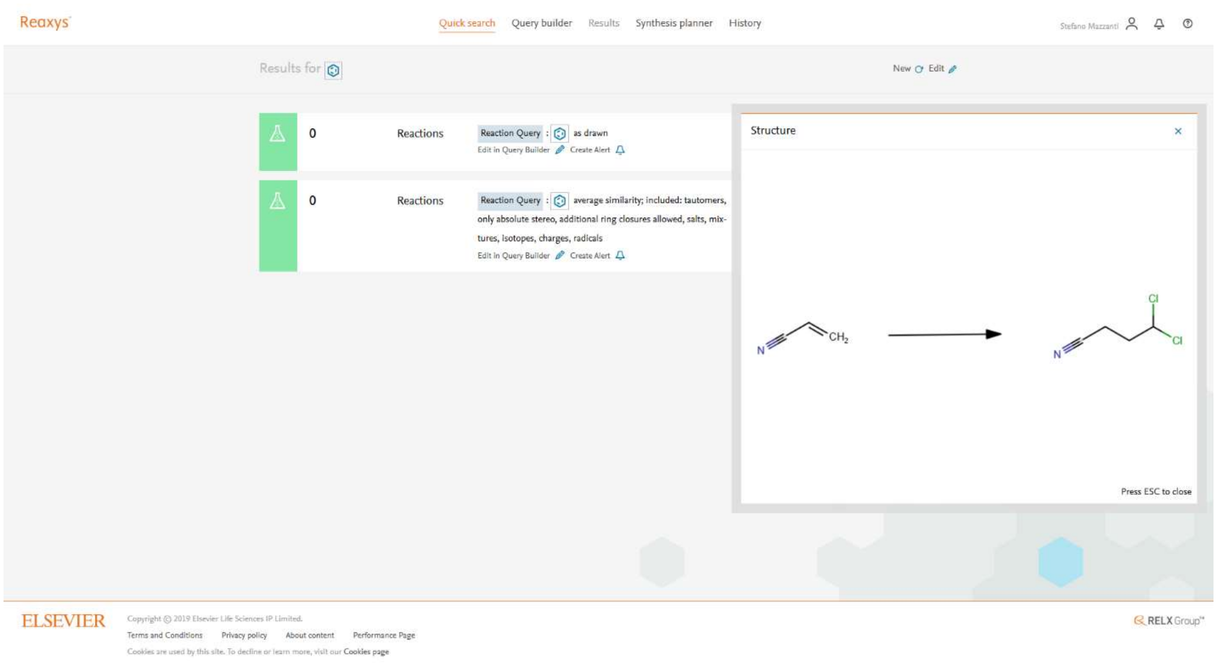

Supplementary Figure 110 – Reaxys outcome for the preparation of 2t

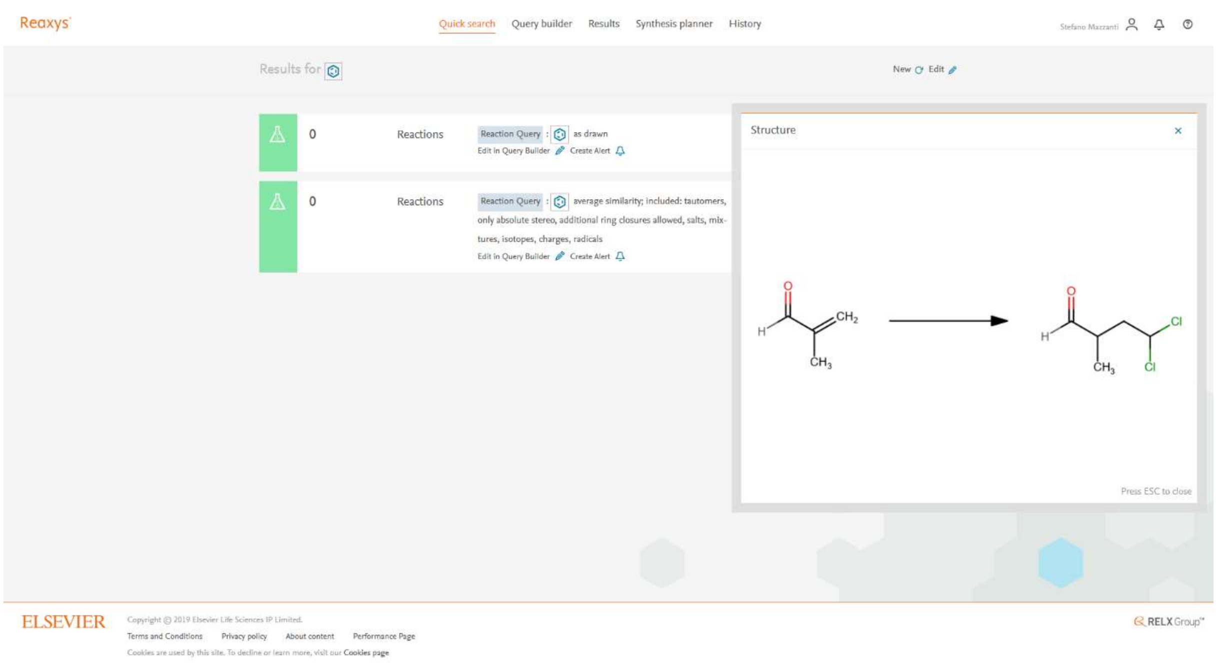

Supplementary Figure 111 – Reaxys outcome for the preparation of 2u

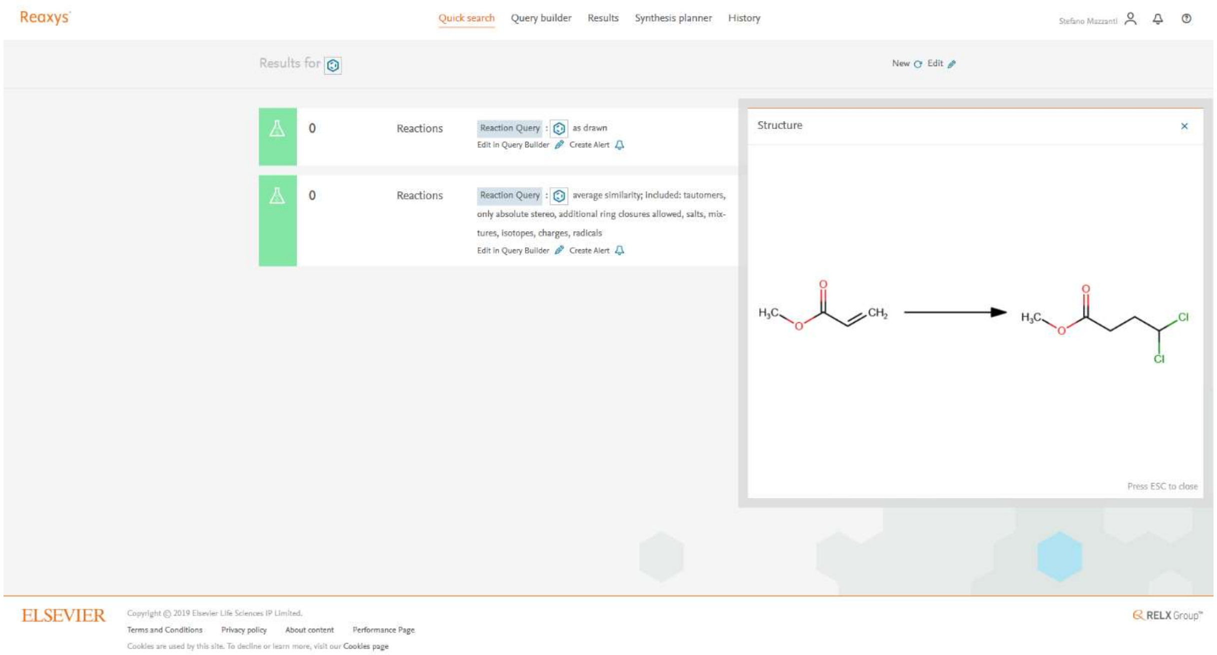

Supplementary Figure 112 – Reaxys outcome for the preparation of 2v

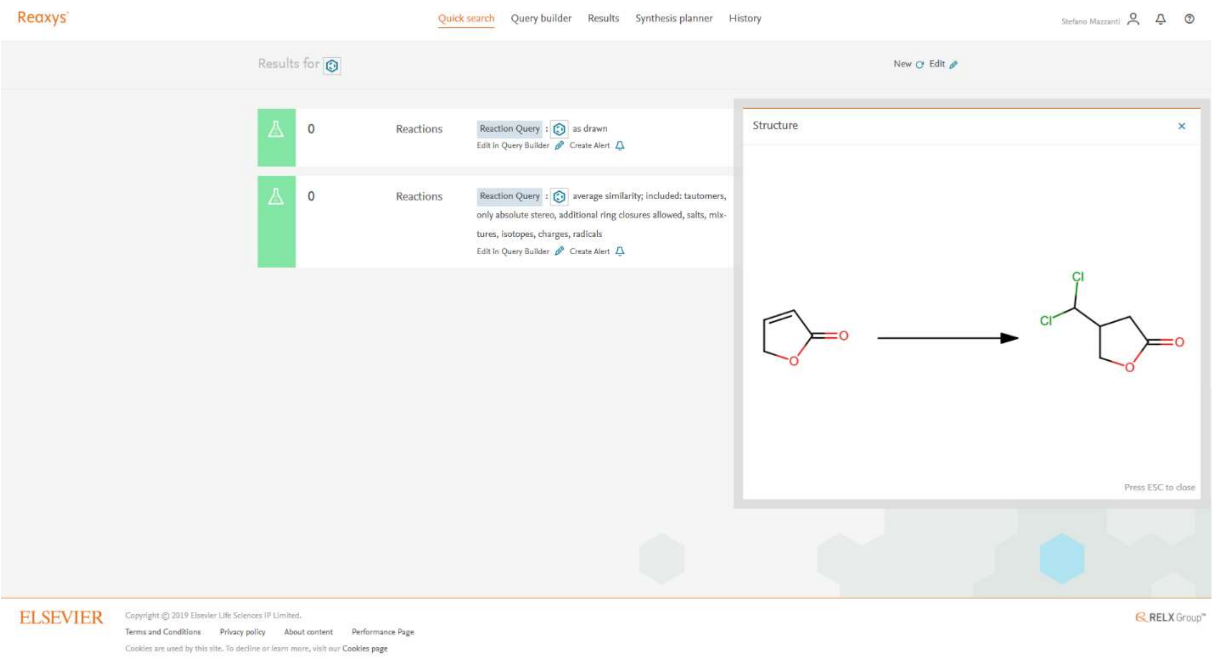

Supplementary Figure 113 – Reaxys outcome for the preparation of 2w

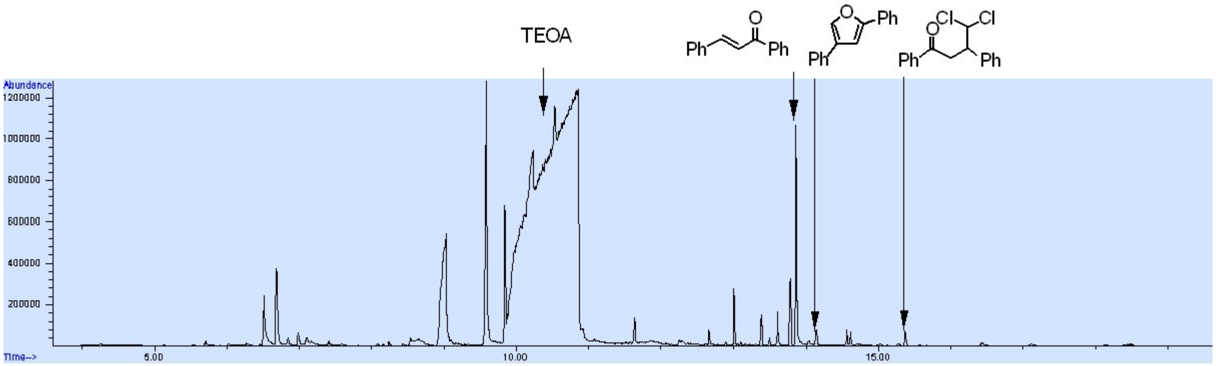

Supplementary Figure 114 - GC-MS data of the reaction mixture using dichloromethane as solvent.

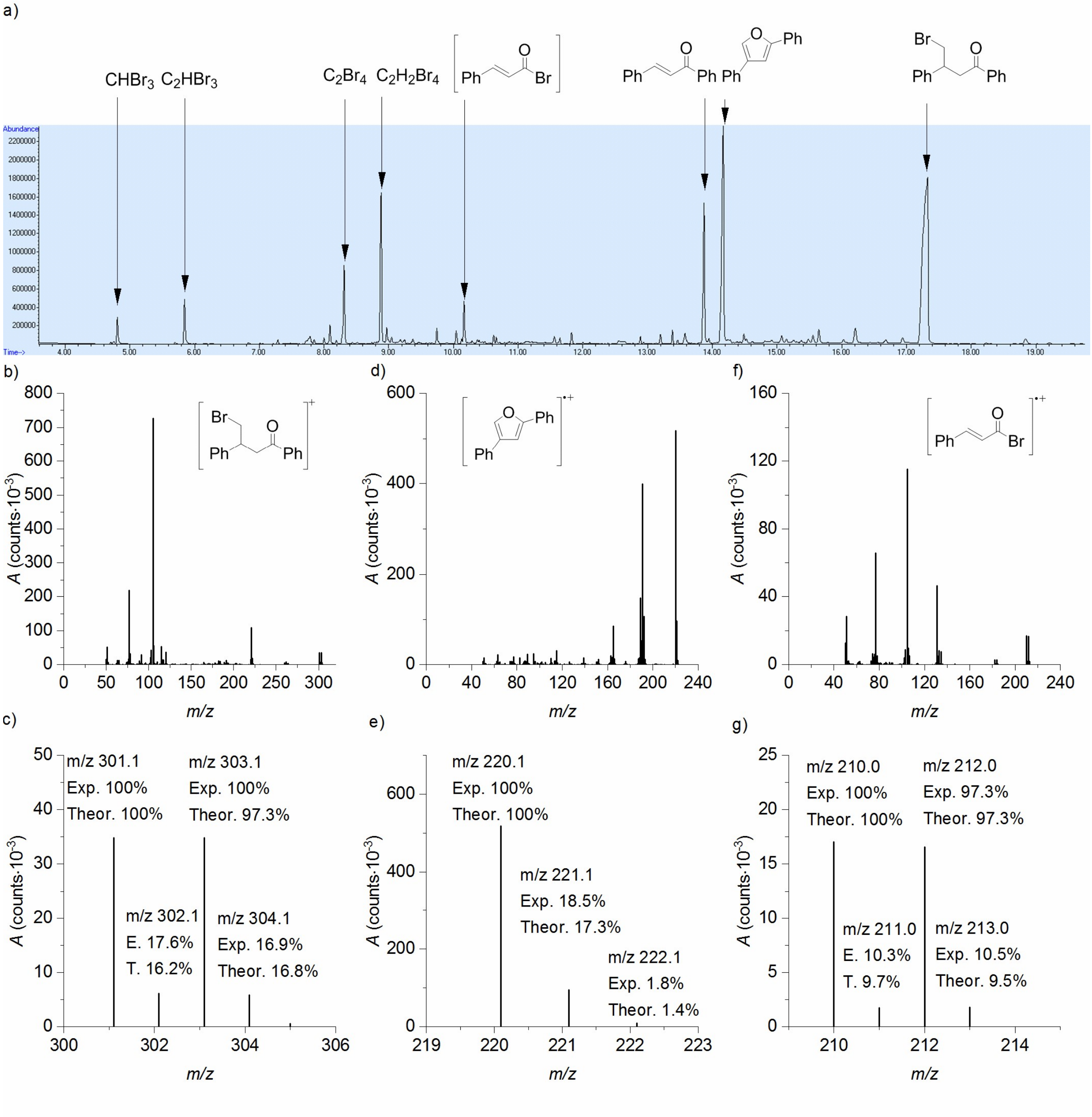

Supplementary Figure 115 - GC-MS data of the reaction mixture using bromoform as solvent. a) Gas chromatogram. b) Mass spectrum of the GC peak corresponding to 4-bromo-1,3-diphenylbutan-1-one cation. c) Zoomed in mass spectrum of 4-bromo-1,3-diphenylbutan-1-one cation. d) Mass spectrum of the GC peak corresponding to 2,4-diphenylfurane radical cation. e) Zoomed in mass spectrum of 2,4-diphenylfurane radical cation. f) Mass spectrum of the GC peak corresponding to the compound with a tentative structure of cinnamoyl bromide radical cation. g) Zoomed in mass spectrum of cinnamoyl bromide radical cation.

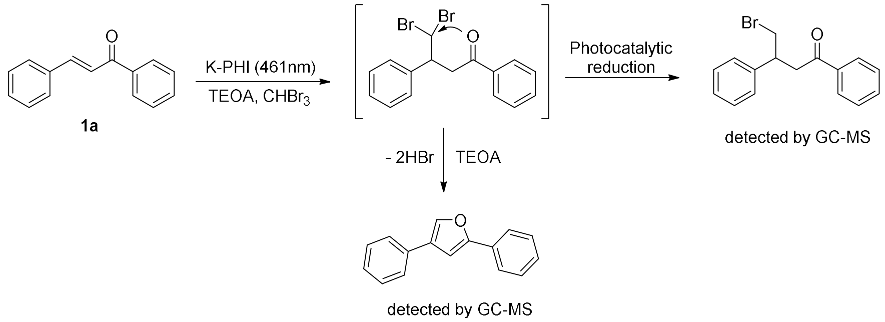

Supplementary Figure 116 – Proposed mechanism using bromoform as solvent

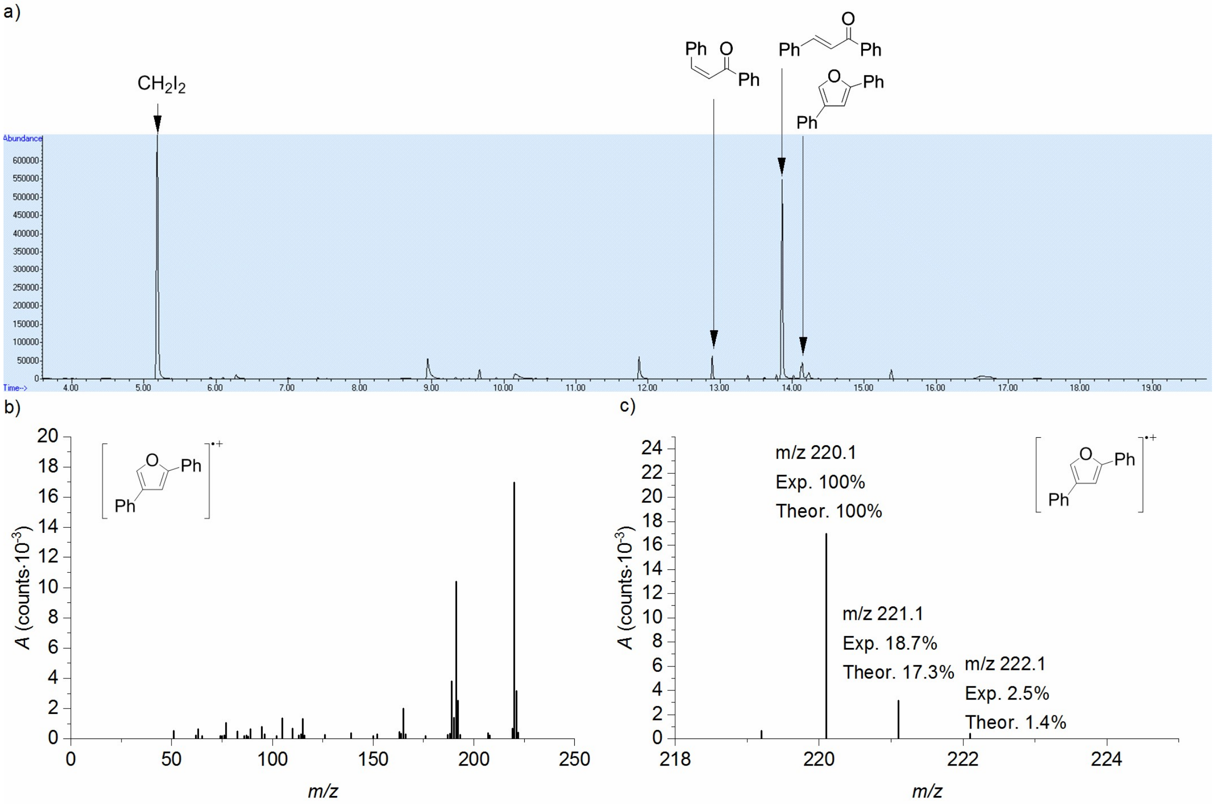

Supplementary Figure 117 - GC-MS data of the reaction mixture using iodoform in dichloromethane. a) Gas chromatogram. b) Mass spectrum of the GC peak corresponding to 2,4-diphenylfuran radical cation. c) Zoomed in mass spectrum of 2,4-diphenylfuran radical cation.

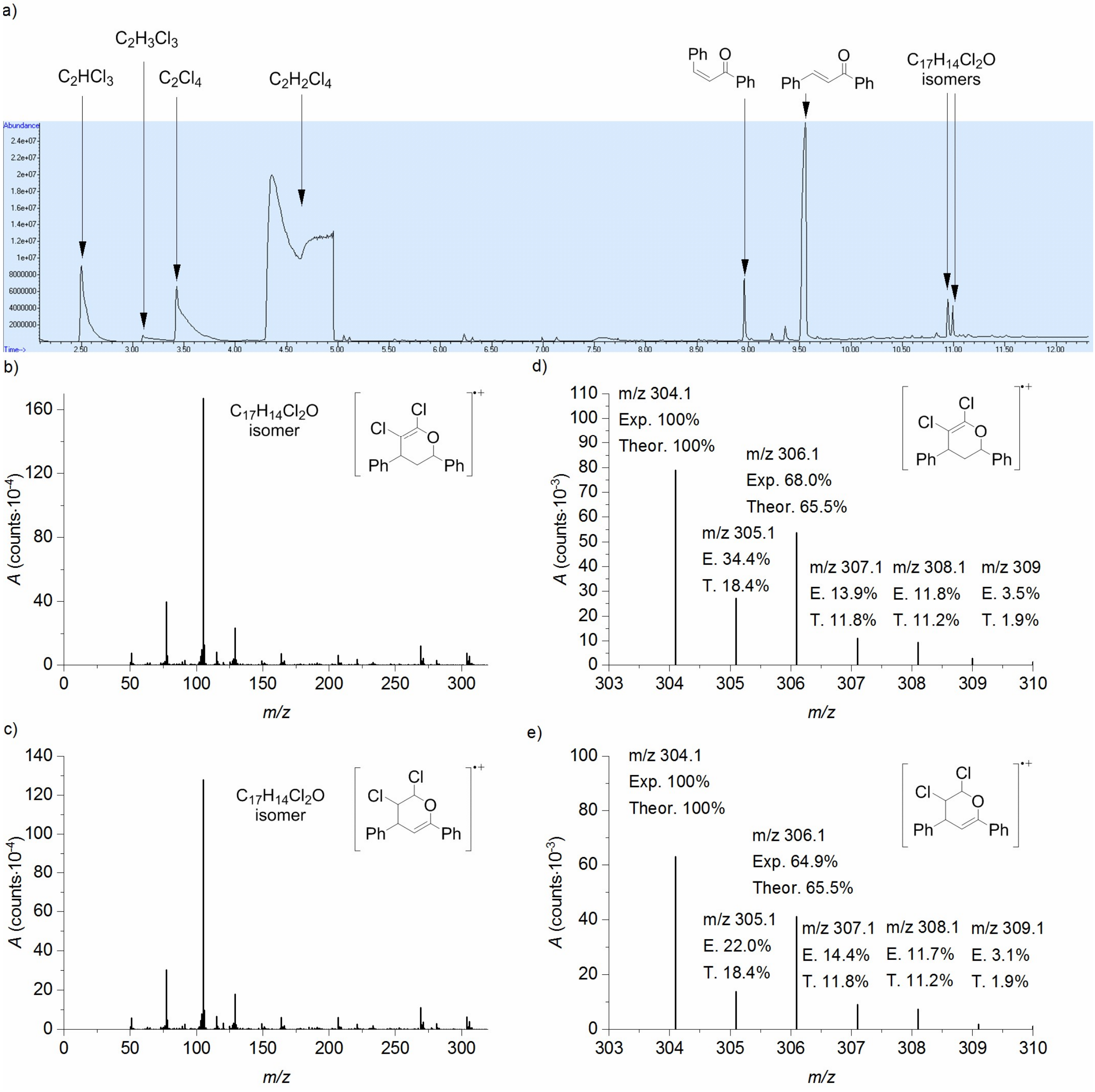

Supplementary Figure 118 - GC-MS data of the reaction mixture using 1,1,2,2-tetrachloroethane as solvent. a) Gas chromatogram. b) Mass spectrum of the GC peak (retention time 10.950 min) corresponding to the compound with brutto formula $C_{17}H_{14}Cl_2O$ radical cation. One of possible chemical structures is shown. c) Zoomed in mass spectrum of $C_{17}H_{14}Cl_2O$ radical cation. d) Mass spectrum of the GC peak (retention time 10.996 min) corresponding to the compound with brutto formula $C_{17}H_{14}Cl_2O$ radical cation. One of possible chemical structures is shown. e) Zoomed in mass spectrum of $C_{17}H_{14}Cl_2O$ radical cation.

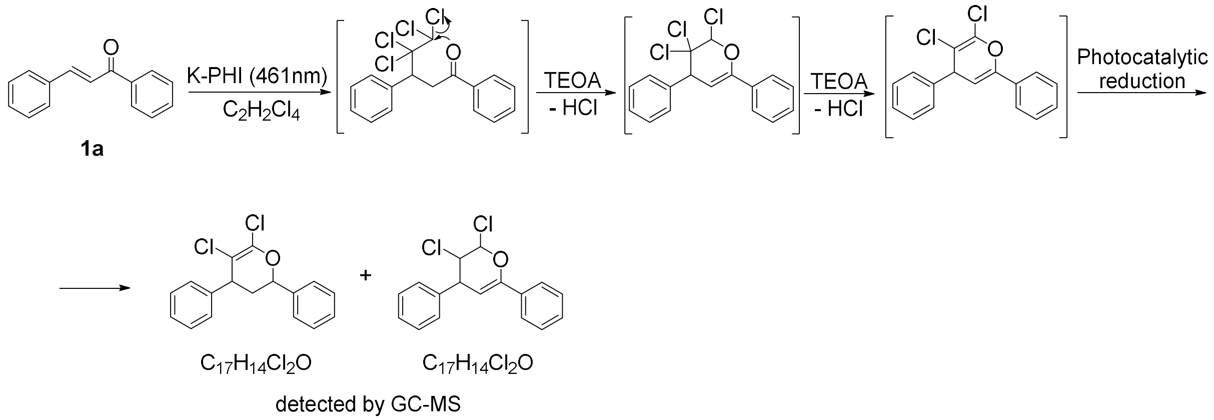

Supplementary Figure 119 – Proposed mechanism using 1,1,2,2-tetrachloroethane as solvent

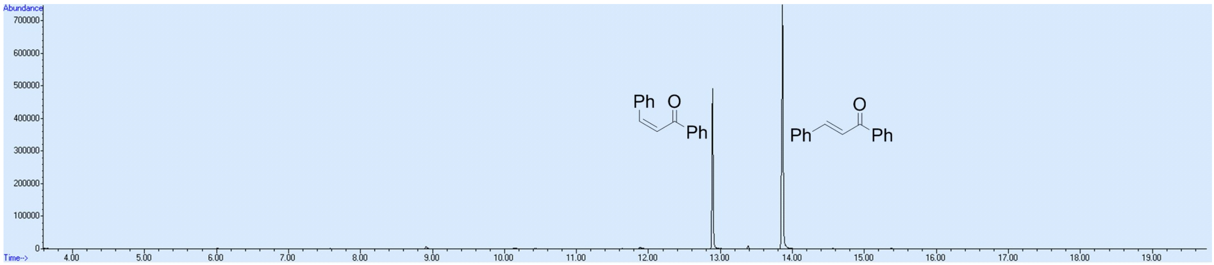

Supplementary Figure 120 - GC-MS data of the reaction mixture using tetrachloromethane as solvent.

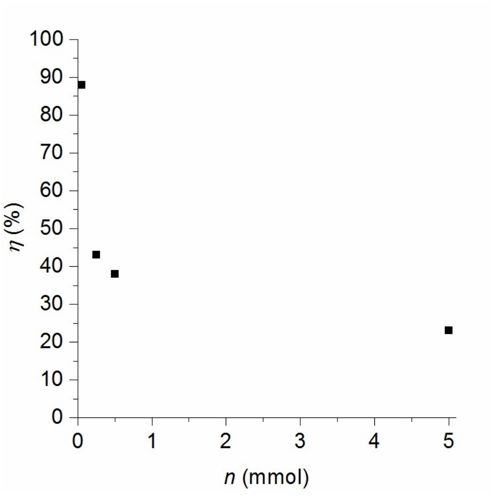

Supplementary Figure 121 – Yield of **2a** varying substrate loading in scale- up experiments

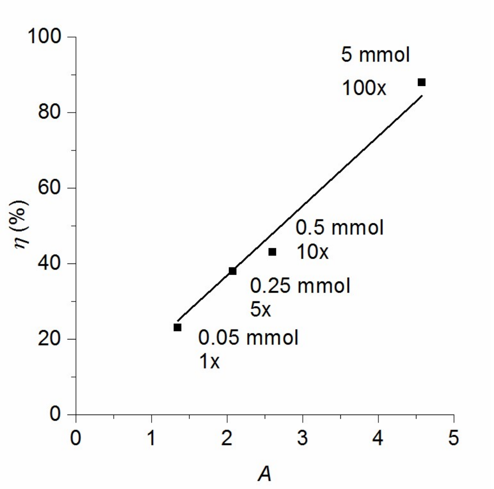

Supplementary Figure 122 – Yield of **2a** varying surface-volume ratio (A)

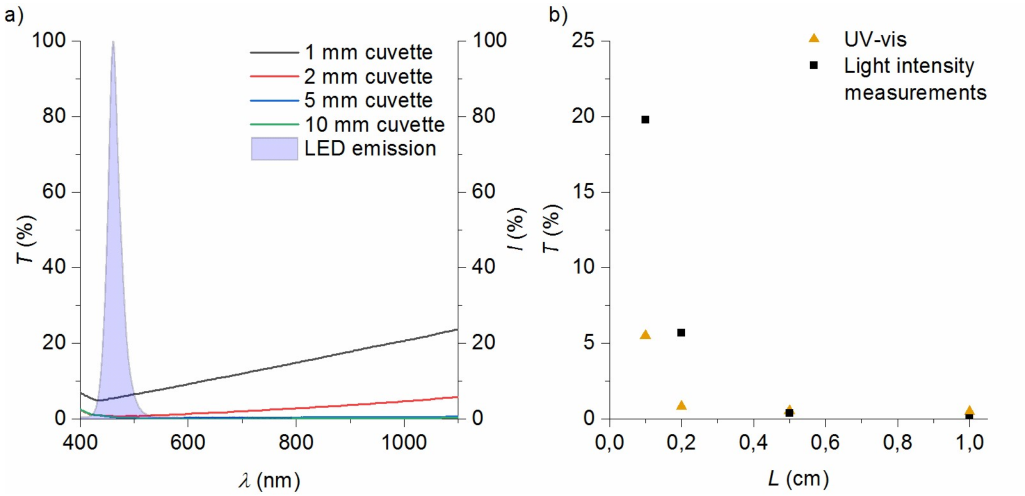

Supplementary Figure 123 – Light penetration tests. a) Transmittance (*T*) of the K-PHI suspension in $CHCl_3$:DMSO (9:1) versus incident light wavelength measured using UV-vis spectrometer and LED emission spectrum (used in the photocatalytic experiments in this work); b) Transmittance (*T*) of the K-PHI suspension in $CHCl_3$:DMSO (9:1) at λ = 461 nm versus cuvette optical path. Triangles denote data points obtained using UV-vis spectrometer. Squares denote data points obtained by measuring fraction of light (λ = 461±20 nm, I_0 = 10.6 mW cm^{-2}) from the external source passed through the cuvette filled with K-PHI suspension.

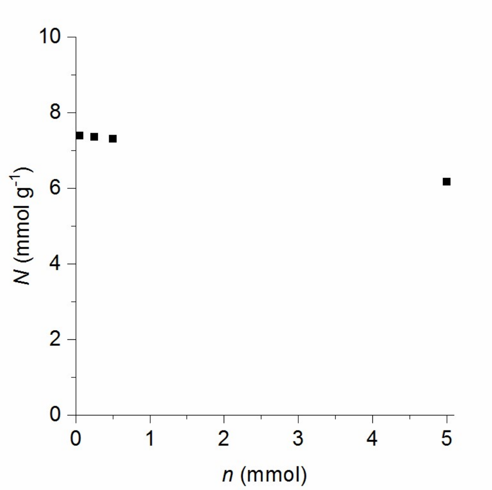

Supplementary Figure 124 – Specific absolute yield (N) of 2a varying substrate loading

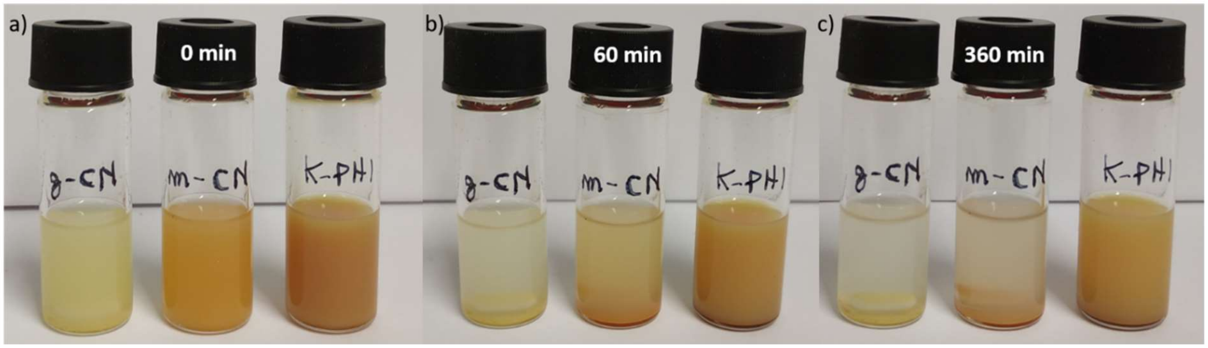

Supplementary Figure 125 – Suspension stability test for different photocatalysts. From left to right, respectively, g-C_3N_4, mpg-CN, K-PHI. Pictures taken after different times: a) immediately after stop stirring; b) after 60 minutes; c) after 360 minutes

Supplementary References

1. Pieber B, Shalom M, Antonietti M, Seeberger PH, Gilmore K. Continuous Heterogeneous Photocatalysis in Serial Micro-Batch Reactors. *Angew. Chem., Int. Ed.* **57**, 9976-9979 (2018).

2. Savateev A, Dontsova D, Kurpil B, Antonietti M. Highly Crystalline Poly(heptazine imides) by Mechanochemical Synthesis for Photooxidation of Various Organic Substrates Using an Intriguing Electron Acceptor – Elemental Sulfur. *J. Catal.*, **350**, 203-211 (2017).

3. JulioMontes-Avila, Díaz-Camacho SP, Sicairos-Félix J, Delgado-Vargas F, Rivero IA. Solution-phase parallel synthesis of substituted chalcones and their antiparasitary activity against Giardia lamblia. *Bioorg. Med. Chem.* **17**, 6780-6785 (2009).

4. Hayat F, Salahuddin A, Umar S, Azam A. Synthesis, characterization, antiamoebic activity and cytotoxicity of novel series of pyrazoline derivatives bearing quinoline tail. *Eur. J. Med. Chem.* **45**, 4669-4675 (2010).

5. Fang F, Li Y, Tian S-K. Stereoselective Olefination of N-Sulfonyl Imines with Stabilized Phosphonium Ylides for the Synthesis of Electron-Deficient Alkenes. *Eur. J. Org. Chem.*, 1084–1091 (2011).

6. Braun RU, Ansorge M, Müller TJJ. Coupling–Isomerization Synthesis of Chalcones. *Chem. - Eur. J.* **12**, 9081–9094 (2006).

7. Kumar V*, et al.* Novel Chalcone Derivatives as Potent Nrf2 Activators in Mice and Human Lung Epithelial Cells. *J. Med. Chem.* **54**, 4147–4159 (2011).

8. Ranu BC, Jana R. Catalysis by Ionic Liquid. A Green Protocol for the Stereoselective Debromination of vicinal-Dibromides by [pmIm]BF4 under Microwave Irradiation. *J. Org. Chem.* **70**, 8621–8624 (2005).

9. Gottumukkala AL*, et al.* Pd-Diimine: A Highly Selective Catalyst System for the Base-Free Oxidative Heck Reaction. *J. Org. Chem.* **76**, 3498–3501 (2011).

10. Gupta S*, et al.* Pyrimidine as an Aryl C-H Activating Group. *Org. Lett.* **20**, 3745-3748 (2018).

11. Zhang J, Xing C, Tiwari B, Chi YR. Catalytic Activation of Carbohydrates as Formaldehyde Equivalents for Stetter Reaction with Enones. *J. Am. Chem. Soc.* **135**, 8113-8116 (2013).

12. Kurpil B, Markushyna Y, Savateev A. Visible-Light-Driven Reductive (Cyclo)Dimerization of Chalcones over Heterogeneous Carbon Nitride Photocatalyst. *ACS Catal.* **9**, 1531-1538 (2019).

13. Ghosh I, Khamrai J, Savateev A, Shlapakov N, Antonietti M, König B. Organic semiconductor photocatalyst can bifunctionalize arenes and heteroarenes. *Science* **365**, 360-366 (2019).

14. Kurpil B*, et al.* Carbon nitride photocatalyzes regioselective aminium radical addition to the carbonyl bond and yields N-fused pyrroles. *Nat. Commun.* **10**, 945-945 (2019).
